# Advancements in the design and development of pyrazoline-based antimycobacterial agents: an update and future perspectives

**DOI:** 10.1039/d5ra03759j

**Published:** 2025-09-01

**Authors:** Gourav Rakshit, Soumi Chakraborty, Sanjib Bhakta, Venkatesan Jayaprakash

**Affiliations:** a Department of Pharmaceutical Sciences and Technology, Birla Institute of Technology Mesra Ranchi 835215 India venkatesanj@bitmesra.ac.in; b Mycobacteria Research Laboratory, Institute of Structural and Molecular Biology, School of Natural Sciences, Birkbeck, University of London London WCIE 7HX UK s.bhakta@bbk.ac.uk

## Abstract

Pyrazoline scaffolds have attracted significant interest in medicinal chemistry due to their broad spectrum of pharmacological activities. Pyrazole-based drugs are either already approved or are currently undergoing clinical trials across a range of therapeutic areas. Pyrazolines (Δ^2^-pyrazolines or 2-pyrazoline or 4,5-dihydropyrazoles) evolved as cyclic analogues of thioacetazone and were explored for enhanced antitubercular activity over the past five decades. The scope of this review focused on how extensively the chemical space around pyrazolines has been explored in relation to their antitubercular activity, rather than presenting a general structure–activity relationship (SAR) account. In this exercise, we covered key molecular modifications, including rationale substitutions and conjugations, aimed at enhancing the potency in general. Additionally, information pertaining to *in vitro*/*in silico* target interaction and ADMET studies are also covered. A dedicated section is included to showcase target-oriented strategies (InhA, cytochrome P450 14α-sterol demethylase, and enzymes involved in the mycobactin biosynthesis pathway), recent patents, suggested schemes for reported pyrazolines, and an overview of research methodologies and evaluation models. We believe that this review will enable medicinal chemists to map unexplored chemical space in identifying critical research gaps. This is essential for the rational design and development of potent antitubercular agents against tuberculosis (TB), drug-resistant tuberculosis (DR-TB), and other non-tubercular mycobacterial diseases (NTMD).

## Abstract 2

Advancements in the design and development of pyrazoline-based antimycobacterial agents (1975–2025 & beyond).

## Graphical abstract

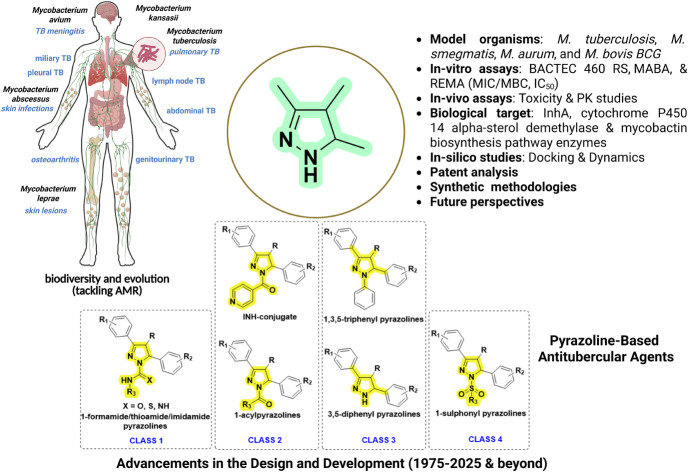

## 1 Introduction

**Fig. 1:**
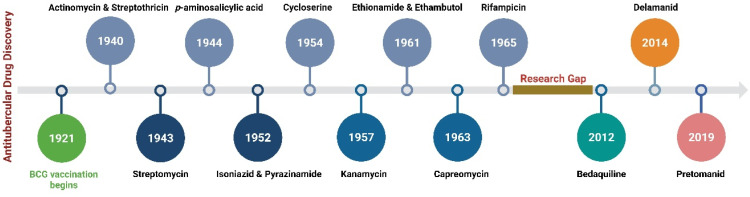
A timeline showing the development of antitubercular drugs and year of their first public use (created in BioRender. Agreement number: ML28OHA80L, Rakshit, G. (2025) https://BioRender.com/f1mvqwy).

**Fig. 2:**
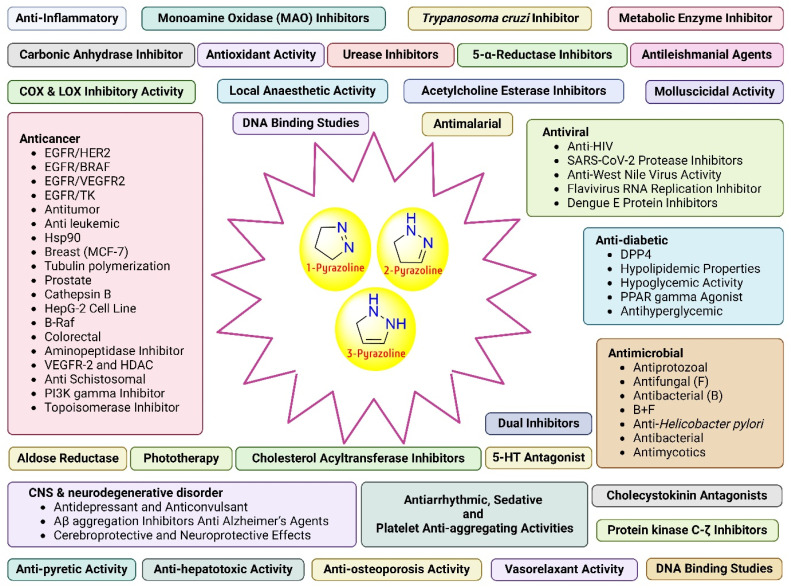
Pictorial representation of the diversified biological activities exhibited by the pyrazoline scaffold (created in BioRender. Agreement number: GW28OHBTT9, Rakshit, G. (2025) https://BioRender.com/ty0umbd).

Tuberculosis (TB) is the leading cause of human death from a single infectious bacterial pathogen, *Mycobacterium tuberculosis* (*Mtb*). According to WHO's Global TB Report 2024, 8.2 million new TB cases were reported globally, with an estimated 1.25 million deaths.^1^ Tubercle bacilli primarily affect the lungs, but it can also lead to systemic infection and spread to other organs of the body. This can result in conditions such as bone TB, TB meningitis, and genital TB.^2,3^ Other non-tuberculous mycobacteria, such as *Mycobacterium avium* (*Mav*) and *Mycobacterium abscessus* (*Mabs*), are notoriously intrinsically resistant and are gaining increasing attention for causing severe respiratory infections, particularly in immunocompromised patients with conditions such as cystic fibrosis, asthma, and COPD.^4^ The growing concern over multidrug resistance in mycobacterial diseases is alarming, as treatment often involves a newly developed regimen, which can last anywhere from six months to two years.^5^ Early detection and effective treatment for TB are crucial to prevent its spread and improve the treatment outcome. The prolonged antibiotic treatment is challenging for patients, especially in low- and middle-income countries, where over 90% of cases and deaths occur.^6^ The golden era of anti-TB drug development, along with recently approved therapeutic agents, is illustrated in Fig. 1. There has been a recent surge in anti-tuberculosis drug discovery, as reflected by the growing number of promising candidates currently in development, including those undergoing clinical trials.^7^ The most prominent classes of drugs currently under investigation in the discovery phase include indazoles, sulphonamides, diaryl thiazoles, aryl sulphonamides, and oxazolidinones. Beyond existing classes, there is a critical need to enrich the discovery pipeline with novel scaffolds. In this context, pyrazolines (Δ^2^-pyrazolines or 2-pyrazoline or 4,5-dihydropyrazoles) emerge as promising candidates due to their potent biological activity and drug-like properties. While pyrazoline ring-containing compound(s) are not a major component in the first-line or second-line anti-TB drugs, some experimental pyrazoline derivatives have shown promising anti-TB activity in preclinical studies. These pyrazoline derivatives may interfere with essential and conditionally essential targets responsible for cell wall synthesis, protein synthesis, nucleic acid synthesis, energy metabolism of mycobacteria, and/or iron acquisition machinery (led by mycobactin, carboxymycobactin metabolism, trans-membrane transporters and their regulators), making the bacteria weak and more vulnerable to host defences. These five-membered heterocycles, characterised by two adjacent nitrogen atoms (N–N bond), exist in three isomeric forms, 1-pyrazoline, 2-pyrazoline, and 3-pyrazoline, and are known for their ability to exhibit a wide range of biological effects (Fig. 2).^8–31^ Several clinically approved drugs feature the pyrazole scaffold, including axitinib (for metastatic renal cell carcinoma), ibrutinib (for chronic lymphocytic leukaemia), and ibipinabant (a CB1 receptor inverse agonist), as shown in Tables 1 and 2. Additionally, various nonsteroidal anti-inflammatory drugs (NSAIDs), such as antipyrine, aminophenazone, phenylbutazone, and metamizole, also incorporate this scaffold. Their potent and diversified biological activities, along with immense prospects for structural modification, position pyrazolines as privileged scaffolds in modern drug design and discovery. Notably, concerning pyrazolines, extensive research has been dedicated to their development as antitubercular agents, with particular emphasis on 3,5-disubstituted pyrazolines, 1,3,5-trisubstituted derivatives, and their drug conjugates.

**Table 1:** Tabular representation of some pyrazole-based drugs approved across various therapeutic areas

Sl no.	Marketed (approved)			
	Drug name	Structure	Inventor	Therapeutic area
1	Celecoxib	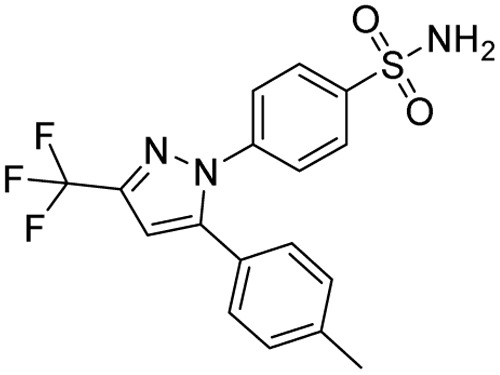	G. D. Searle & Co: now Pfizer Inc.	Anti-inflammatory & analgesic
2	Phenylbutazone	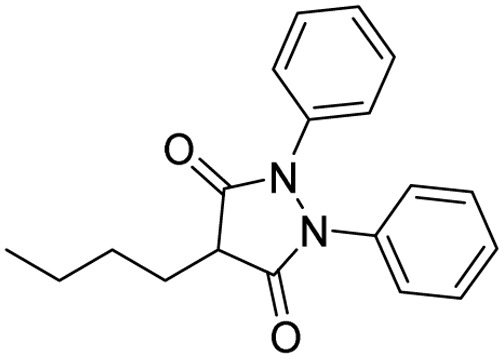	Geigy, merged into Novartis	
3	Antipyrine	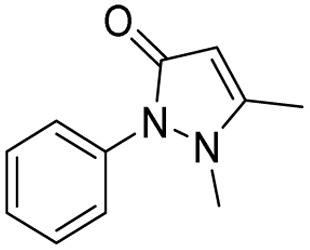	Friedrich Bayer & Co: now Bayer AG	
4	Riociguat	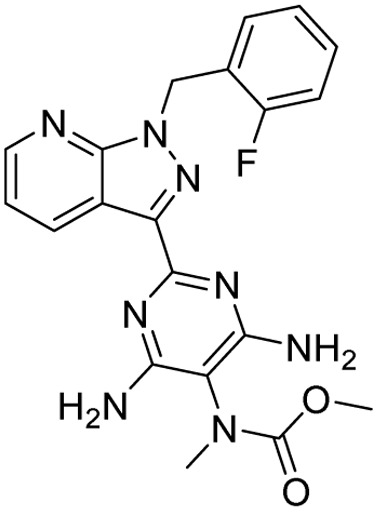	Bayer AG	Cardiovascular & pulmonary
5	Berotralstat	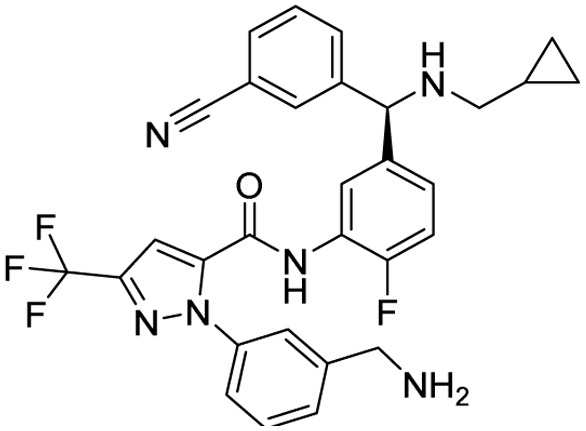	BioCryst pharmaceuticals	
6	Ibrutinib	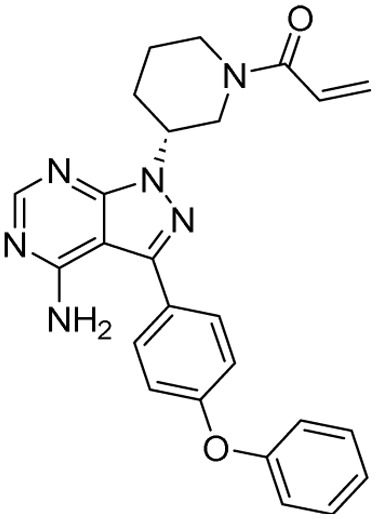	Johnson & Johnson's Janssen Biotech	Oncology (cancer)
7	Ruxolitinib	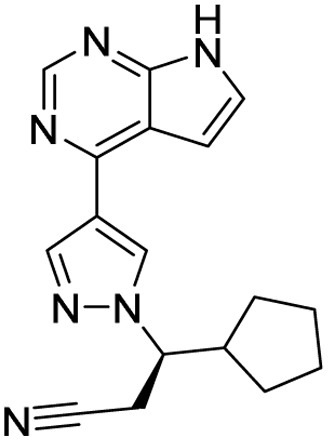	Incyte Corporation (U.S.)	
8	Axitinib	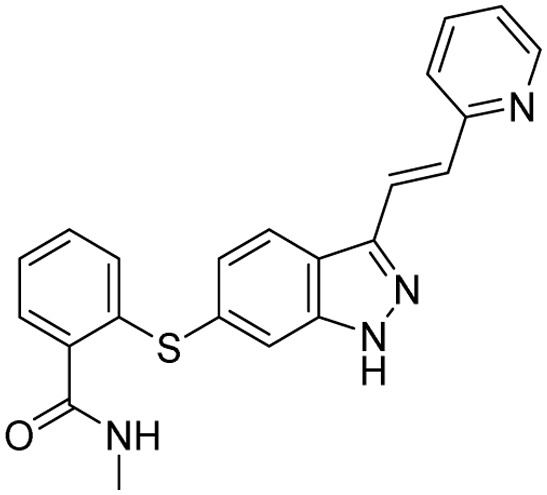	Pfizer	
9	Niraparib	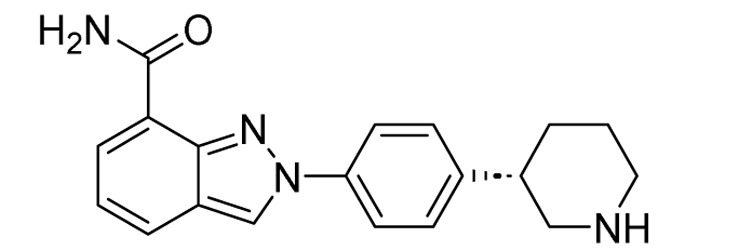	GlaxoSmithKline	
10	Baricitinib	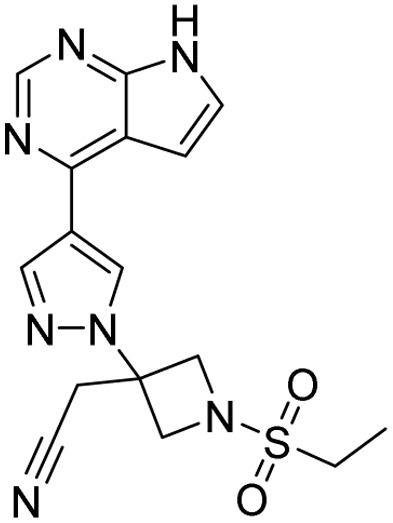	Eli Lilly and Company	
11	Futibatinib	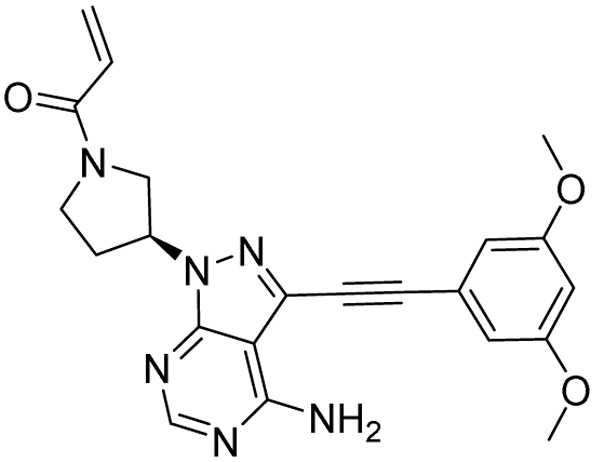	Taiho Oncology	
12	Pralsetinib	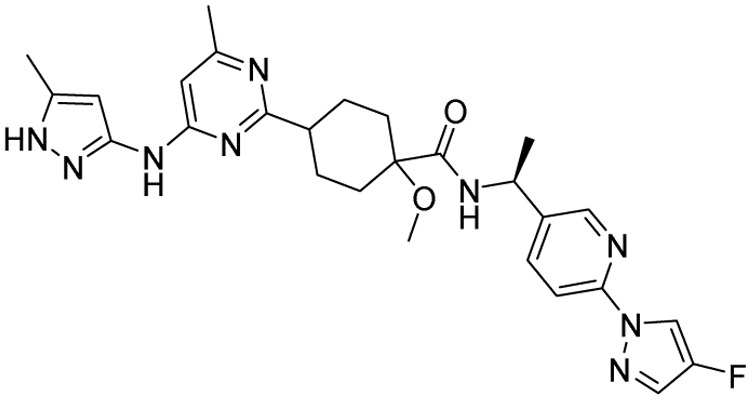	Roche/Genentech	
13	Selpercatinib	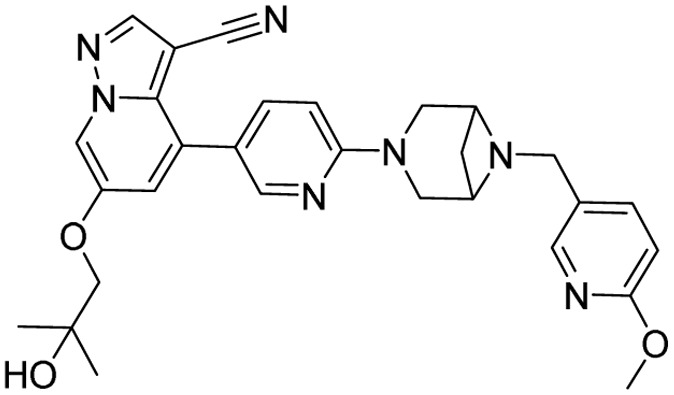	Loxo Oncology	
14	Lenacapavir	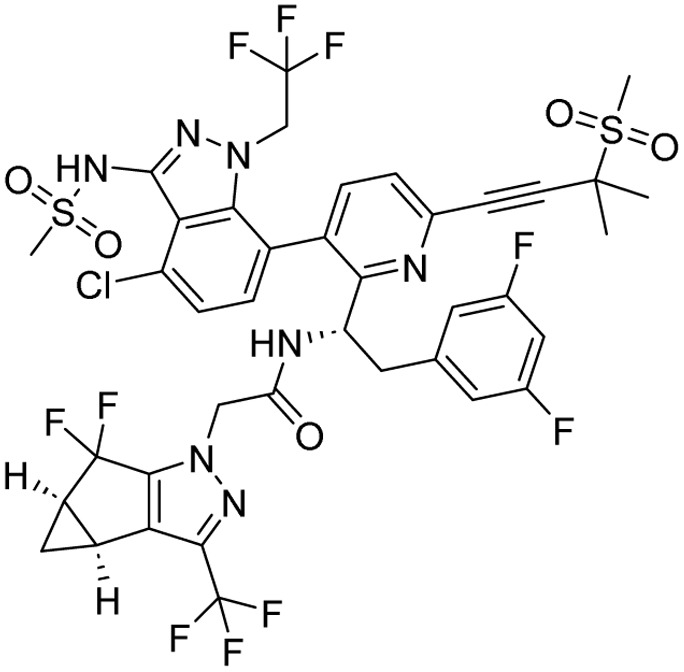	Gilead Sciences	Infectious disease
15	Cefoselis	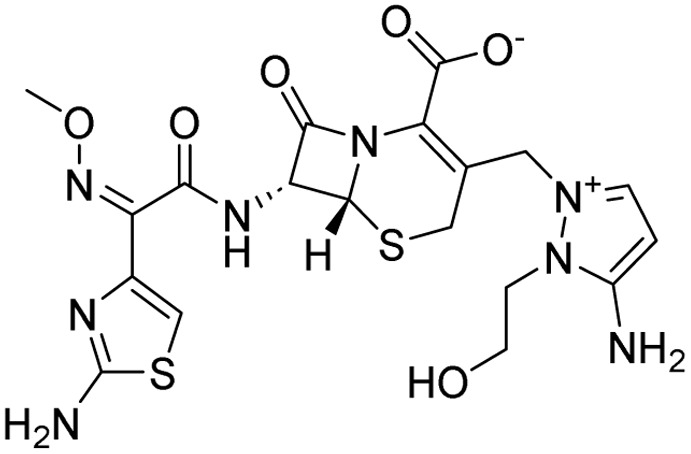	Hoffmann-La Roche	
16	Ceftolozane	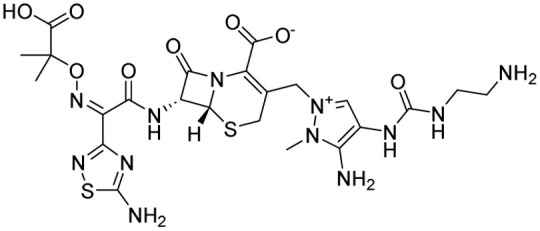	Merck & Co., Inc.	
17	Zavegepant	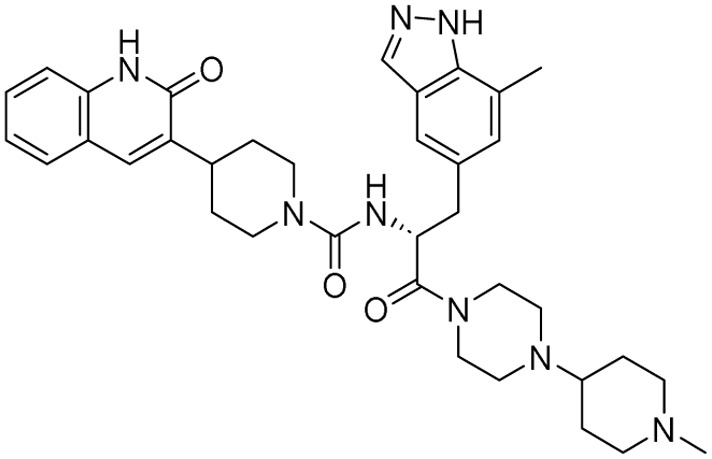	Biohaven pharmaceuticals: now Pfizer Inc.	Neurology & psychiatry
18	Omidenepag isopropyl	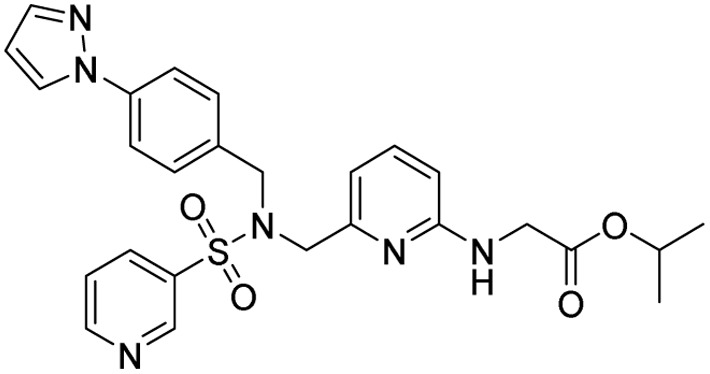	Ube Industries Ltd	Ophthalmology
19	Rimonabant	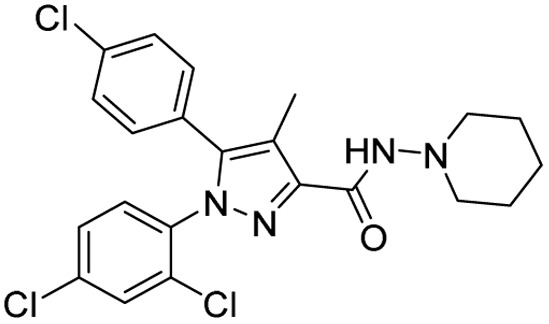	Sanofi-Aventis	Anti-obesity agent

**Table 2:** Tabular representation of some pyrazole-based drugs under clinical trials across various therapeutic areas

Under trials			
Drug name	Structure	Inventor	Therapeutic area
PF-03715455	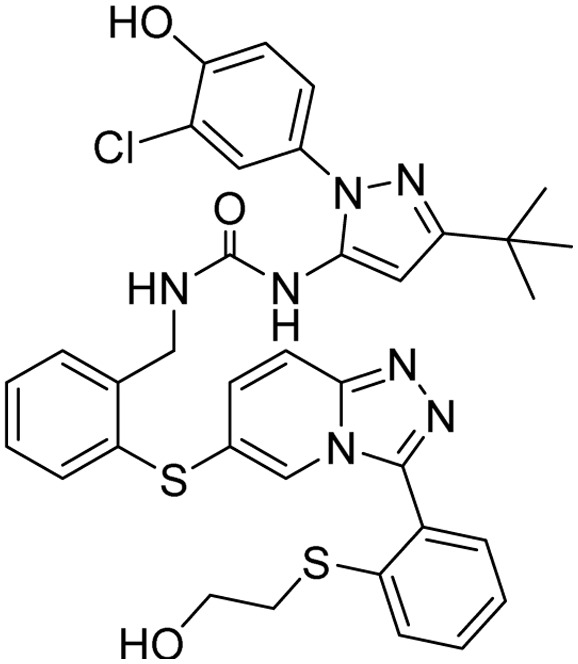	Pfizer Inc.	Respiratory (asthma, COPD)
PF-03882845	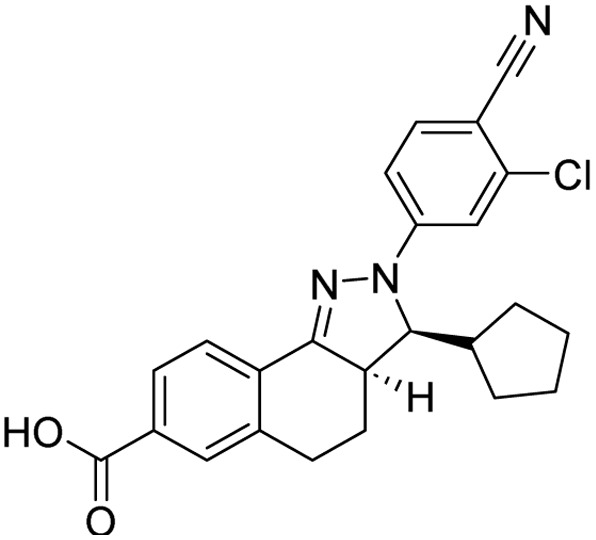	Pfizer Inc.	Type 2 diabetes, diabetic nephropathy
GDC-0941 (pictilisib)	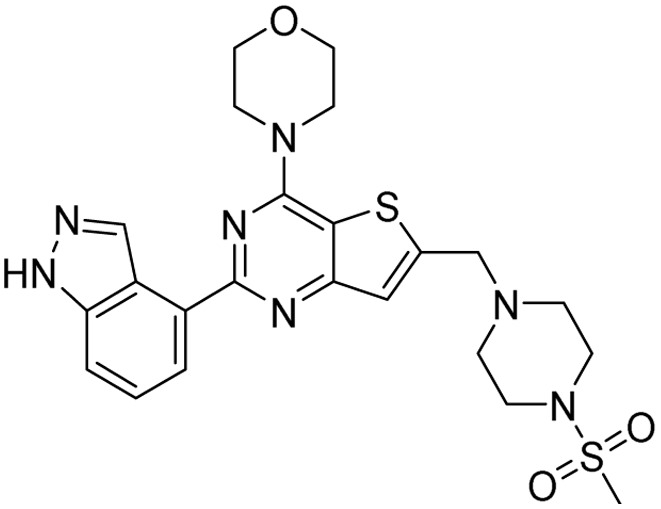	Genentech: Roche Group	Oncology (cancer)
TB47	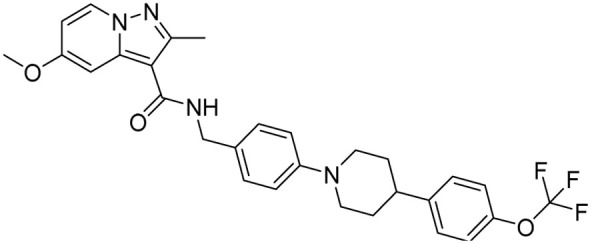	Guangzhou Institute of Respiratory Medicine Company Limited	Anti-tubercular
CLB073	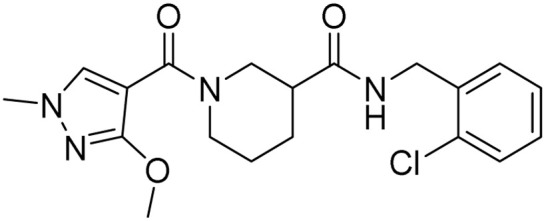	Bill & Melinda Gates Medical Research Institute	
CDPPB	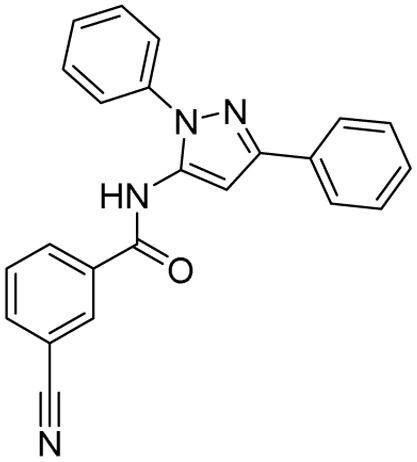	Vanderbilt University, Nashville, Tennessee	Neurology & psychiatry

**Fig. 3:**
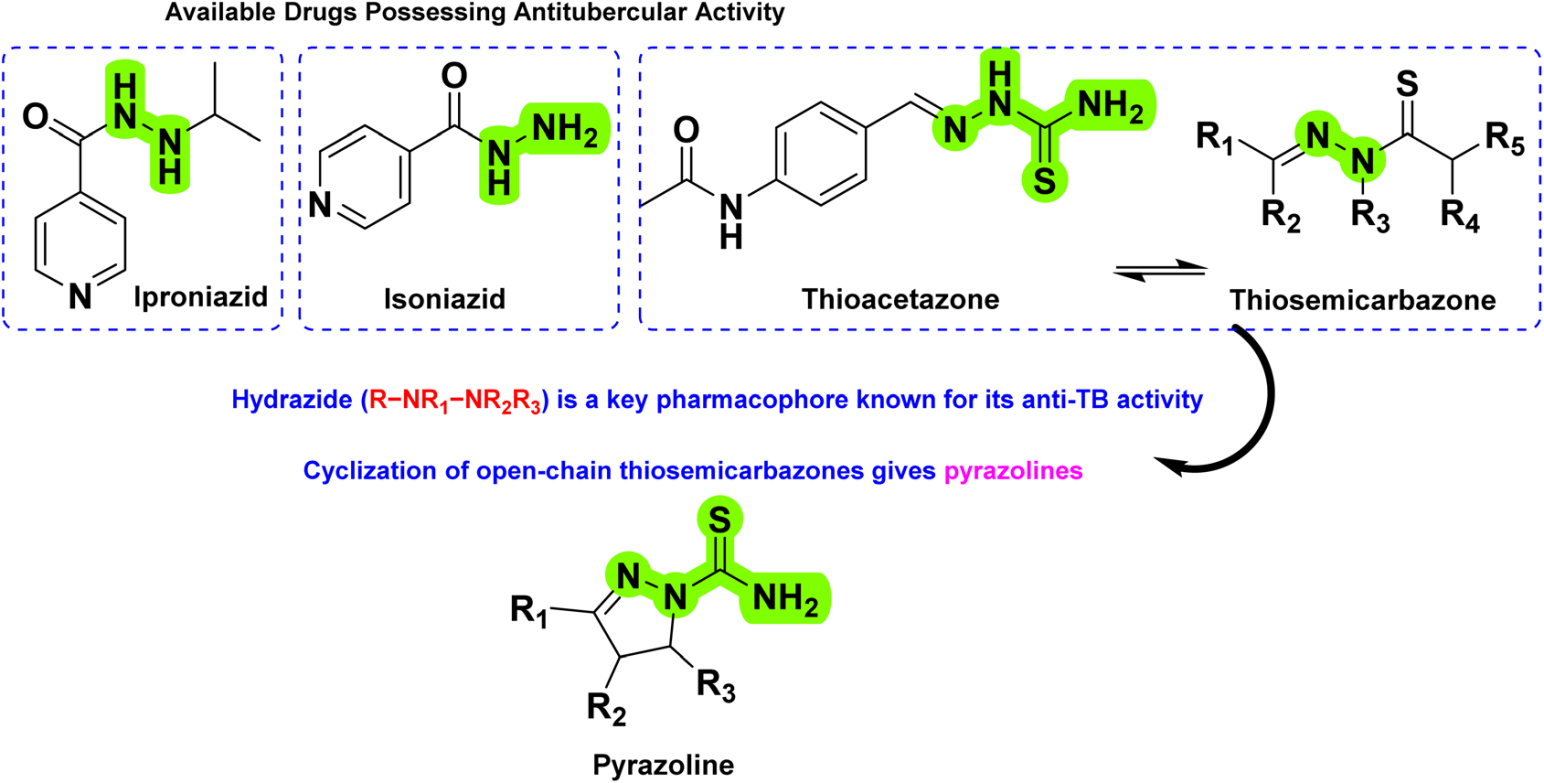
Design strategy for developing pyrazolines as antitubercular agents. The green colour highlights the essential functional groups responsible for antibacterial/tubercular activity.

Isoniazid (INH), first synthesized in 1912, remains a first-line drug for the treatment of tuberculosis.^32^ Its chemical structure features a pyridine ring (a six-membered aromatic ring with one nitrogen atom) and an acyl hydrazide group (–CO–NH–NH_2_) attached to the ring. The hydrazide group is essential for its antibacterial activity, enabling the drug to interact with bacterial enzymes, particularly in *Mtb*. INH inhibits mycolic acid synthesis, a critical component of the bacterial cell wall, effectively weakening the pathogen.^33^ Another drug, amithiozone (also known as thioacetazone (TBI/698) or *p*-acetaminobenzaldehyde thiosemicarbazone), is a second-line oral antibacterial used in the treatment of TB.^34^ Although it has weak activity against *Mtb*, its primary role is to prevent the development of resistance to first-line anti-TB drugs such as INH, rifampicin (RIF), and ethambutol (ETH).^35^ Structurally, thioacetazone belongs to the thiosemicarbazone (–CS–NH–N=) class of compounds. The presence of the hydrazide group (–NH–N) in INH and TBI/698 is crucial for their activity, as they interact with mycobacterial enzymes, inhibiting mycolic acid biosynthesis. Exploration of these compounds' structure–activity relationship (SAR) has highlighted the potential of the nitrogen-rich hydrazide (R–NR_1_–NR_2_R_3_) segment as an effective anti-TB scaffold.^36^ In line with these findings, efforts were directed toward designing novel scaffolds by incorporating a hydrazide pharmacophore in cyclic structure(s). In 1975, Arthur and his group attempted for the first time the anti-TB evaluation of cyclic analogues of thiosemicarbazones, pyrazolines.^37^ Since then, the pyrazoline scaffold has become an attractive pharmacophore for medicinal chemists working in the area of anti-TB drug design and development (Fig. 3).

### 1.1 The overall process of literature review/search

**Fig. 4:**
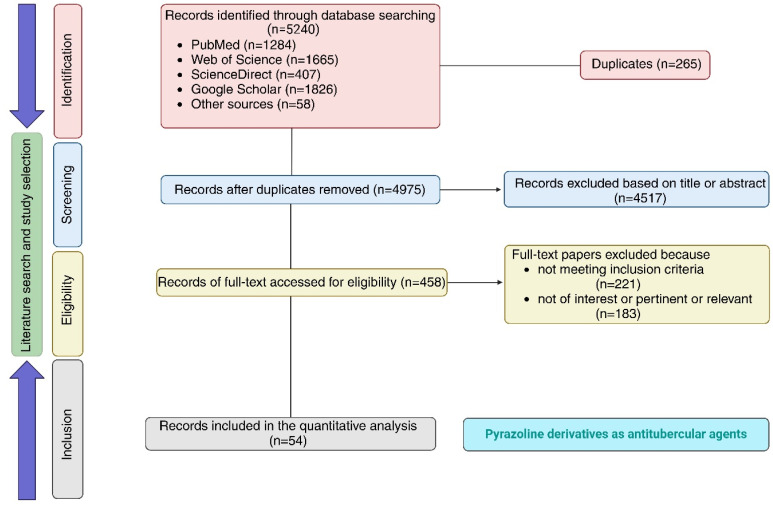
PRISMA flow diagram of literature search and study selection (created in BioRender. Agreement number: JT28OHB3Z3, Rakshit, G. (2025) https://BioRender.com/zlwg4qw).

This review is an attempt to summarize systematically the chemical space around pyrazolines reported for their anti-TB activity over the past 50 years (1975–2025). The literature search strategy (bibliographic research) and study selection process were done according to PRISMA (preferred reporting items for systematic review and meta-analysis) guidelines and are presented in Fig. 4. This process ensures a comprehensive and relevant dataset for our bibliometric analysis. Following these guidelines, we have detailed our search strategy, including the databases used, search terms employed, and inclusion *n* criteria considered during the process. The search terms and keywords for the study selection were pyrazolines OR pyrazoline AND tuberculosis OR anti-tubercular activity AND substituted pyrazoline. The search term was kept broad and limited to a single word to ensure all articles related to pyrazolines were included, minimizing the risk of missing relevant articles. Further exclusions were made based on specific exclusion criteria. The search was carried out for the last 50 years (1975 to 2024) of publications. Two independent reviewers (Gourav Rakshit and Soumi Chakraborty) conducted the literature search in the scientific databases and assessed/verified the eligibility of the studies based on the title, abstract, and anti-tubercular activity. Disagreement was sorted out through consultation with the supervisors (Venkatesan Jayaprakash and Sanjib Bhakta) to reach a consensus. The inclusion criteria were (i) studies involving pyrazoline derivatives as antitubercular agents, (ii) antitubercular evaluation of the designed and synthesized pyrazoline derivatives, (iii) studies on pyrazoline derivatives and their effects on *in vitro* or *in vivo* models, (iv) *in silico* studies conducted to establish a plausible mechanism of action for antitubercular activity and (v) studies published from 1975 to 2025 (50 years, both years included). The exclusion criteria were (i) studies not involving pyrazoline derivatives and their evaluation as antitubercular agents, (ii) studies on systematic reviews, meta-analysis, and case reports, (iii) studies involving clinical data and human subjects, and (iv) published articles in a language different from English. A total of 5240 published records were identified from the database search (PubMed, Web of Science, Scopus, Google Scholar) and other sources. After removing 265 duplicate articles, 4975 papers were screened, and 4517 were excluded based on title and/or abstract. The full text of eligible studies (*n* = 458) was read, and 404 articles were excluded because they did not meet the inclusion criteria (*n* = 221) or were not of interest/pertinent/relevant (*n* = 183). At the end of the selection process, 54 papers were finally selected and included in the study. The number of studies related to newly designed and synthesized pyrazolines evaluated for anti-tubercular activity available is limited, as very few research groups are continuously exploring this area.

### 1.2 Synthetic strategies for the preparation of pyrazolines

The various synthetic methods for pyrazolines have been rigorously reviewed by Matiadis (2023), and readers are encouraged to refer to the review for a comprehensive understanding of the different approaches for the synthesis of pyrazolines.^38^ In this segment, we highlight the essence of the above review, while detailed procedures for the synthesis of pyrazolines covered under this review are provided in the SI.

The most prominent synthetic methodologies for pyrazoline derivatives are:

#### 1.2.1. 1,3-Dipolar cycloadditions

Originally introduced by Rolf Huisgen around 60 years ago to synthesise a wide range of heterocyclic compounds.^39,40^ In this, a 1,3-dipole combines with a dipolarophile to yield a five-membered ring structure. Compared to the traditional hydrazine α,β-enone approach for synthesizing pyrazolines, this method provides enhanced regioselectivity. This reaction proceeds *via* a concerted mechanism, forming two new σ-bonds and substituted pyrazoline rings. Usually carried out under ambient or mild heating conditions and often without the need for a catalyst, this method is valued for its operational simplicity, broad substrate scope, and environmentally friendly profile. This can be further categorized into two subsections based on the type of dipole involved in the reaction.

##### 1.2.1.1 Nitrile imines (Nis) as 1,3-dipoles

**Fig. 5:**
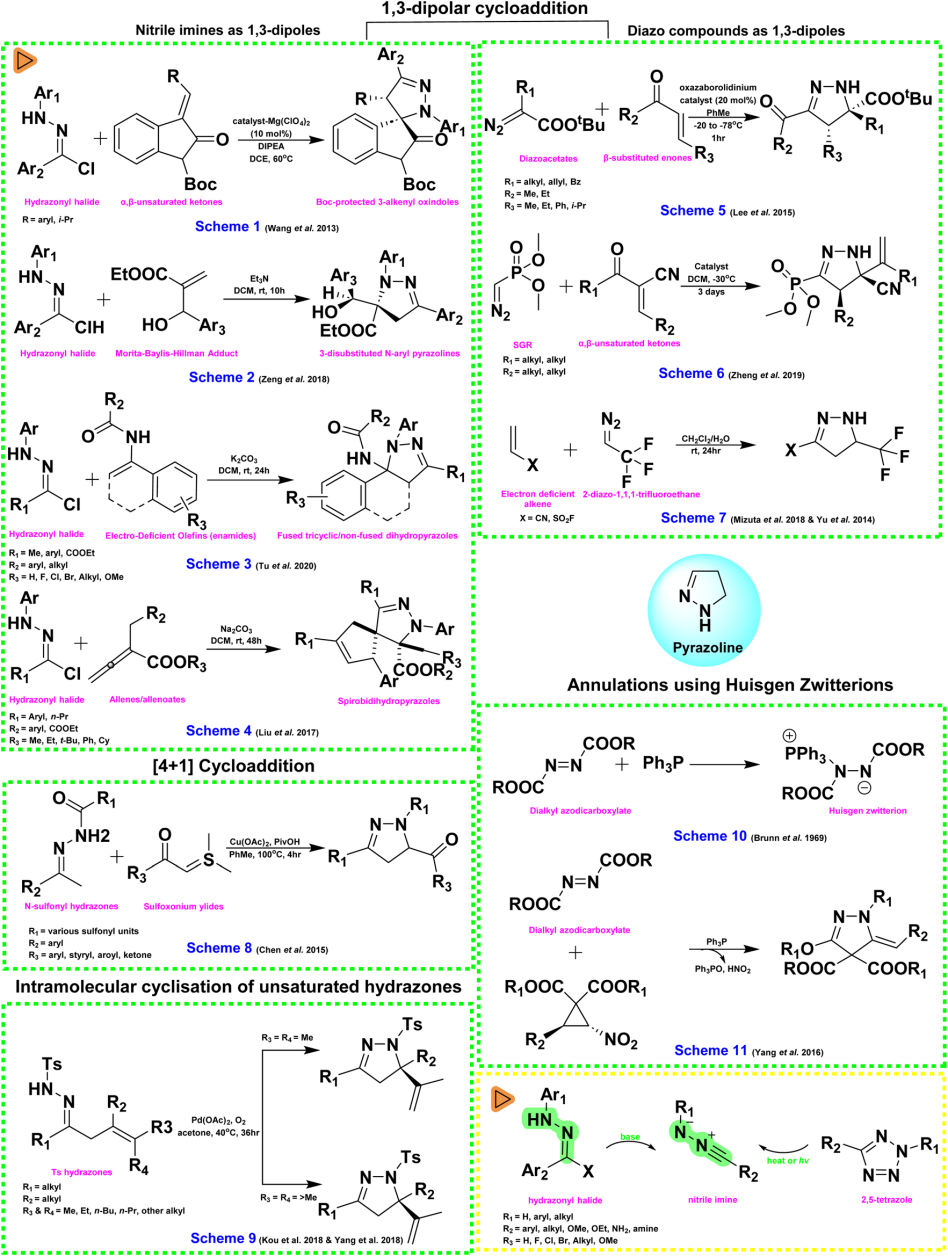
Overview of synthetic strategies for the preparation of pyrazolines (Schemes 1–7: 1,3-dipolar cycloadditions, Scheme 8: [4 + 1]-cycloadditions, Scheme 9: intramolecular cyclisation of unsaturated hydrazones, and Schemes 10 and 11: annulations using Huisgen zwitterions).

Nis reacts with alkenes and alkynes to form five-membered nitrogen-containing heterocycles. Due to their inherent instability, NIs are usually generated *in situ* from suitable precursors. The most commonly used precursors include hydrazonyl halides and 2,5-tetrazoles. *E.g.*,

• Non-spirocyclic pyrazolines can be synthesized *via* cycloaddition of difluoroacetohydrazonoyl bromides with α,β-unsaturated ketones using K_2_CO_3_ as the base. Pyrazoline spiroadducts with high yield and enantioselectivity were formed by the dipolar cycloaddition of hydrazonyl chlorides with Boc-protected 3-alkenyl oxindoles, promoted by a Mg(ClO_4_)_2_-based catalyst (Fig. 5; Scheme 1).^41^

• 3-Disubstituted *N*-aryl pyrazolines can be synthesized by reacting nitrile imines with Morita–Baylis–Hillman (MBH) adducts (Fig. 5; Scheme 2).^42^

• Cycloaddition between nitrile imines and enamides, forming fused tricyclic or non-fused pyrazoline with a quaternary center at C-5. The method shows broad substrate scope, functional group tolerance, gram-scale synthesis, one-pot conversion to pyrazoline, and further modification *via* Suzuki coupling (Fig. 5; Scheme 3).^43^

• Nis react with allenoates to produce spirobipyrazolines with excellent yield and diastereoselectivity, using Na_2_CO_3_ as base and a broad substrate scope (Fig. 5; Scheme 4).^44^

##### 1.2.1.2 Diazo compounds as 1,3-dipoles

The synthesis of 2-pyrazolines in most protocols involves the use of chalcone derivatives or other α,β-unsaturated ketones reacting with diazoalkanes.

###### Diazoacetates

Using a chiral oxazaborolidinium (cation) catalyst, functionalized pyrazolines were synthesized *via* 1,3-dipolar cyclization of β-substituted enones with substituted diazoacetates (Fig. 5; Scheme 5).^45^

###### α-Diazophosphonates

Seyferth–Gilbert reagent (SGR) and BOR (Bestmann–Ohira reagent), initially used to convert carbonyls to alkynes, served as diazoacetate analogues in 1,3-dipolar cycloadditions to synthesize pyrazolines.^46,47^ A chiral catalyst from silver carbonate and spirobiindane-based phosphoric acid enabled the 1,3-dipolar cycloaddition of SGR with α,β-unsaturated ketones, yielding chiral phosphonylpyrazolines (Fig. 5; Scheme 6).^48^

###### Di- and trifluorodiazomethanes

Trifluorodiazoethane (CF_3_CHN_2_) is a widely used diazoalkane for introducing trifluoromethyl groups into heterocycles. In a pioneering study, Mykhailiuk *et al.* reported a one-pot, base-free synthesis of CF_3_-substituted pyrazolines from electron-deficient alkenes, yielding 5-carboxylate 2-pyrazolines in high yields under mild conditions (Fig. 5; Scheme 7).^49,50^

#### 1.2.2. [4 + 1]-Cycloadditions

Recently, [4 + 1]-annulation reactions have emerged as an alternative to 1,3-dipolar cyclizations. The main approach involves using 1,2-diaza-1,3-dienes (DDs), generated *in situ* from α-haloketohydrazones or α-hydroxyl ketohydrazones, and sulfur or sulfoxonium ylides. These ylides act as one-carbon units reacting with DDs in formal [4 + 1]-annulation reactions.^51^ Chen *et al.* synthesized functionalized pyrazolines by reacting sulfoxonium ylides with *in situ* generated DDs from *N*-tosylhydrazones, using Cu(OAc)_2_ and pivalic acid. Notably, they avoided α-halo hydrazones in DD formation (Fig. 5; Scheme 8).^52^

#### 1.2.3. Intramolecular cyclisation of unsaturated hydrazones

Unsaturated hydrazones undergo metal-catalyzed intramolecular cyclization *via* radical or ionic pathways, often using palladium catalysts. This triggers cascade reactions, yielding highly functionalized and structurally diverse pyrazolines, sometimes with high enantioselectivity. Zhang *et al.* developed a Pd(OAc)_2_-catalyzed cyclization of *N*-Ts hydrazones using a chiral pyridine-oxazoline ligand, yielding products with exocyclic alkenes and excellent enantioselectivity. The reaction generated two vicinal stereocenters and showed regioselectivity based on alkyl chain length, forming either α,β- or β,γ-unsaturated products (Fig. 5; Scheme 9).^53,54^

#### 1.2.4. Annulations using Huisgen zwitterions

Huisgen zwitterions, formed *in situ* (Fig. 5; Scheme 10) from triphenylphosphine and dialkyl azodicarboxylates, are key intermediates in the Mitsunobu reaction. Renewed interest, especially from Nair's studies, has led to their use in synthesizing benzoxadiazoles, spiroxadiazolines, 2-pyrazolines. Yang *et al.* reported a novel method for synthesizing 3-alkoxy derivatives, where zwitterions reacted with nitrocyclopropane carboxylates to afford 4-arylidene pyrazolines with high diastereoselectivity (Fig. 5; Scheme 11).^55,56^

#### 1.2.5. Iodine-mediated intramolecular 5-*exo*-trig cyclization

5-*exo*-trig cyclization is a term derived from Baldwin's rules, which classify ring-closing reactions based on the size of the ring formed and the nature of the bond formation. In this, “5” refers to the formation of a five-membered ring. The term “*exo*” indicates that the new bond is formed outside the original chain, meaning the nucleophile attacks a position external to the starting framework. Finally, “trig” denotes that the electrophilic centre involved in the cyclization is trigonal, typically an sp^2^-hybridized atom such as one found in a double bond or a π-system like an alkene or carbonyl carbon. Together, these terms describe a favoured pathway for forming five-membered rings *via* intramolecular nucleophilic attack on an sp^2^ centre.

**Fig. 6:**
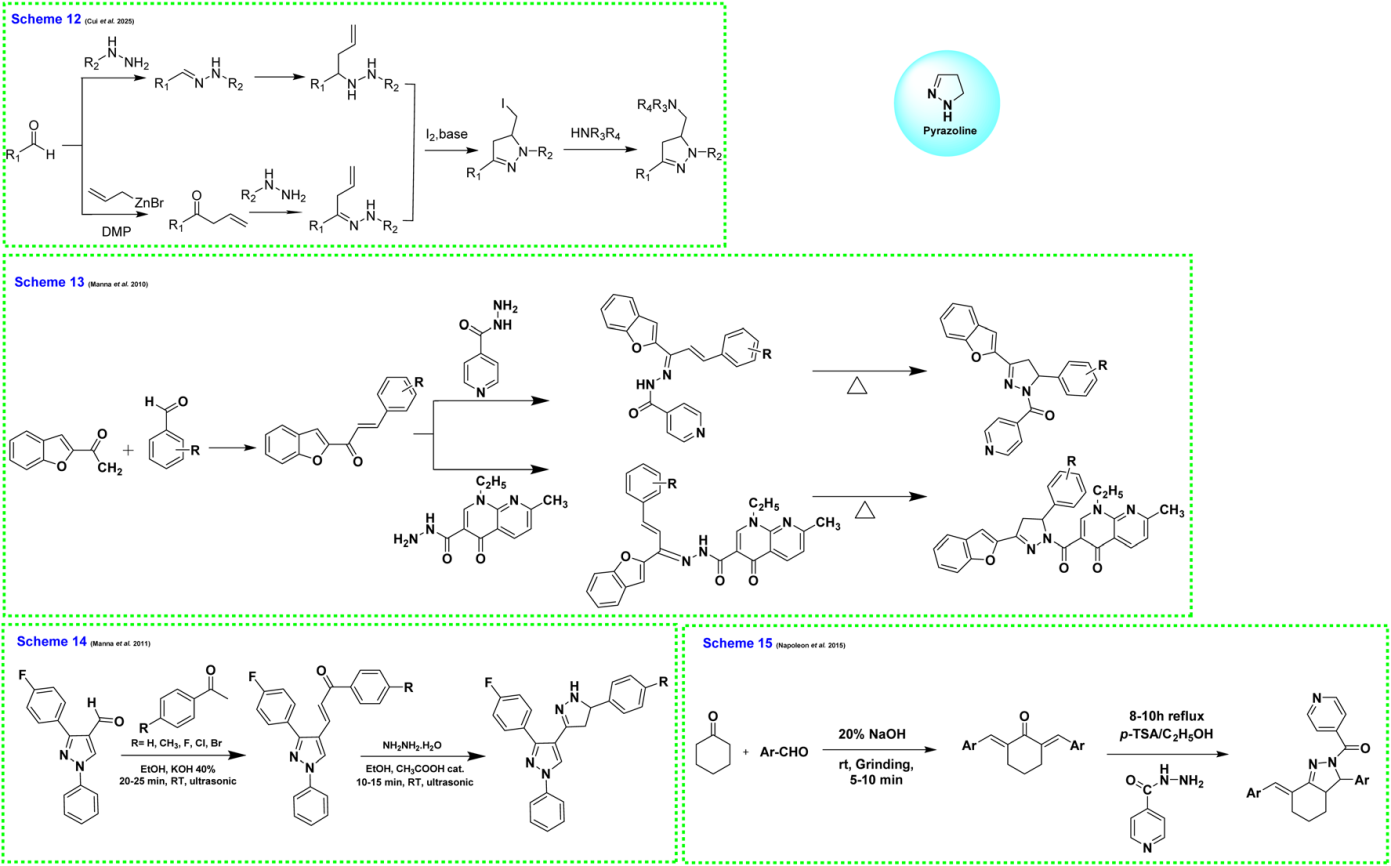
Various synthetic approaches for the synthesis of pyrazoline derivatives (Scheme 12: iodine-mediated intramolecular 5-*exo*-trig cyclization, Scheme 13: microwave-assisted synthesis, Scheme 14: ultrasonic irradiation-assisted synthesis, and Scheme 15: mechanochemical (grinding-based) synthesis).

In 2025, Cui and colleagues synthesized 2-pyrazoline derivatives through an iodine-mediated intramolecular 5-*exo*-trig cyclization of homoallyl hydrazines. The reaction was carried out at room temperature using 3 equivalents of iodine and 5 equivalents of sodium bicarbonate, resulting in the selective formation of the 2-pyrazoline products without any side products such as azetidines or pyrrolidines (Fig. 6; Scheme 12).^57^

#### 1.2.6. Advanced and green synthetic approaches

In recent years, there has been remarkable progress in developing green and sustainable methodologies for synthesizing pyrazoline derivatives. These innovative approaches aim to enhance efficiency while minimizing environmental impact:

##### 1.2.6.1 Microwave-assisted synthesis

Microwave irradiation significantly accelerates reaction rates, reduces solvent use, and improves product yields compared to traditional heating methods. For example, combining chalcones with hydrazine derivatives under microwave conditions leads to the rapid and efficient formation of pyrazolines within minutes. This technique offers cleaner reaction profiles, shorter reaction times, and greater environmental compatibility.

In 2010, Manna and Agrawal synthesized pyrazoline derivatives using microwave irradiation, which significantly reduced reaction time and improved yields. Chalcones were first prepared from 2-acetylbenzofuran and aromatic aldehydes, then reacted with isonicotinic acid hydrazide or nalidixic acid hydrazide to yield the final pyrazoline-based compounds. Nalidixic acid hydrazide was obtained from its ester derivative (Fig. 6; Scheme 13).^58^

##### 1.2.6.2 Ultrasonic irradiation

Ultrasound-assisted synthesis utilizes acoustic cavitation to enhance mixing and reaction kinetics, often resulting in high yields and minimal byproduct formation. This energy-efficient method is well-suited for green chemistry applications, offering faster reactions and reduced energy consumption.

In 2011, Manna and Agrawal synthesized 1,3,5-trisubstituted pyrazoline derivatives containing benzofuran and indophenazine moieties. They prepared 5-hydroxy-2-acetylbenzofuran from 2,5-dihydroxybenzaldehyde using chloroacetone and K_2_CO_3_. Key intermediates were formed by reacting substituted benzofurans with various aromatic aldehydes under strong alkaline conditions. The final pyrazoline products were obtained by treating these benzofuran chalcones with glacial acetic acid (Fig. 6; Scheme 14).^59^

##### 1.2.6.3 Grinding-based (mechanochemical) synthesis

Mechanochemical techniques involve physically grinding the reactants, either manually or mechanically, without the use of solvents. This solvent-free, room-temperature approach eliminates the need for hazardous chemicals, making it both cost-effective and environmentally friendly. It is particularly advantageous for resource-limited settings and aligns closely with the principles of sustainable chemistry.

In 2015, Napoleon and his team synthesized bisbenzylidene cycloalkanones by grinding cyclohexanone, aldehydes, and solid NaOH, followed by acid treatment and purification. The resulting intermediates were then refluxed with isoniazid in ethanol using *p*-toluene sulfonic acid as a catalyst. Upon completion, the products were isolated, washed, and recrystallized to obtain the final compounds (Fig. 6; Scheme 15).^60^

#### 1.2.7. Multicomponent reactions (MCRs)

Multicomponent reactions offer a convergent and atom-economical route to pyrazoline derivatives. These one-pot procedures allow for the simultaneous assembly of multiple building blocks under various conditions, including solvent-free, catalyst-free, or with the aid of microwave or ultrasonic irradiation.^61–63^ MCRs are particularly useful for the rapid generation of structural diversity and have been widely applied in the synthesis of bioactive pyrazoline analogues (Table 3).

**Table 3:** Multicomponent synthetic routes for pyrazoline derivatives

Sl. no.	Number of components	Reactants	Catalyst/base/additive	Solvent/conditions	Product type	Key features
1	3	4-Methoxyacetophenone, halogen-substituted benzaldehyde, phenylhydrazine	KOH	Ethanol, microwave irradiation (180 W, 3–6 min)	Pyrazoline derivatives	Simple, rapid, high-yielding; broad substrate scope; products isolated by filtration and washing
2	4	β-Ketoester, hydrazine, aldehyde, malononitrile	None	One-pot, sequential addition	Pyrano[2,3-*c*]pyrazoline	Fused heterocycles; efficient domino process; combines Michael addition, cyclization, and condensation
3	4	Ethyl acetoacetate, hydrazine, tetracyanoethylene (TCE), imidazole	Imidazole (catalytic)	Water (green, aqueous phase)	4-Dicyanomethylene-2-pyrazoline-5-one	Green chemistry, functionalization possible, high yields
4	3	Azo-acetophenone, benzaldehyde, phenylhydrazine	KOH	Ethanol, microwave irradiation	Azo-pyrazoline	Rapid, efficient products isolated by standard workup

### 1.3 The antitubercular activity of pyrazolines: a brief discussion down the lane

**Fig. 7:**
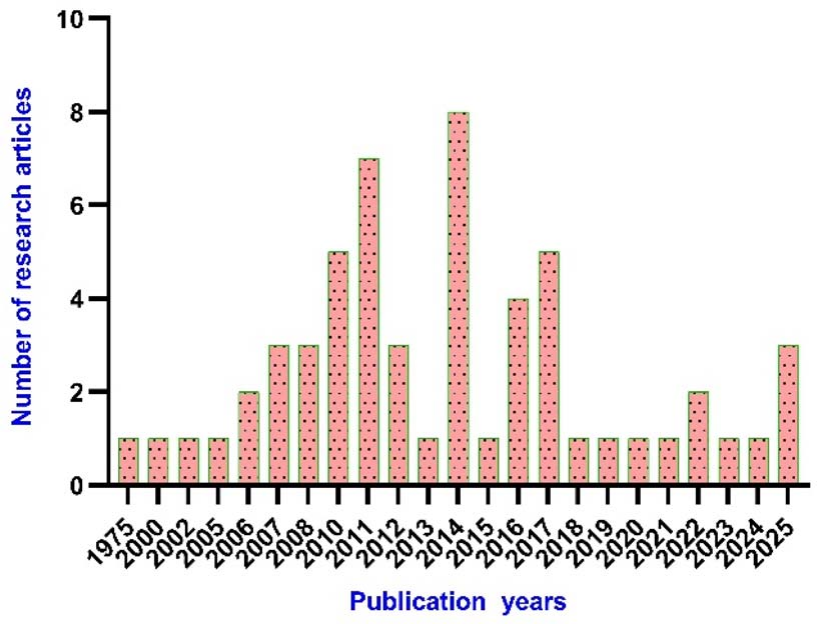
The number of research articles on pyrazolines in the field of antitubercular research, as per PRISMA.

**Fig. 8:**
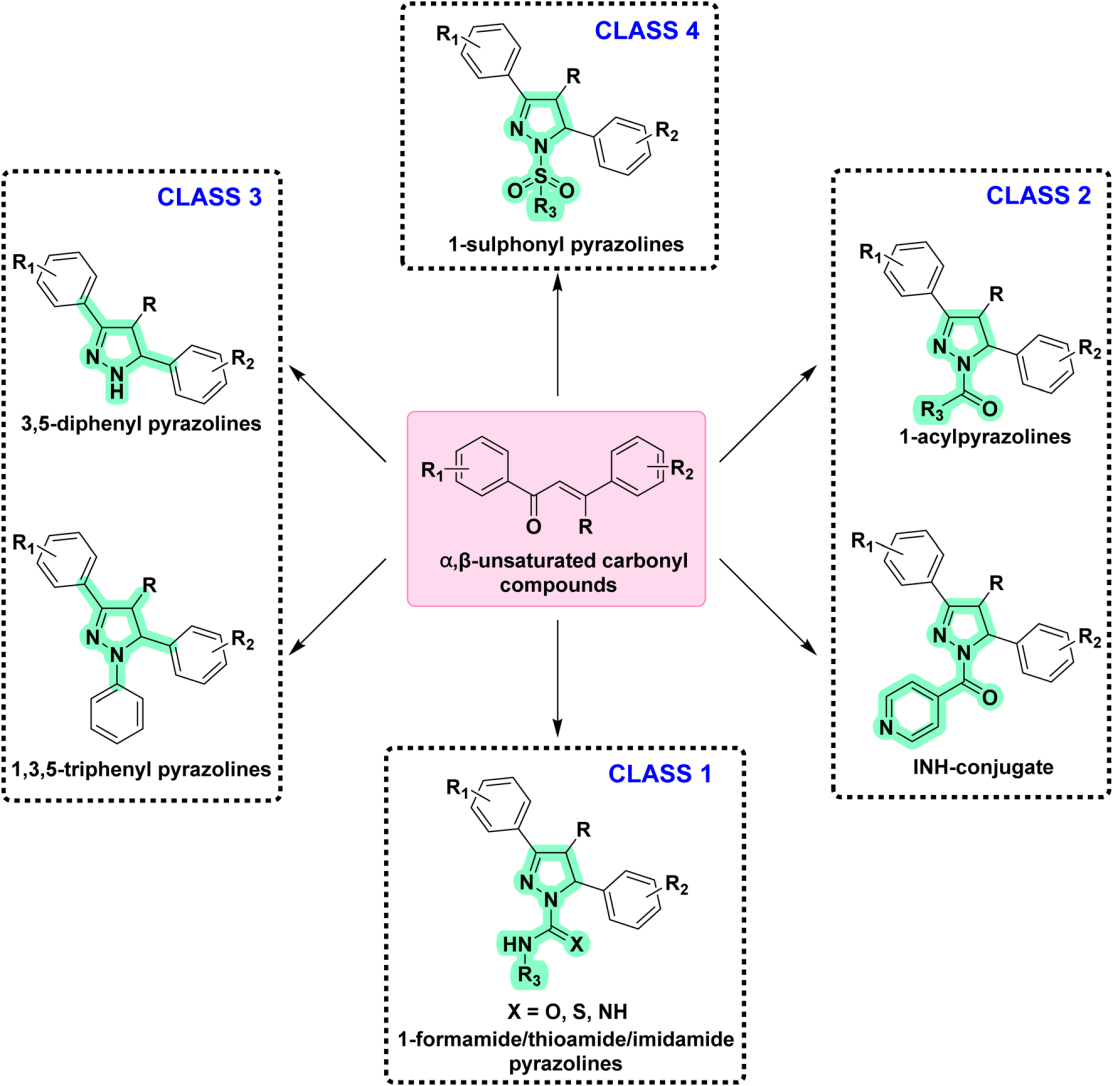
Organization of structural classes for the purpose of review and discussion. Cyan indicates the structural modifications in the primary pyrazoline scaffold.

After reviewing the available data on the antitubercular activities of pyrazolines, as shown in Fig. 7, we organised the information based on the chemical structure (Fig. 8) and within each class arranged chronologically. It is observed that (a) the number of compounds reported in each manuscript is minimal and rarely a well-conceived structure–activity relationship (SAR) account was presented by the author, (b) compounds from different classes (class 1–4, Fig. 8) were also reported in a manuscript, (c) evaluation protocol (antimycobacterial screening protocol) and activity reporting (% inhibition, IC_50_, MIC_50_, MIC_90,_*etc.*) were also widely varied from one scientific group to the other as well as within the group over a period of time, and (d) no target orientation was presented by the authors in general except very few.

Based on the above facts, the authors of this review did not attempt to present an overall SAR. Rather, they tried to provide information on the extent to which the chemical space around each class (Fig. 5) was explored to date with reference to antitubercular activity. This information will be extremely critical for any medicinal chemist to understand the existing gap and work upon it. Accordingly, in the following sections, we presented a class-wise discussion.

#### 1.3.1. Class 1

##### 1.3.1.1 Thiosemicarbazone-derived pyrazolines

The first thiosemicarbazone, thiacetazone (TBI/698), was reported for its anti-tubercular activity in the year 1946 by Domagk and his group.^64^ Since then, thiosemicarbazones have been explored for their antitubercular activity.^65–68^ Leveraging the antitubercular potential of open-chain thiosemicarbazones, Arthur and his group (1975) synthesized twelve novel 1,3,4 tri-substituted pyrazoline-5-ones *via* cyclizing thiosemicarbazone derivatives.^69^ They found the activity was retained even after cyclization, and the nature of substitution over the pyrazoline ring was found to influence the potency. The MICs ranged from 0.05 to 100 μg mL^−1^, with the most potent compound (1) exhibiting an MIC of 0.05–0.1 μg mL^−1^. Activity decreased significantly when compound (1) was converted to a free acid (1a) or a carboxamide (1b). Substituting the methyl thiocarbamoyl group with a thiocarbamoyl group or replacing the 3-methyl group with hydrogen also reduced activity (MIC = 0.5 μg mL^−1^). Extending the alkanoic acid chain did not increase potency (MIC = 1 μg mL^−1^). The presence of the methyl thiocarbamoyl group and the alkanoic acid group was crucial for maintaining high activity. These findings led to the subsequent exploration of the pyrazoline scaffold for antitubercular activity after a long gap of almost 30 years.

In 2007, Ali *et al.*, reported a series of twenty-two compounds by condensing pyrazolines with 2-methyl/methoxy phenyl isothiocyanates. During preliminary screening at 6.25 μg mL^−1^, four compounds showed inhibition in the range of 88–98%. Subsequent screening identified the compound with 2,6-dichloro phenyl substitution at the 5^th^ position of pyrazoline (2) as the most potent one amongst twenty-two, with a MIC value of 1.66 μg mL^−1^.^70^ In the following year (2008), they reported another twenty-two pyrazolines derived from phenyl isothiocyanate and 2-chloro phenyl isothiocyanate. This time, antitubercular evaluation was performed on INH-resistant *Mtb*. Once again, the compound with 2,6-dichloro phenyl substitution at the 5th position of pyrazoline (3) was found to be potent, with a MIC value of 0.96 μg mL^−1^, surpassing INH (1.86 μg mL^−1^) with a two-fold enhancement in activity.^71^

**Fig. 9:**
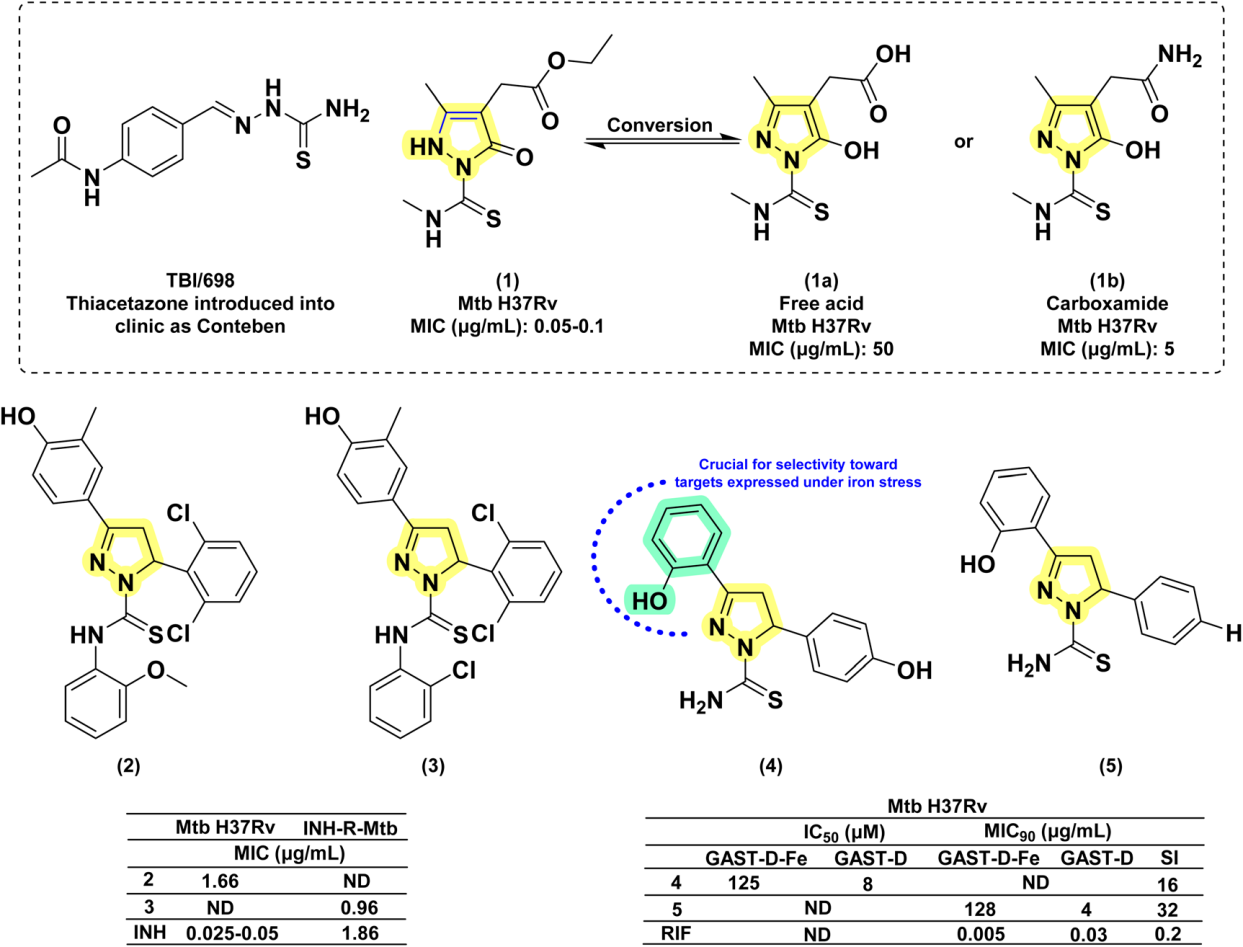
Chemical structures and anti-tubercular profiles of thiosemicarbazone-based analogues. Yellow highlights the pyrazoline ring system, while cyan indicates the key aryl features required for retention of activity (ND: not determined).

In the same year (2008), Stirrett *et al.* synthesized a library of thirty-two small-molecule inhibitors resembling the hydroxyphenyl-oxazoline portion of mycobactin, a mycobacterial siderophore. The idea was to interfere with the biosynthesis of mycobactin in mycobacteria, leading to iron deprivation. In order to identify the compounds interfering with the conditionally essential proteins expressed under iron stress, the experiment was conducted in an iron-rich and iron-deprived medium. 2-Hydroxyphenyl substitution at the third position of pyrazoline was retained, while the variation was shown in the other two phenyl rings at the first and fifth positions of pyrazoline.^72^ It was found that 2-hydroxyphenyl substitution at either the third or fifth position is crucial for selectivity towards conditionally essential targets expressed under iron stress. Amongst the thirty-two compounds, the one having 4-hydroxy phenyl substitution at the fifth position of the pyrazoline displayed enhanced potency and selectivity when there was no phenyl ring in the side chain at the first position of the pyrazoline (4). Accordingly, fourteen more compounds were reported by Mousumi *et al.* in 2022, showing variation in the phenyl ring at the fifth position of the pyrazoline ring.^73^ Surprisingly, the compound with an unsubstituted phenyl ring at the fifth position was found to be potent and selective against *Mtb* (5). In the case of compounds having 2-hydroxy phenyl substitutions at the fifth position of the pyrazoline ring, the potency and selectivity were influenced by the presence of a phenyl ring at the first position of the pyrazoline.^72,74^ Nevertheless, further investigative research is needed to optimise this scaffold (Fig. 9).

**Fig. 10:**
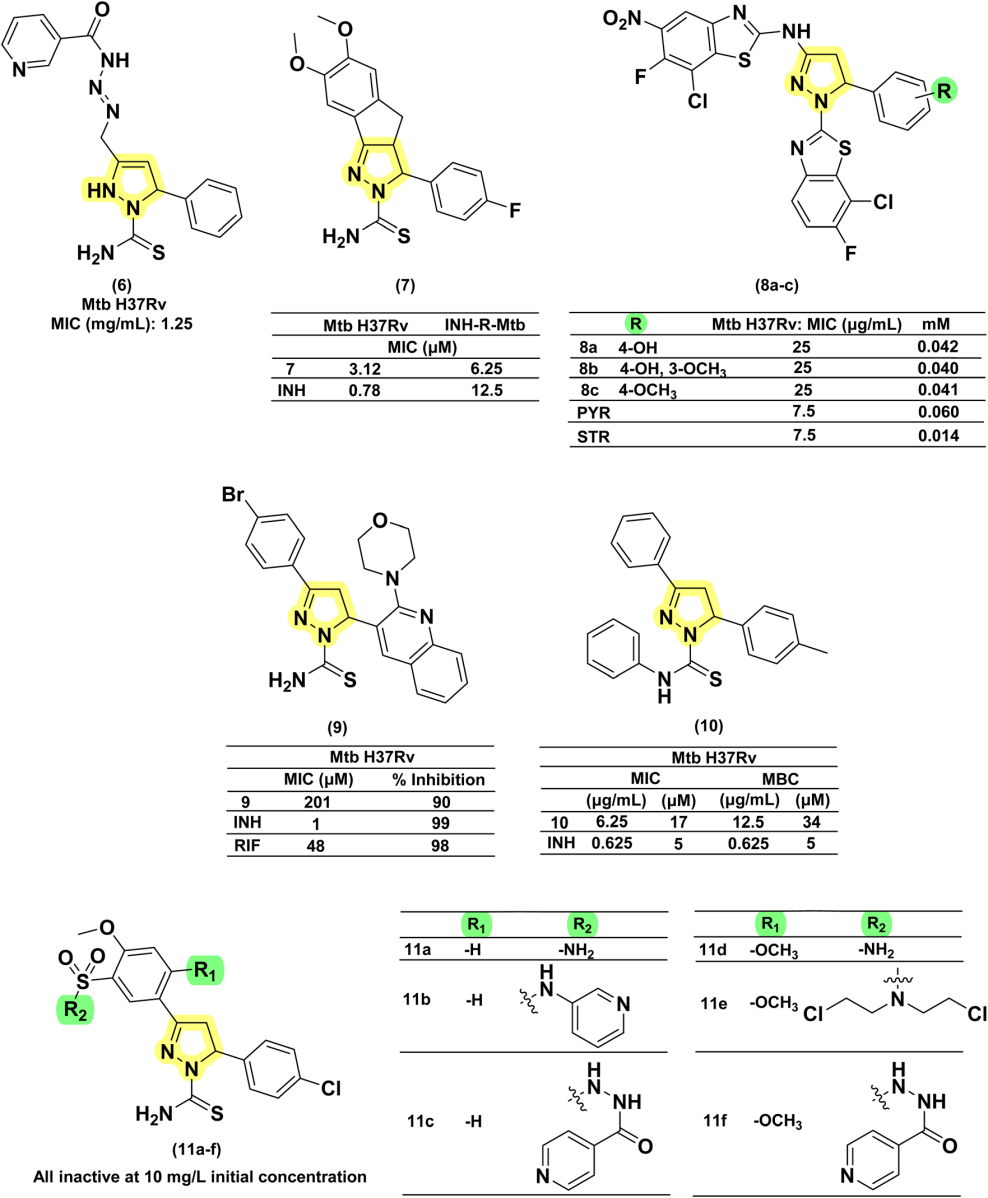
Chemical structures and anti-tubercular profiles of thiosemicarbazone-based analogues. Yellow highlights the pyrazoline ring system, while green indicates the alkyl/aryl substitution.

In 2010, Kasabe and Kasabe reported five 3-pyrazoline derivatives having β-picolinoyl amino azo methyl substitution at the third position of the pyrazoline ring. Variations on the phenyl ring at the fifth position of pyrazoline provided compounds with antitubercular and analgesic activity. Compound with an unsubstituted phenyl ring (6) was found to be the most potent amongst the five, with a MIC of 1.25 mg mL^−1^.^75^ Ali *et al.* in 2011 reported a series of fourteen 2-acyl-2,3-dihydro-1*H*-inden-1-one derivatives. The 4-fluorophenyl derivatives were found to be potent and equally effective in both wild-type and INH-resistant *Mtb* strains.^76^ The same group led by Ahsan *et al.* in 2011, visualized this scaffold as equivalent to chalcone in cyclizing thiosemicarbazide derivatives to indeno pyrazolines. This also provided a rigid tricyclic system by freezing the phenyl ring at the third position through a methyl bridge connecting the *ortho*-carbon of the phenyl ring with the 4^th^ carbon of the pyrazoline ring. They reported six rigid tricyclic indeno pyrazolines and evaluated them for antitubercular activity against both wild-type and INH-resistant *Mtb* strains. The 4-fluorophenyl derivative (7) was 4-fold less potent than INH in wild-type strains, whereas it was found to be more effective in INH-resistant strains by two-fold.^77^ In 2012, Hazra and co-workers reported a series of fluoro and nitro-substituted benzothiazolepyrazolines comprising eighteen compounds. The benzothiazole ring at the first position of the pyrazoline ring is a cyclic analogue of thiocarboxamide. The variations were shown in the substitution pattern on the heterocyclic (2-amino benzothiazole) and phenyl rings at the first, third, and fifth positions of pyrazoline, respectively. The compounds having hydroxy (8a–c) and methoxy (8c) functional groups at the third and fourth positions of the phenyl ring exhibited equal potency when the nitro group was present in the fifth position of the benzthiazole ring. These compounds were found to be better than the standard drug pyrazinamide (PYR).^78^ In 2016, Karad *et al.* reported a series of four compounds having a morpholine-substituted quinoline ring at the first position of the pyrazoline ring. They explored the variations in the phenyl ring at the third position of the pyrazoline. The compound (9), having a 4-bromo substitution, was found to be potent.^79^ Seven more compounds of similar nature were reported by Wong *et al.* in 2021 by condensing chalcone with phenyl-thiosemicarbazides. Variations were shown in the phenyl ring at the fifth position of pyrazoline, while the other two phenyl rings (first and third) were left unsubstituted. Only the compound (10) with 4-methyl phenyl substitution was found to be active with a MIC value of 17 μM.^80^ Castaño *et al.*, in 2022, reported six pyrazoline analogues bearing a sulphonamide group at the third position on the phenyl ring attached to the third position of the pyrazoline ring (11a–f). Upon evaluation against *Mtb* H37Rv at a concentration of 10 mg L^−1^, none of the compounds exhibited any growth inhibitory activity^81^ (Fig. 10).

##### 1.3.1.2 Semicarbazones

**Fig. 11:**
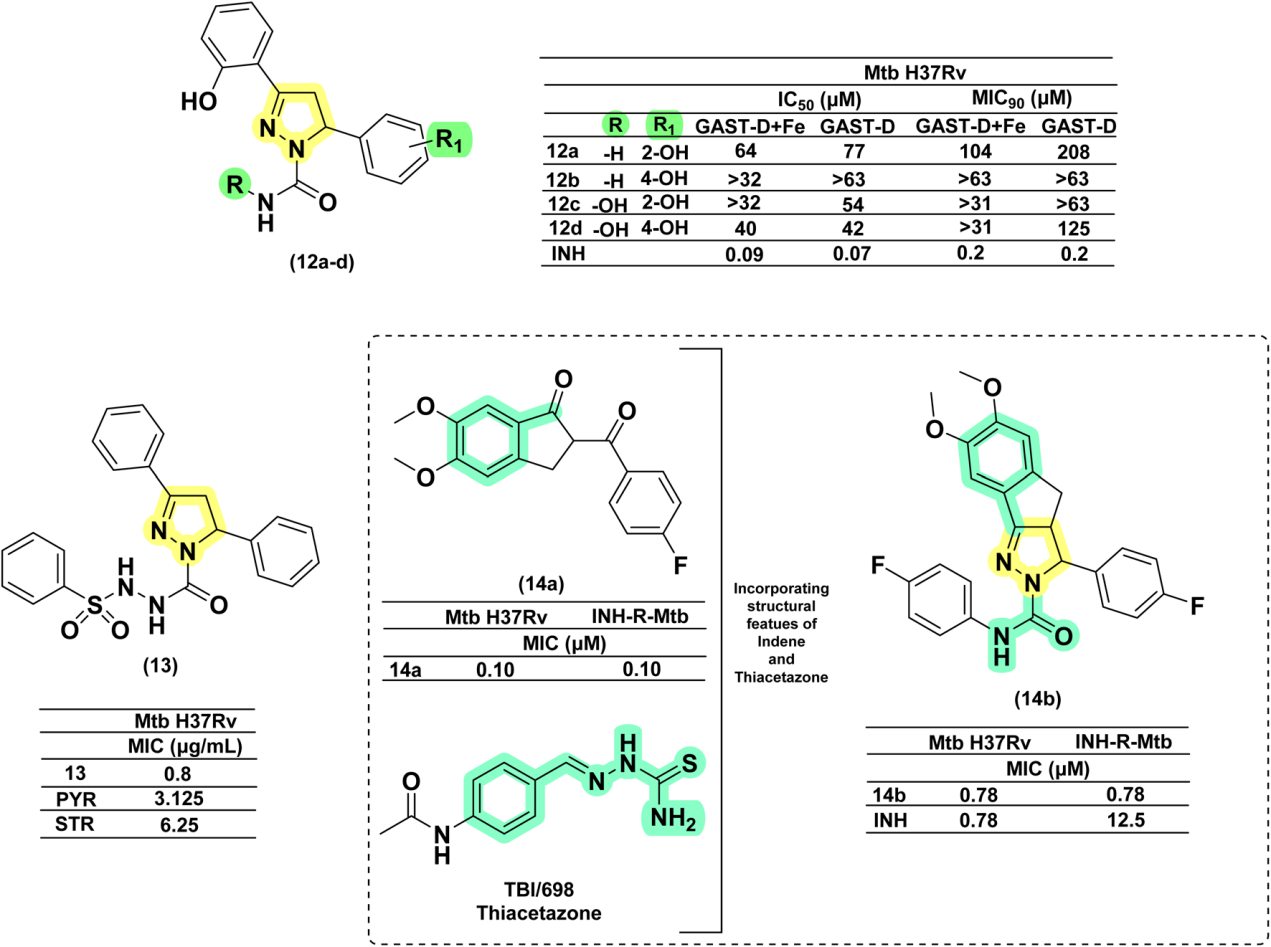
Chemical structures and anti-tubercular profiles of thiosemicarbazone-based analogues. Yellow highlights the pyrazoline ring system, while cyan indicates the structural features incorporated in the design strategy.

Condensation of chalcones with semicarbazide provides pyrazoline-1-carboxamide, a bivalent bioisosteric replacement of C=S with C=O. For the first time, Ferreras *et al.* reported two compounds (12a–b) as active analogues of the potent compound (4) reported by Stirrett *et al.*,^72^ mimicking the 2-hydroxy phenyloxazoline portion of mycobactin.^74^ Corresponding *N*-hydroxy analogues (12c–d) were also tested, and none of them were found to be better than their thiosemicarbazide counterparts.^74^ Sadashiva *et al.*, in 2017, reported a series of fourteen pyrazolines carboxamide derivatives with extended benzene sulphonamides. The authors attempted to generate a hybrid of two well-known antibacterial pharmacophoric features: pyrazolines and sulphonamides. Interestingly, the compound having no substitution (13) on all three phenyl rings was found to be the most potent one. It was also found to be four-fold more potent than pyrazinamide and around eight-fold more potent than streptomycin (STR). Substitution on the phenyl ring at the fifth position of the pyrazoline with 4-chloro and 4-bromo/4-methyl mercapto groups led to a two- and four-fold decrease in activity, respectively, when compared with compound 13.^82^ Encouraged by the antitubercular activity of indeno pyrazoline (7), Ahsan *et al.* in 2011 went on to report the activity of forty-two indeno pyrazoline derivatives prepared from semicarbazide with variations in the phenyl rings at the second and third positions of the indeno pyrazoline ring. All the compounds were evaluated for their antitubercular activity against both wild-type and INH-resistant *Mtb* strains. The compound having 4-fluorophenyl substitution on both the second and third positions of the indeno pyrazoline ring (14b) was found to be equipotent to INH (0.78 μM) in wild-type strain, whereas it was found to be effective in INH-resistant strain by sixteen-fold. Cyclization results in retention of activity, but the potency was decreased to – eightfold in comparison with 14a. Another six derivatives were reported by the removal of the phenyl ring at the second position. However, this failed to generate any potent compounds^83–86^ (Fig. 11).

#### 1.3.2. Class 2

##### 1.3.2.1 Acetyl pyrazolines

**Fig. 12:**
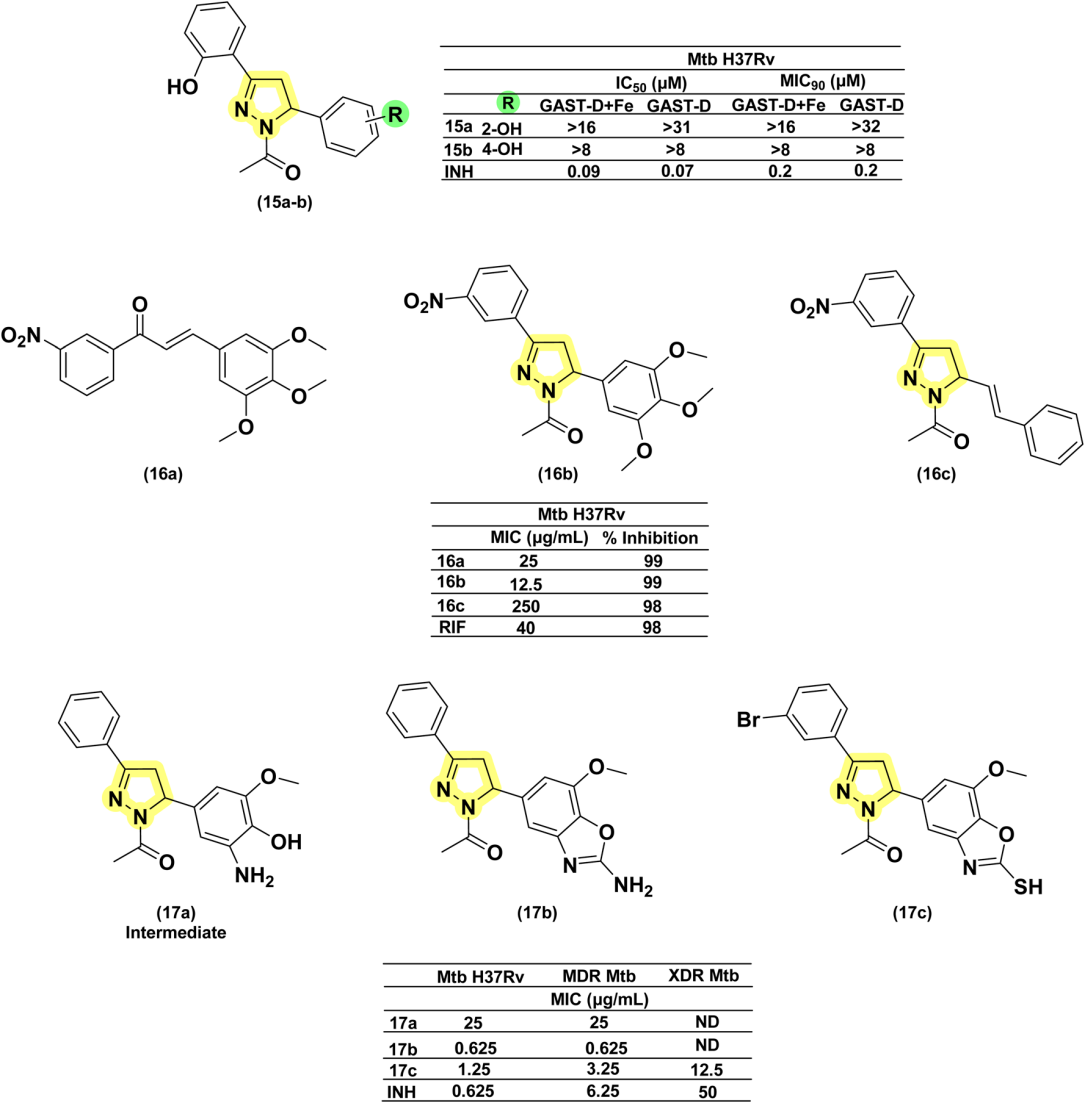
Chemical structures and anti-tubercular profiles of acetyl pyrazolines-based analogues. Yellow highlights the pyrazoline ring system, while green indicates the alkyl/aryl substitution (ND: not determined).

Condensation of chalcones with hydrazine in acetic acid as solvent provides 1-acetyl pyrazolines, a bivalent bioisosteric replacement of C=S with C=O, and a monovalent Grimm's hydride replacement of –NH_2_ with –CH_3_. For the first time, Ferreras *et al.* 2011 reported two compounds (15a–b) as active analogues of the potent compound (6) reported by Stirrett *et al.*,^72^ mimicking the 2-hydroxy phenyl oxazoline portion of mycobactin.^74^ This modification failed to generate active compounds.^72,74^ Further, Mousumi *et al.* in 2022 attempted different substitutions (including the two analogues (15a–b) reported by Ferreras *et al.*^74^) over the phenyl ring at the 5th position of the pyrazoline with different substitutions at *ortho*, *meta*, and *para* positions (–OH, –OCH_3_, –Cl, –Br, –F). This attempt also failed to generate active compounds.^73^ Monga *et al.* 2014, reported the antitubercular activity for seven acetyl pyrazolines and corresponding nitro-chalcones. The tri-methoxy phenyl derivative of both pyrazoline (16b) and their corresponding chalcone (16a) was found to be three and two-fold more potent than rifampicin, respectively. It is interesting to know that the cyclization of chalcone to pyrazoline results in the enhancement of potency. It is also observed that the replacement of phenyl with styryl at the 5^th^ position of pyrazoline (16c) resulted in the enhancement of activity. However, substitutions over the styryl ring were not explored.^87^ In 2013, Rana *et al.* employed a hybrid pharmacophore approach to investigate the antituberculosis potential of the pyrazoline–benzoxazole hybrid.^58,88–91^ The authors evaluated eight intermediate (-3-amino-4-hydroxy phenyl substitution at 5^th^ position of pyrazoline ring) pyrazolines along with thirty-seven pyrazoline–benzoxazole hybrids (2-amino/mercapto benzoxazole-5-yl at 5^th^ position of pyrazoline ring) for antitubercular activity against wild type, MDR, and XDR strains. The intermediate with unsubstituted phenyl and 5-methoxy phenyl substitution at the third and fifth positions of the pyrazoline ring (17a) was found to have poor activity against both strains. Cyclization with cyanogen bromide/carbon disulfide resulted in the formation of amino/mercapto benzoxazole hybrid compounds, respectively. Cyclization improved the potency of the final compounds (17b–c) in comparison to their corresponding intermediate (17a). The amino counterpart (17b) was found to be equipotent to the standard drug INH over both strains under study. Whereas, the mercapto analogue (17c) displayed effectiveness against both MDR and XDR strains^91,92^ (Fig. 12).

**Fig. 13:**
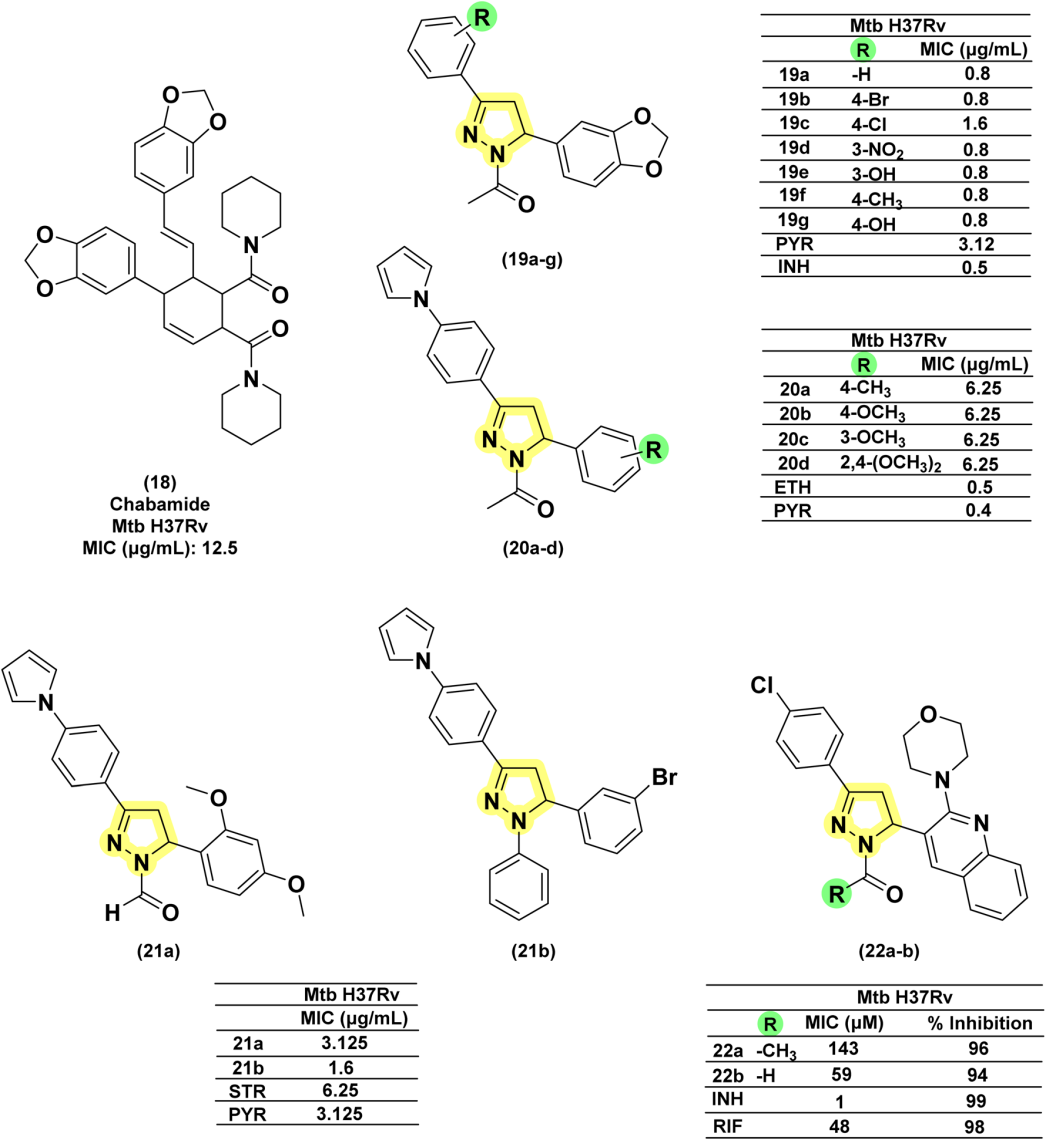
Chemical structures and anti-tubercular profiles of acetyl pyrazolines-based analogues. Yellow highlights the pyrazoline ring system, while green indicates the alkyl/aryl substitution.

In 2014, Deshpande *et al.* designed a series of seven acetyl pyrazoline analogues from chalcones derived with piperonal, considering the antitubercular activity of chabamide (18).^93^ All the analogues (19a–g) except one (having 4-chloro substitution) were found to have comparable activity to that of INH while displaying a better activity profile (four-fold) than pyrazinamide.^93^ Joshi *et al.* in 2016 reported a series of seventeen pyrazoline derivatives bearing the pharmacophoric features of InhA inhibitors, 4(pyrrol-1-yl)phenyl. Variations were shown in the phenyl ring at the fifth position of the pyrazoline ring with electron-pumping and electron-withdrawing groups, especially methoxy groups (20a–d). All compounds were evaluated for anti-TB activity against *Mtb* H37Rv. Four compounds (20a–d) possessing electron-pumping groups, methyl, and methoxy, exhibited an MIC of 6.25 μg mL^−1^.^94^ Replacing the acetyl group with formyl (21a) did not provide any significant enhancement, whereas with phenyl (21b), a significant enhancement was observed.^95,96^ Similarly, pyrazoline–sulphonamide conjugates reported by Castaño *et al.* with acetyl and formyl substitution in the first position failed to show any inhibitory potential at 10 mg per L concentration.^97^ In 2016, Karad *et al.* reported a series of four acetyl pyrazolines having a morpholin-2-yl quinoline at the fifth position. They explored the variations in the phenyl at the third position of the pyrazoline, and the compound with 4-chloro substitution (22a) was found to be potent. In this case, also, replacing the acetyl group with formyl provided compounds (22b) with equipotency except for the 4-fluoro derivative^98^ (Fig. 13).

##### 1.3.2.2 Acyl pyrazolines

**Fig. 14:**
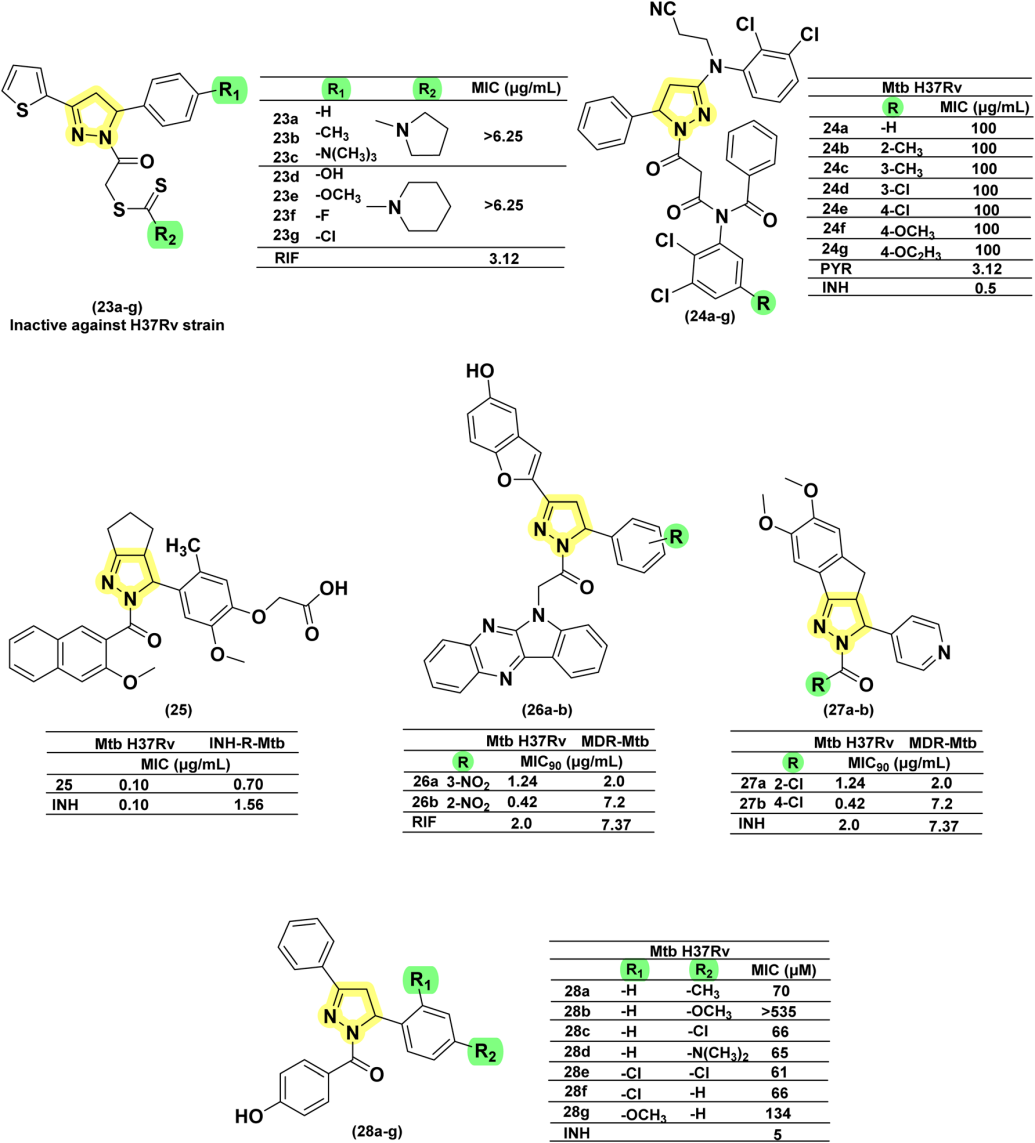
Chemical structures and anti-tubercular profiles of acyl pyrazolines-based analogues. Yellow highlights the pyrazoline ring system, while green indicates the alkyl/aryl substitution.

Inspired by the antimicrobial activities reported in literature studies, Zitouni *et al.*, in 2005, reported a series of pyrazoline derivatives incorporating *N*,*N*-disubstituted dithiocarbamic acid. However, upon testing these derivatives (23a–g) against the *Mtb* H37Rv strain, they were found to be inactive, leading to the discontinuation of research in this direction.^99,100^ Sharma *et al.* in 2010 reported a series of 3-amino-5-phenyl pyrazoline derivatives (24a–g) and evaluated them against the *Mtb* H37Rv strain. All the analogues were found to have tuberculostatic activity at 100 μg per mL concentration.^101^ Shahar Yar *et al.*, in 2006, reported acyl-pyrazolines with phenyl/benzyl and fused cyclopentyl in the fifth and between the fourth and fifth position of pyrazoline, respectively, from chalcones prepared with 2-(4-formyl-2-methoxyphenoxy) acetic acid. Compound (25), having phenoxy acetyl substitution in the first position, was found to be potent and comparable to INH against *Mtb* H37Rv, while it was approximately two-fold more potent compared to INH-resistant strains.^102^ Manna *et al.*, in 2009, reported antibacterial activity for indophenazine–pyrazoline conjugates having benzofuran and substituted phenyl ring/furan rings in the third and fifth positions, respectively. Encouraged by the broad-spectrum antibacterial activity, they synthesized and evaluated 5-hydroxybenzofuran-2-yl analogues of the earlier reported series against *Mtb* H37Rv and MDR-*Mtb*. The *meta* and *ortho*-nitro derivatives (26a–b) were found to show the best antitubercular activity against both the strains (wild and resistant) studied.^59,103^ In line with their previous report, Ali and the group extended their work by designing twenty-nine indeno-pyrazolines derived from acyl hydrazides. The variations were explored in the phenyl ring at the first and fifth positions of the pyrazoline ring. Two compounds (27a–b) featuring chloro substitution at the *meta* and *para* positions demonstrated significant efficacy against both wild-type *Mtb* H37Rv and resistant strains. Their activity against the wild strain was comparable to INH, whereas the potency was four times higher against the resistant strain.^104,105^ Wong *et al.*, in 2021, designed seven compounds by condensing chalcone with *para*-hydroxybenzoyl hydrazide. Variations (28a–g) were shown in the *ortho* and *para* positions of the phenyl ring at the fifth position of pyrazoline, while the phenyl ring at the third position was left unsubstituted. The MIC range was observed between 61–134 μM, and none of them was found to be better than INH^80^ (Fig. 14).

##### 1.3.2.3 Isoniazid conjugates

**Fig. 15:**
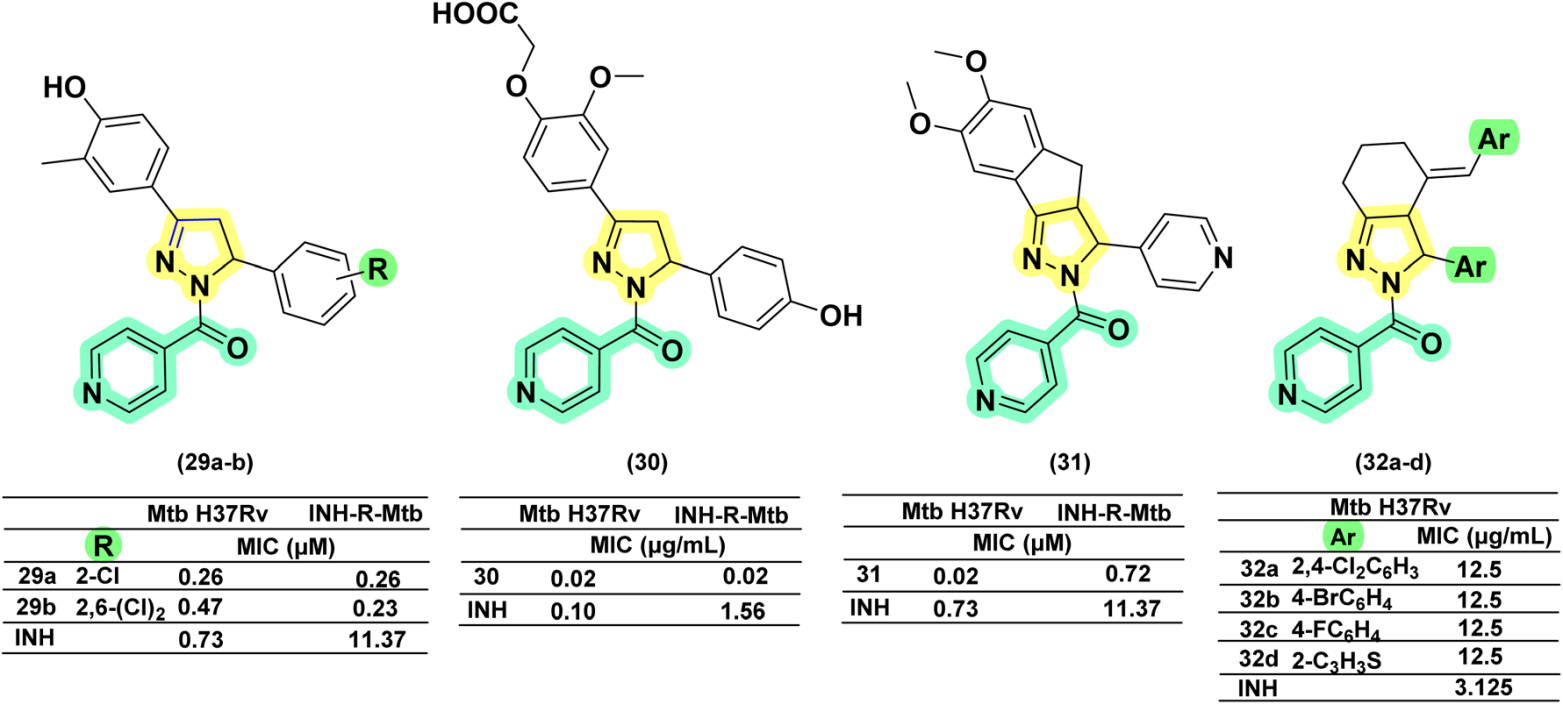
Chemical structures and anti-tubercular profiles of isoniazid drug conjugates. Yellow highlights the pyrazoline ring system, green indicates the alkyl/aryl substitutions, and cyan highlights the part of the isoniazid ring system.

Isoniazid (INH), a first-line anti-TB drug, remains effective despite its associated toxicity and the emergence of resistance. Medicinal chemists have consistently sought to improve INH by modifying its structure, with the hydrazide moiety offering significant opportunities for the development of various derivatives. Among these, the cyclization of the hydrazide segment into pyrazoline derivatives has garnered considerable attention, as it holds promise for addressing both resistance and toxicity concerns. The discussion relevant to these aspects has been presented below. In 2006, Shaharyar and his team reported the design, synthesis, and *in vitro* antimycobacterial evaluation of eleven derivatives against *Mtb* H37Rv and INH-resistant strains. Among the eleven derivatives, four exhibited superior potency compared to INH, with MICs ranging between 0.23 and 0.58 μM. Chlorine (Cl) substitution at the *ortho* position (29a) was found to enhance potency against resistant strains (0.26 μM). However, *ortho* di-substitution (29b) (0.23 μM) did not result in a significant improvement in activity compared to *ortho* mono-substitution.^106^ Further, this research group, led by Ali *et al.* in 2007, derivatized a 4-OH functional group on the phenyl ring at the 3rd position of the pyrazoline ring to oxyacetic acid. This derivatization failed to improve the activity of five analogues reported in their previous study. However, the compound (30) with 4-hydroxyphenyl substitution at the 5th position of pyrazoline exhibited potent inhibitory activity against both *Mtb* and INH-resistant *Mtb* at a concentration of 0.02 μg mL^−1^.^107^ Further, in an attempt to design a potent molecule, they went on to generate tricyclic compounds by freezing the phenyl ring at the position through a methyl bridge connecting the *ortho*-carbon of the phenyl ring with the 4^th^ carbon of the pyrazoline ring. The INH analogue (31) demonstrated potent activity (3.12 times and 15.7 times more potent than INH) against both *Mtb* and INH-resistant *Mtb*, showing approximately a 4-fold improvement when the third position of pyrazoline is occupied with the 4-pyridyl functional group.^108^ Replacement of pyridine-4-carboxyl in the first position of pyrazoline with substituted-benzoyl also resulted in compounds effective against resistant INH strains, but are found to be less potent than (31).^109^ Another group led by Napoleon *et al.* in 2015 described the development of a bicyclic pyrazoline derivative based on di-arylidene cyclohexanones. In this design, a phenyl group was connected to the pyrazoline ring at the 3rd position through a two-carbon unit with an extended exocyclic double bond. The modification resulted in ten analogues, of which four were active (32a–d) at a MIC of 12.5 μg mL^−1^ but 4-fold less potent when compared to INH (3.125 μg mL^−1^)^60^ (Fig. 15).

**Fig. 16:**
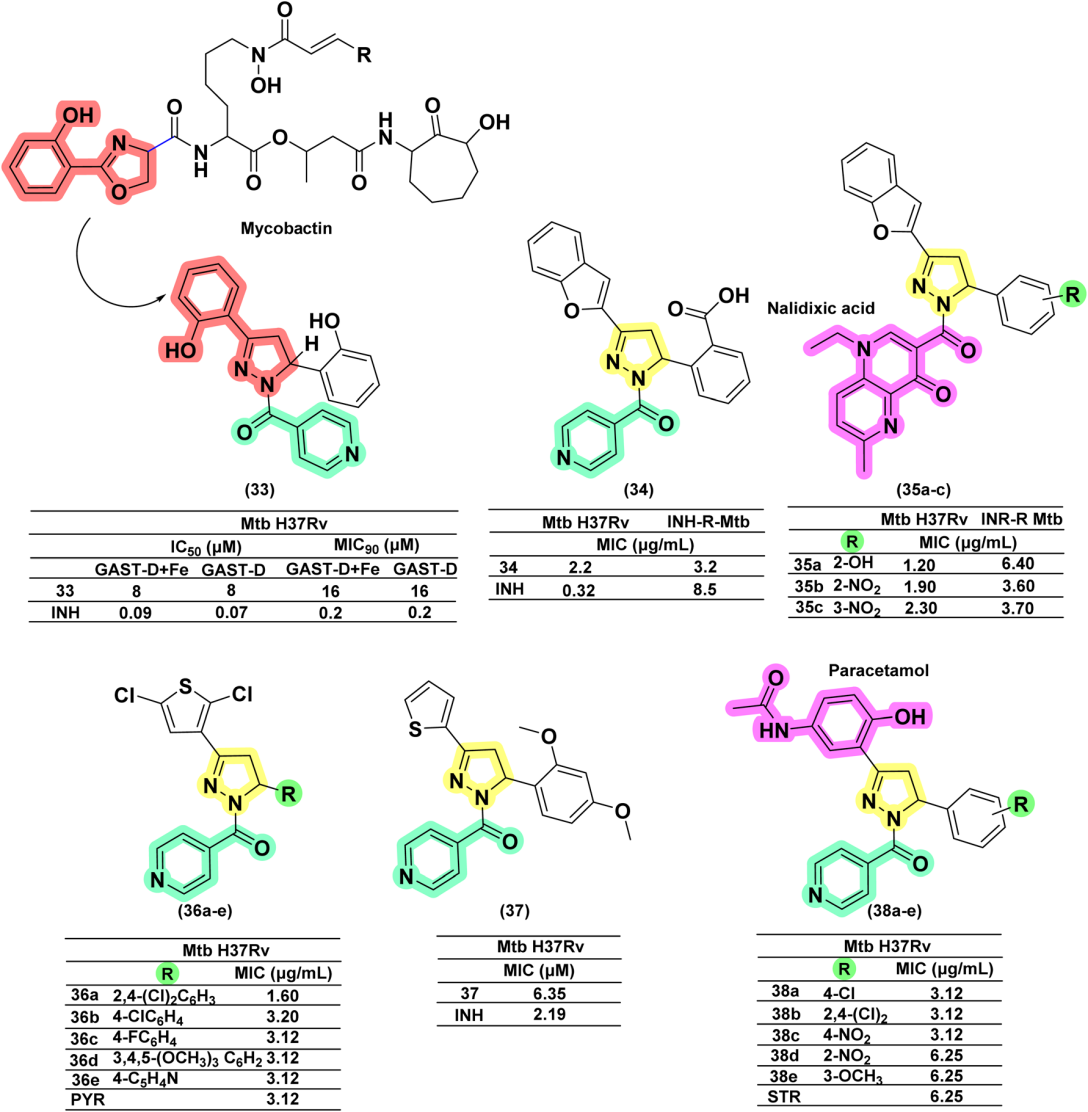
Chemical structures and anti-tubercular profiles of isoniazid drug conjugates. Yellow highlights the pyrazoline ring system, green indicates the alkyl/aryl substitutions, and cyan marks the portion of the isoniazid ring system. Pink highlights the drug moieties attached to the ring system, forming a conjugate, while red represents the features incorporated in the design strategy.

Ferreras *et al.* 2011 reported two novel derivatives designed to mimic the 2-hydroxyphenyloxazoline portion of mycobactin (iron chelators), aiming to disrupt the crucial iron acquisition pathway in mycobacteria. However, the compound with a 2-hydroxyphenyl substitution at the 5th position of pyrazoline (33) was found to have limited efficacy, as it did not interfere with iron metabolism. With a MIC of 8 μg mL^−1^, it was 90-fold less potent than INH.^74^ In 2010, Manna and Agrawal reported a series of fourteen derivatives with benzofuran and phenyl substitutions at the third and fifth positions of pyrazoline, respectively. The compound having *ortho*-COOH substitution on the phenyl ring (34) exhibited nearly ∼4-fold higher potency (MIC 2.2 μg mL^−1^) compared to the unsubstituted derivative (MIC 8.5 μg mL^−1^) against *Mtb* and ∼3-fold higher potency (MIC 3.2 μg mL^−1^ and 9.5 μg mL^−1^ [unsubstituted]) against MDR-resistant *Mtb*. The authors suggested that the acidity of the –COOH group plays a significant role in influencing the activity. However, irrespective of the presence of electron-donating or electron-withdrawing groups, no improvement in activity was observed against INH-resistant *Mtb*. A similar conjugate with nalidixic acid (35a–c) displayed a profile equivalent to INH conjugates.^110^ In 2019, Bontha Venkata and his team synthesized and characterized a series (36a–e) of twenty new pyrazolines derived from a novel 2,5-dichloro-3-acetylthiophene chalcones. Upon *in vitro* evaluation of antitubercular activity against *Mtb* H37Rv, compound (36a) possessing a 2, 4-dichlorophenyl group at the 5-position of the pyrazoline ring was found to be most potent (2-fold activity than PYR) with a MIC of 1.60 μg mL^−1^. Additionally, five other compounds in the series demonstrated similar activity to the standard drug pyrazinamide, with an MIC of 3.12 μg mL^−1^. Highly electronegative groups such as chloro (36b), fluoro (36c), and methoxy (36d) were found to boost antitubercular activity. Among nitrogen-based heterocycles, only the compound with a 4′′-pyridinyl group (36e) exhibited significant activity. These results indicate that electron-pumping substituents (F, Cl, OCH_3_) on the phenyl ring contribute to strong antitubercular properties.^111^ A series of ten novel 3-thiophene–pyrazolines were reported by Rasgania *et al.* in 2024, considering the structural features of INH Schiff-bases, pyrazolines, and thiophenes possessing antitubercular activity in their design. Compound (37), with a 2,4-dimethoxy phenyl substituent, demonstrated potent antitubercular activity against *Mtb* H37Rv, with a MIC of 6.35 μM, but 3-fold less potent than INH (MIC 2.19 μM).^112^ A research group led by Ahmad *et al.* in 2016, from King Saud University, reported INH-derived pyrazoline conjugated with paracetamol at the third position and evaluated them as antimicrobial and antimycobacterial agents. Three compounds (38a–c) were found to be more potent than streptomycin; however, the authors did not compare the activity with INH. Whereas, two compounds (38d–e) having 1-*N*-phenyl substitution were found to have potency equivalent to streptomycin^113^ (Fig. 16).

**Fig. 17:**
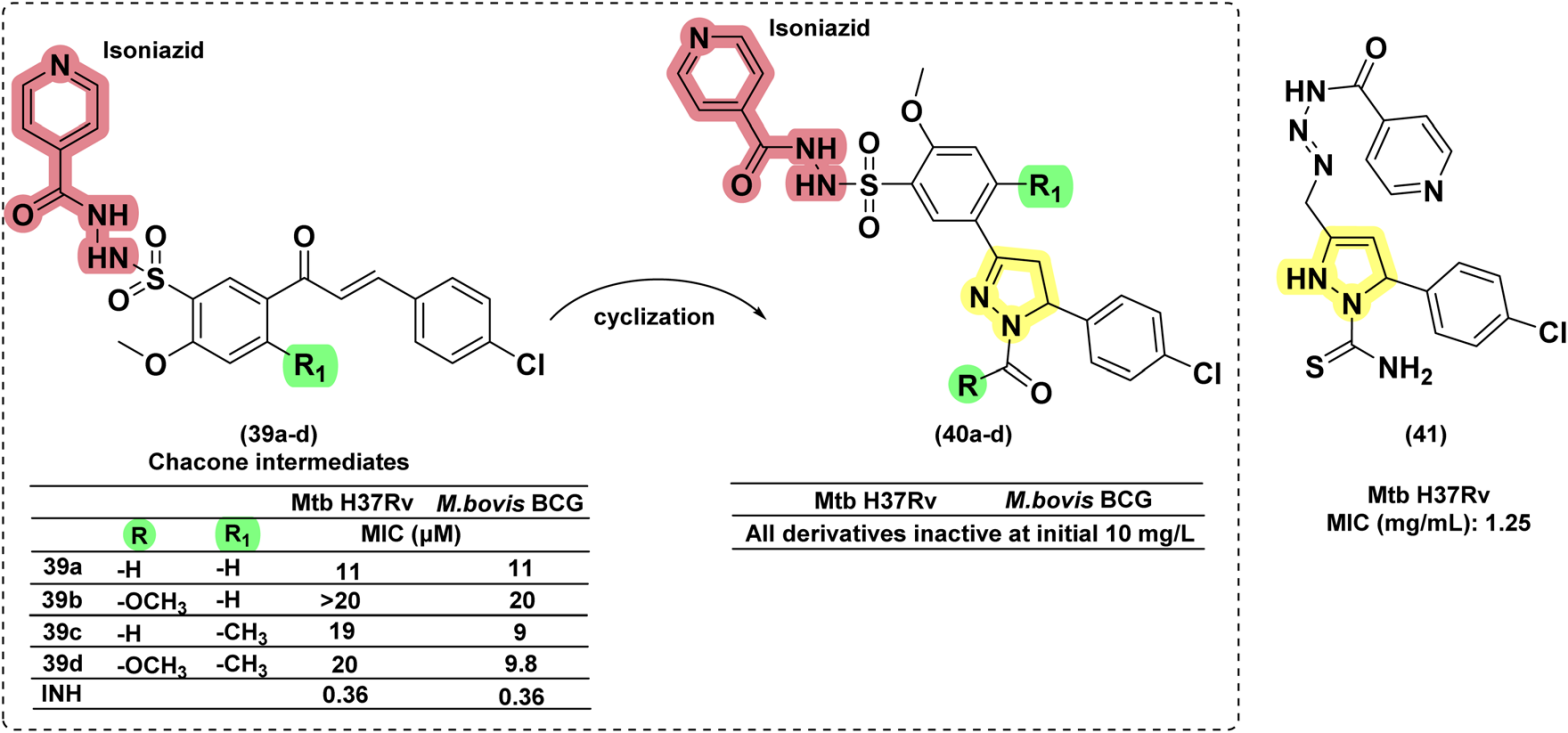
Chemical structures and anti-tubercular profiles of isoniazid drug conjugates. Yellow highlights the pyrazoline ring system, and green indicates the alkyl/aryl substitutions. Red highlights the drug moiety attached to the ring system, forming a conjugate (features incorporated in the design strategy).

A few research groups also reported INH conjugated with pyrazolines as a side chain. In 2022, Castaño *et al.* reported six pyrazoline hybrid analogues in which INH is linked to the *meta*-position of the phenyl ring at the third position of pyrazoline through the –SO_2_– bridge. None of the compounds (40a–d) in the series was found to be active against *M. bovis* BCG and *Mtb*. The other twelve sulphonamide derivatives were also found to be inactive. Interestingly, their chalcone counterparts (39a–d) were found to be active but not potent enough in comparison with INH (27-fold less potent).^114^ Kasabe and Kasabe, in 2010, reported nicotinyl hydrazide (a positional isomer of INH) conjugated with pyrazoline at the third position through an azo-linker. Compound (41) was found to be less potent, showing activity at 1.25–6.25 mg per mL concentration^115^ (Fig. 17).

##### 1.3.2.4 Other drug conjugates

**Fig. 18:**
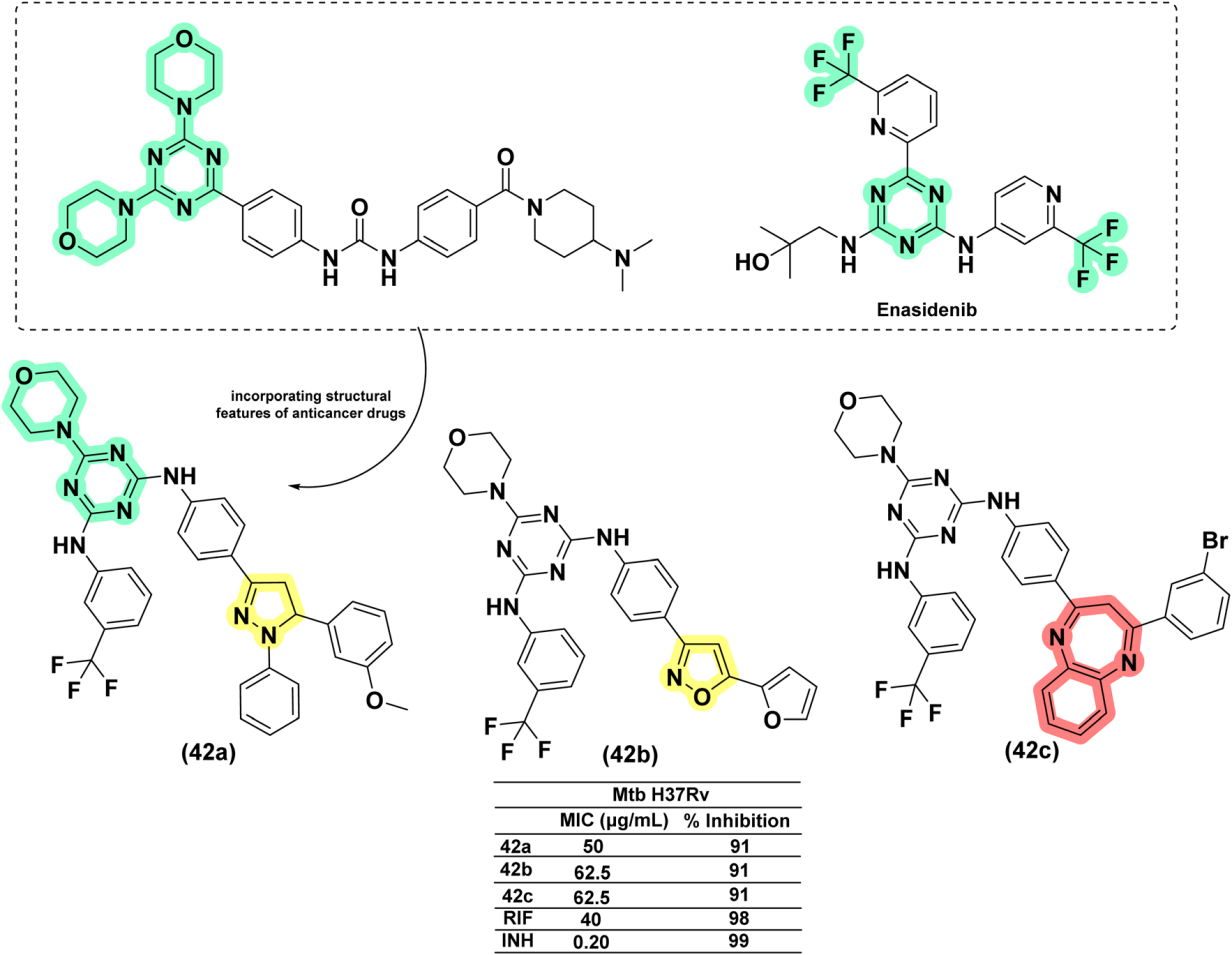
Chemical structures and anti-tubercular profiles of isoniazid drug conjugates employing design strategies. Yellow highlights the pyrazoline ring system, and cyan marks the features incorporated in the design strategy, while red marks the benzodiazepine moiety.

**Fig. 19:**
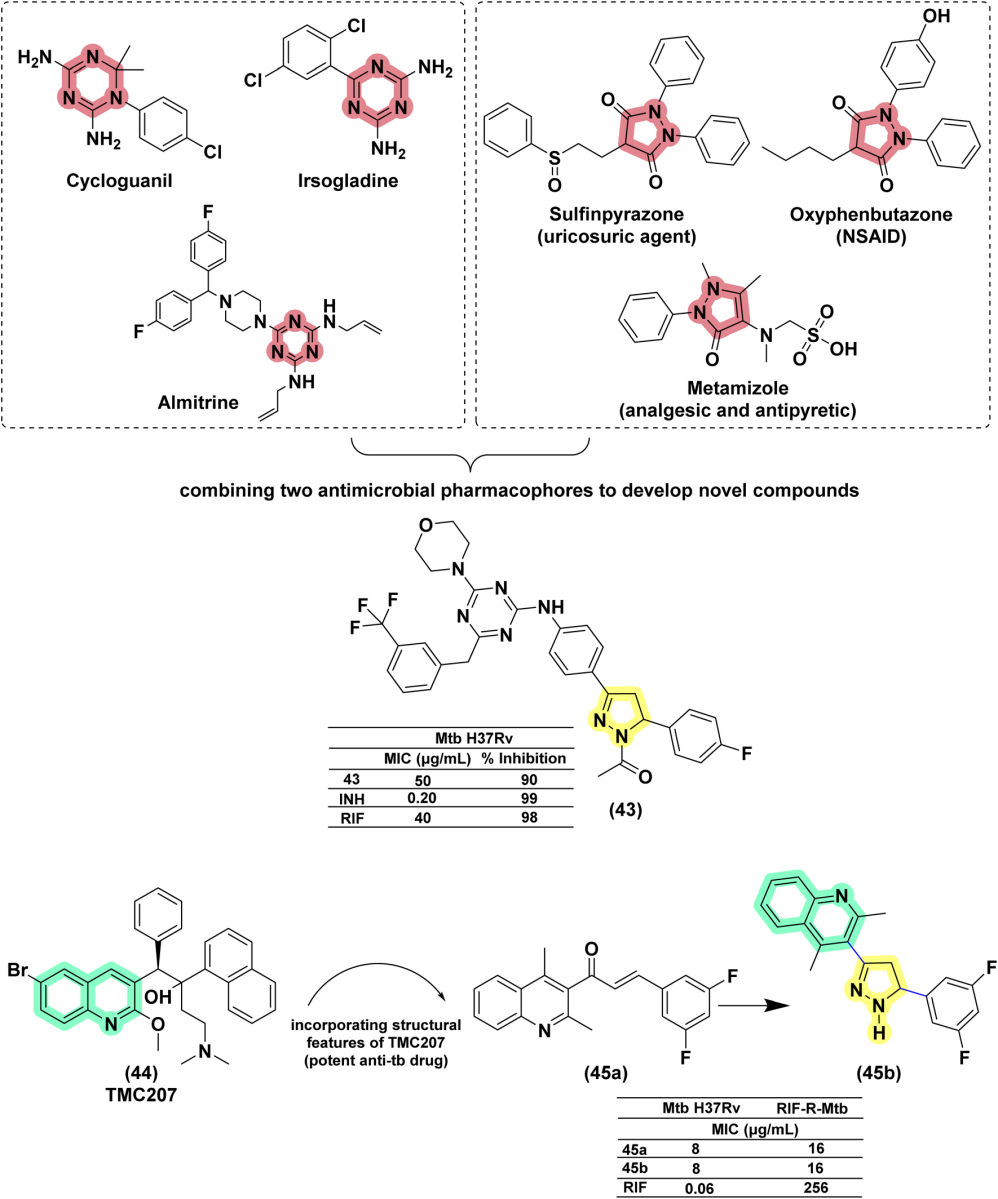
Chemical structures and anti-tubercular profiles of quinoline-linked pyrazoline analogues. Yellow highlights the pyrazoline ring system, while red represents the features incorporated in the design strategy.

Anjani and her co-workers (2016) designed and synthesized fifteen pyrazoline–triazine conjugates with structural features of anti-cancer drugs, *i.e.*, gedatolisib and enasidenib. They were evaluated for their antimicrobial and antimycobacterial activity. The pyrazoline conjugate (42a) was found to be better than its oxazoline (42b) and benzodiazepine counterparts (42c). The activity was found to be equivalent to rifampicin.^116^ Similar conjugates were reported recently by Tailor *et al.* (2025) compound 43, incorporating electron-withdrawing groups such as fluorine (–F) on the phenyl ring at the 5-position of the pyrazoline core, exhibited good antitubercular activity; however, its potency was lower compared to standard drugs like isoniazid and rifampicin.^117^ In 2017, Rao designed quinoline-linked chalcones and corresponding pyrazoline conjugates by incorporating the structural features of the potent anti-TB drug (TMC207: 44), which is in phase 2 clinical trials. Five compounds were found to be equipotent on *Mtb* H37Rv and its rifampicin-resistant strain at concentrations between 16 and 64 μg mL^−1^. The compound having a 3,5-difluoro phenyl substituent (45b) at the fifth position of the pyrazoline ring had a potency 16-fold higher against the rifampicin-resistant mycobacterial strain than the wild-type strain. Interestingly, their chalcone counterparts (45a) were found to be more active (4–8 μg mL^−1^) than corresponding pyrazolines and more potent (32 and 64-fold) than rifampicin against the rifampicin-resistant strain of *Mtb* in comparison with the wild-type strain.^118^ (Fig. 18 and 19).

#### 1.3.3. Class 3

##### 1.3.3.1 3,5-Diphenyl pyrazolines

**Fig. 20:**
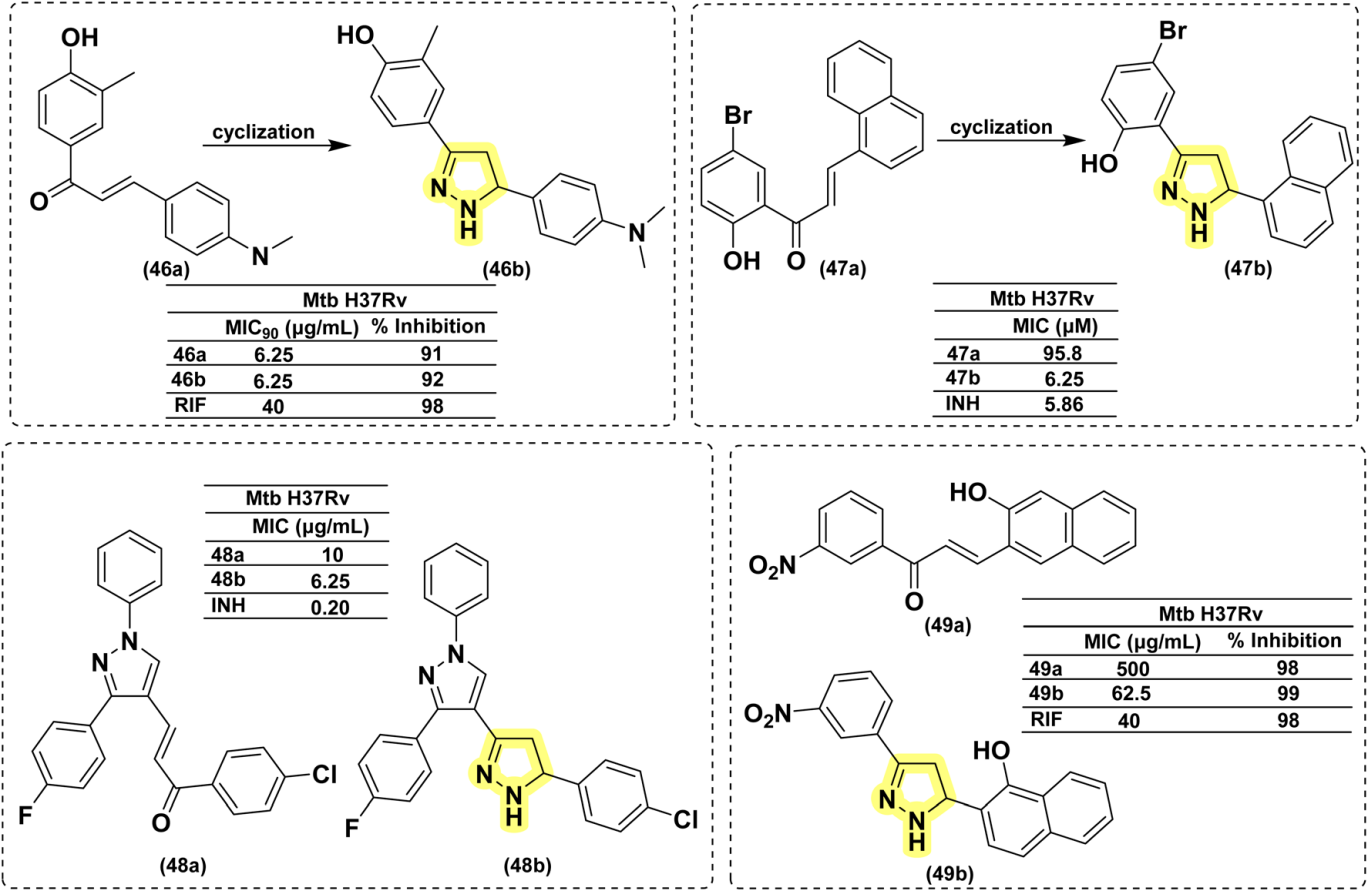
Chemical structures and anti-tubercular profiles of 3,5 di-substituted pyrazolines-based analogues. Yellow highlights the pyrazoline ring system.

The research group led by Yar in 2007^119^ reported eleven novel pyrazoline derivatives synthesized using their set protocol as mentioned in their early studies.^106^ All the compounds were evaluated for their antimycobacterial activity against *Mtb* H37Rv, and amongst all, only Compound (46b) exhibited a 92% inhibition at 6.25 μg mL^−1^. Interestingly, the corresponding chalcone (46a) also showed 91% inhibition at the same concentration. It clearly shows that the activity of chalcone was not affected by cyclization to pyrazoline. This provides an option for medicinal chemists to explore the chemical space around pyrazoline during the lead optimization process in the drug discovery pipeline. In a similar approach, Pola *et al.* reported antimycobacterial activity for a series of chalcones of naphthalene-1-carboxaldehydes and their corresponding pyrazolines. It was observed that cyclization to pyrazoline had improved the activity in general, with few exceptions where no significant change was observed. Potency improvement to the tune of ∼15-fold was observed for compound (47b) in comparison with its corresponding chalcone (47a) carrying a 5-bromo-2-hydroxyphenyl substitution at the third position and an unsubstituted naphthyl group at the fifth position of pyrazoline.^120^ Shelke *et al.* 2012 reported a similar comparison of heterocyclic chalcones and their corresponding pyrazolines.^121^ No significant improvement in activity was found due to the cyclization of chalcone (48a) to pyrazoline (48b). In 2017, Rao *et al.* compared the activity of quinoline-linked chalcone (45a) with their corresponding pyrazolines (45b). They also reported no significant improvement in activity.^122^ In contrast, Monga *et al.* 2014 made a comparison between nitrochalcones and their corresponding pyrazolines. Interestingly, the pyrazoline counterparts (49b) demonstrated a better activity profile compared with the corresponding chalcones (49a)^123^ (Fig. 20).

**Fig. 21:**
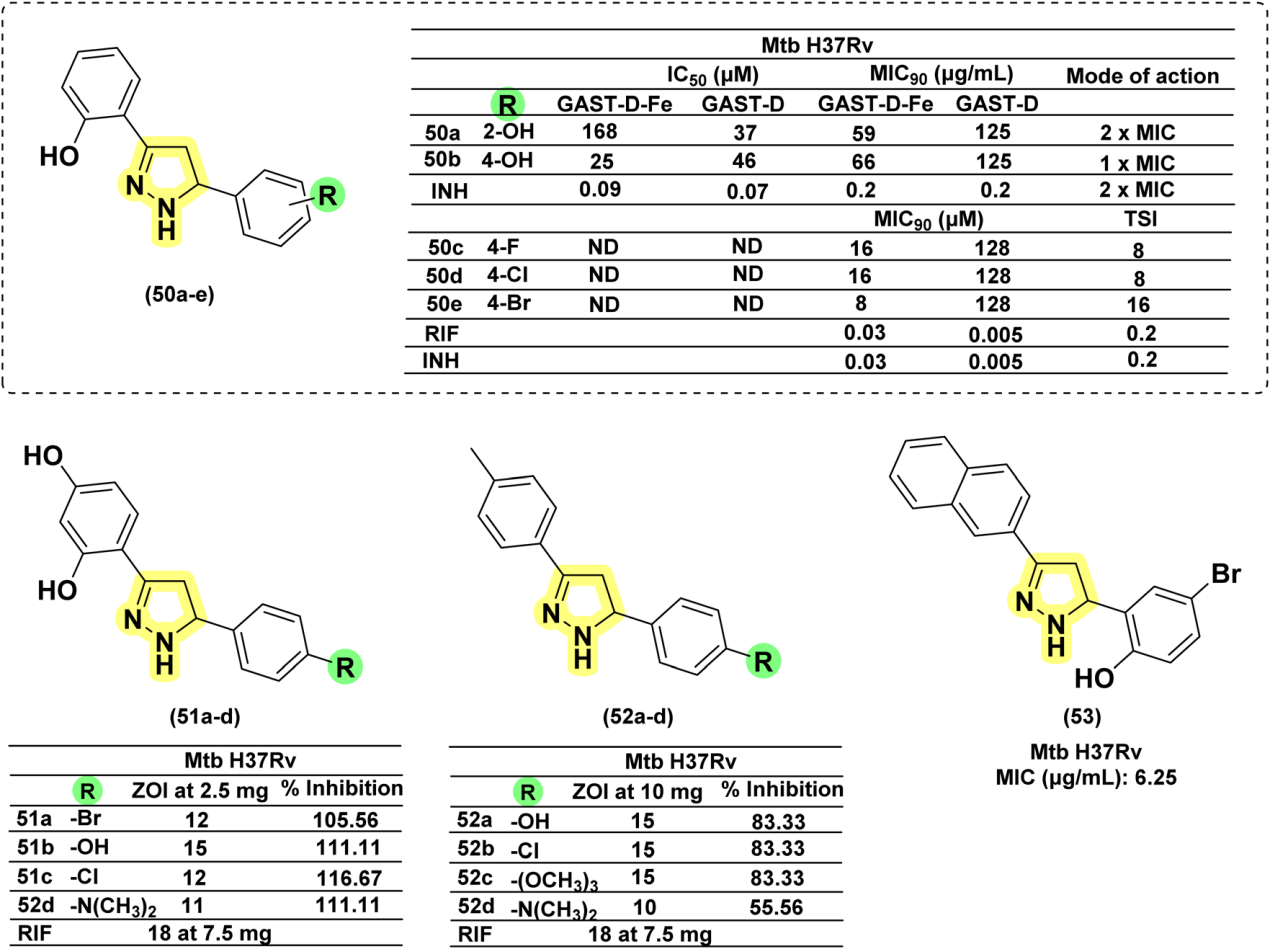
Chemical structures and anti-tubercular profiles of 3,5 di-substituted pyrazolines-based analogues. Yellow highlights the pyrazoline ring system, and green indicates the alkyl/aryl substitutions (ND: not determined).

In 2011, Ferreras *et al.* reported antimycobacterial activity for two of their pyrazoline intermediates in an attempt to identify compounds binding with conditionally essential proteins expressed by *Mtb* and *Yersinia pestis* during iron stress. The structures of these pyrazoline derivatives mimic the bidentate hydroxyphenyloxazoline/thiazoline portion of siderophores secreted by *Mtb*/*Y. pestis*. Both the compounds (50a–b) were found to be active at a concentration below 50 μM. The study design revealed that they were bactericidal and act by inhibiting essential proteins and not by inhibiting conditionally essential proteins expressed during iron stress.^74^ Further, Mousumi *et al.* continued exploring the scaffold with different substitutions over the phenyl ring at the 5^th^ position of pyrazoline. Out of fourteen newly synthesized analogues, nine were found to show improved potency in iron-deprived media (GAST) in comparison with iron-supplemented media (GAST-Fe). Three compounds having halo substitutions –F (50c), –Cl (50d) & –Br (50e) at the *para*-position of the phenyl ring were found to have improved potency, having MIC_90_ of 8–16 μg mL^−1^ and target selectivity index (TSI) of ∼8–16-fold amongst the fourteen compounds synthesized. Through thermofluorimetric analysis, the authors demonstrated the binding of these compounds (50d–e) with salicyl-AMP ligase (MbtA), an enzyme in the biosynthetic pathway of mycobactin. Further, the compounds were also found to be efflux pump inhibitors displaying activity better than verapamil and chlorpromazine.^73^ Five compounds similar to mycobactin mimics reported by Mousumi *et al.* were reported by Jain *et al.* The compounds differ from the one reported by Mousumi *et al.* by having 2,4-dihydroxyphenyl substitution at the third position of pyrazoline instead of 2-hydroxyphenyl. However, the compounds were tested for antitubercular activity in conventional nutrient media by the disc diffusion method. Four of them (51a–d) were found to show 100% inhibition at a 2.5 mg concentration. Another five compounds (52a–e) having 4-methylphenyl substitution at the third position of pyrazoline were found to be less potent than 2,4-dihydroxyphenyl counterparts.^124^ Three more compounds of the same kind were reported by Pola *et al.*, and the best one (53) had an MIC at a concentration of 6.25 μM (ref. 120) (Fig. 21).

##### 1.3.3.2 Heterocycles at 3^rd^ position

**Fig. 22:**
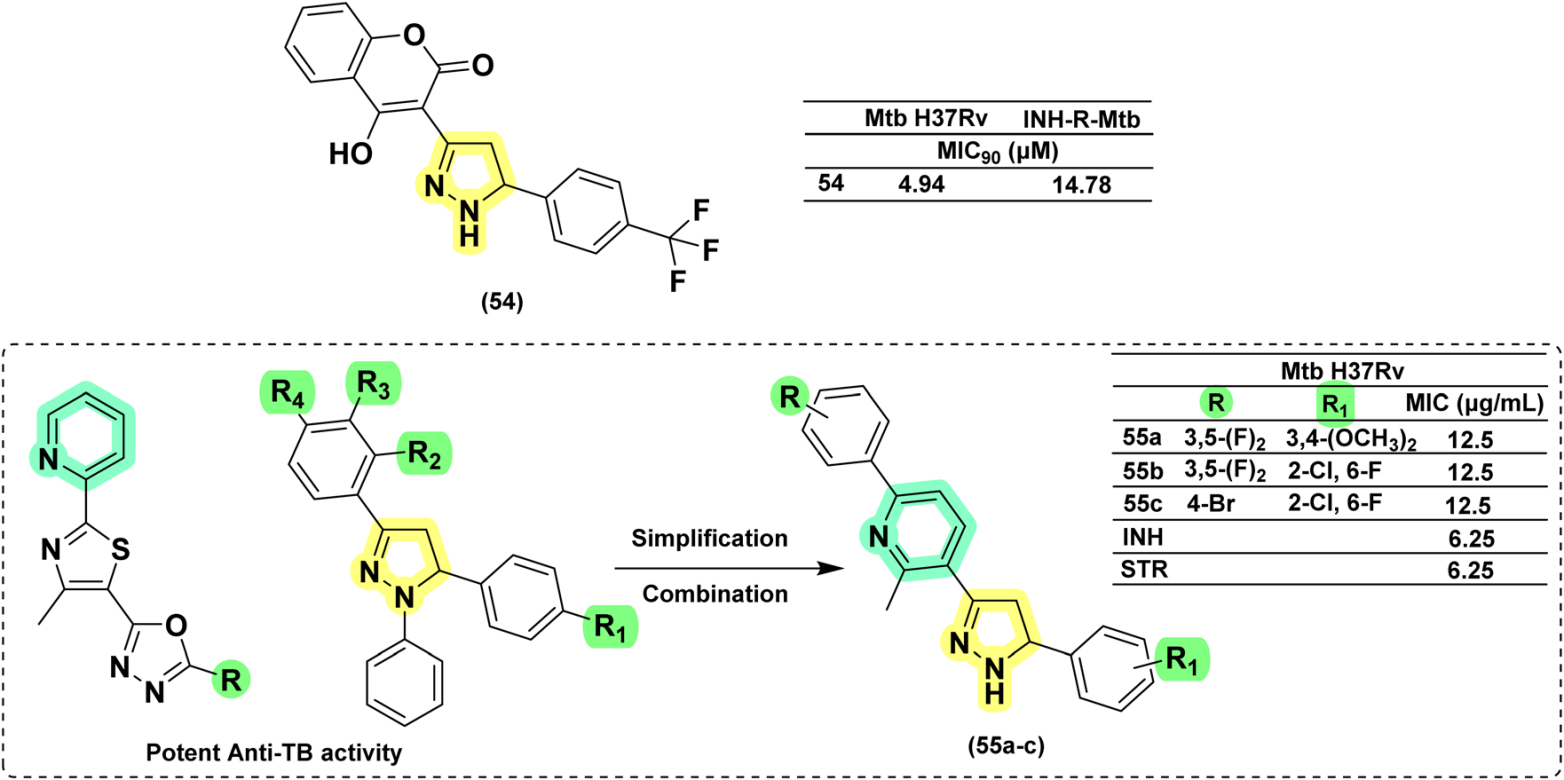
Chemical structures and anti-tubercular profiles of heterocycles at 3rd position-based analogues. Yellow highlights the pyrazoline ring system, green indicates the alkyl/aryl substitutions, and cyan represents the features incorporated in the design strategy.

In 2014, Asad and his research group reported the synthesis of twenty pyrazoline derivatives having 2-chromenone at the 3^rd^ position of pyrazoline. The structures of these new compounds were thoroughly characterized using extensive IR, NMR, and X-ray crystallographic studies. Upon evaluation against *Mtb* H37Rv and INH-resistant *Mtb* strains, compound (54) demonstrated the highest efficacy, showing greater than 90% inhibition against *Mtb* at a concentration of 4.94 μM and against INH-resistant *MTB* at 14.78 μM.^125^ In 2017, Sowmya *et al.* designed twelve pyrazolines having a pyridine ring at the 3^rd^ position of pyrazoline based on the structure of potent compounds reported by Sivakumar *et al.* and Dhumal *et al.*^126,127^ They found three derivatives (55a–c) active at 12.5 μg mL^−1^ but 2-fold less potent than INH and Streptomycin and ∼3–4-fold less potent than ciprofloxacin^128^ (Fig. 22).

##### 1.3.3.3 1,3,5-Triphenyl pyrazolines

**Fig. 23:**
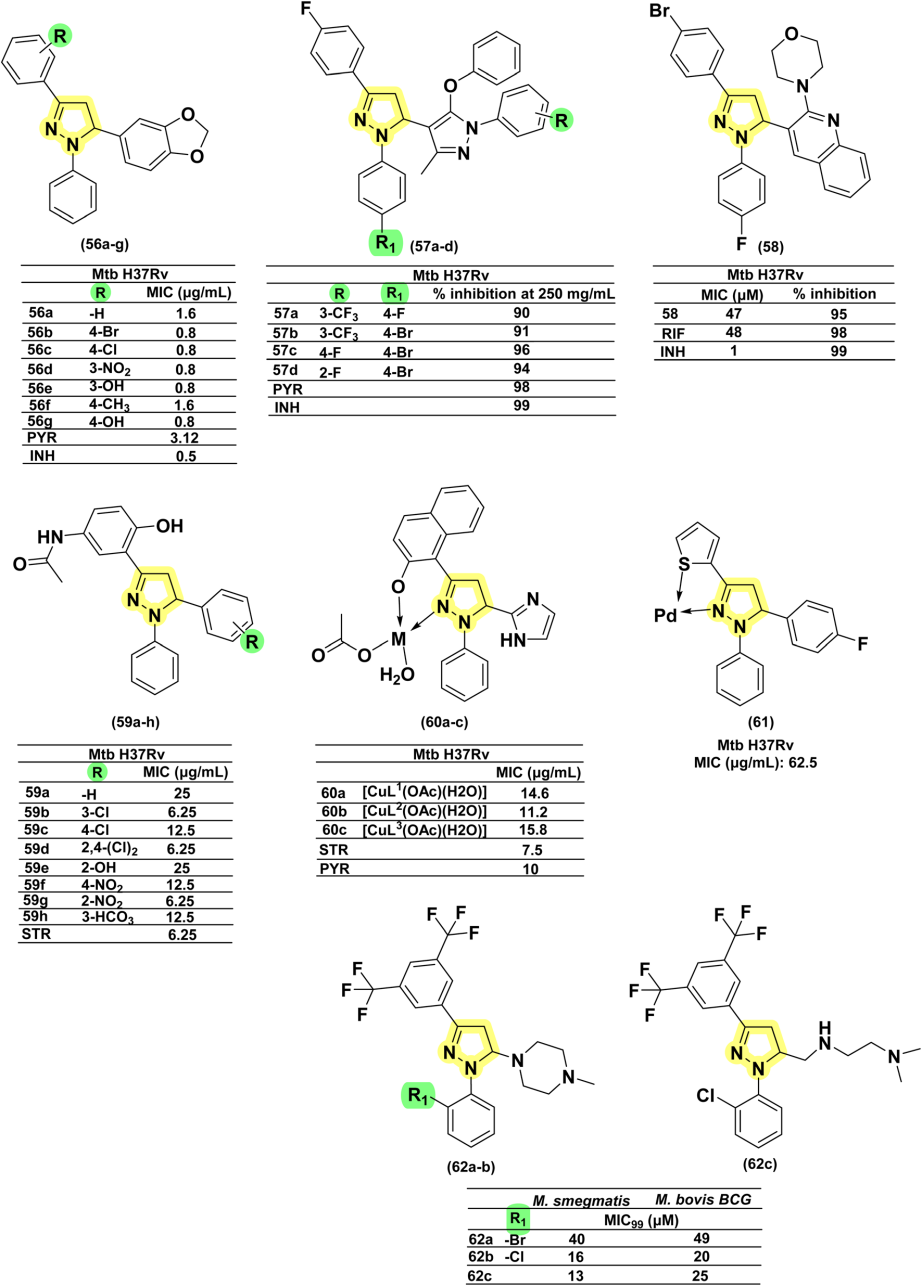
Chemical structures and anti-tubercular profiles of *N*-phenyl pyrazolines-based analogues. Yellow\ highlights the pyrazoline ring system, green indicates the alkyl/aryl substitutions. Ensure consistency in structures.

**Fig. 24:**
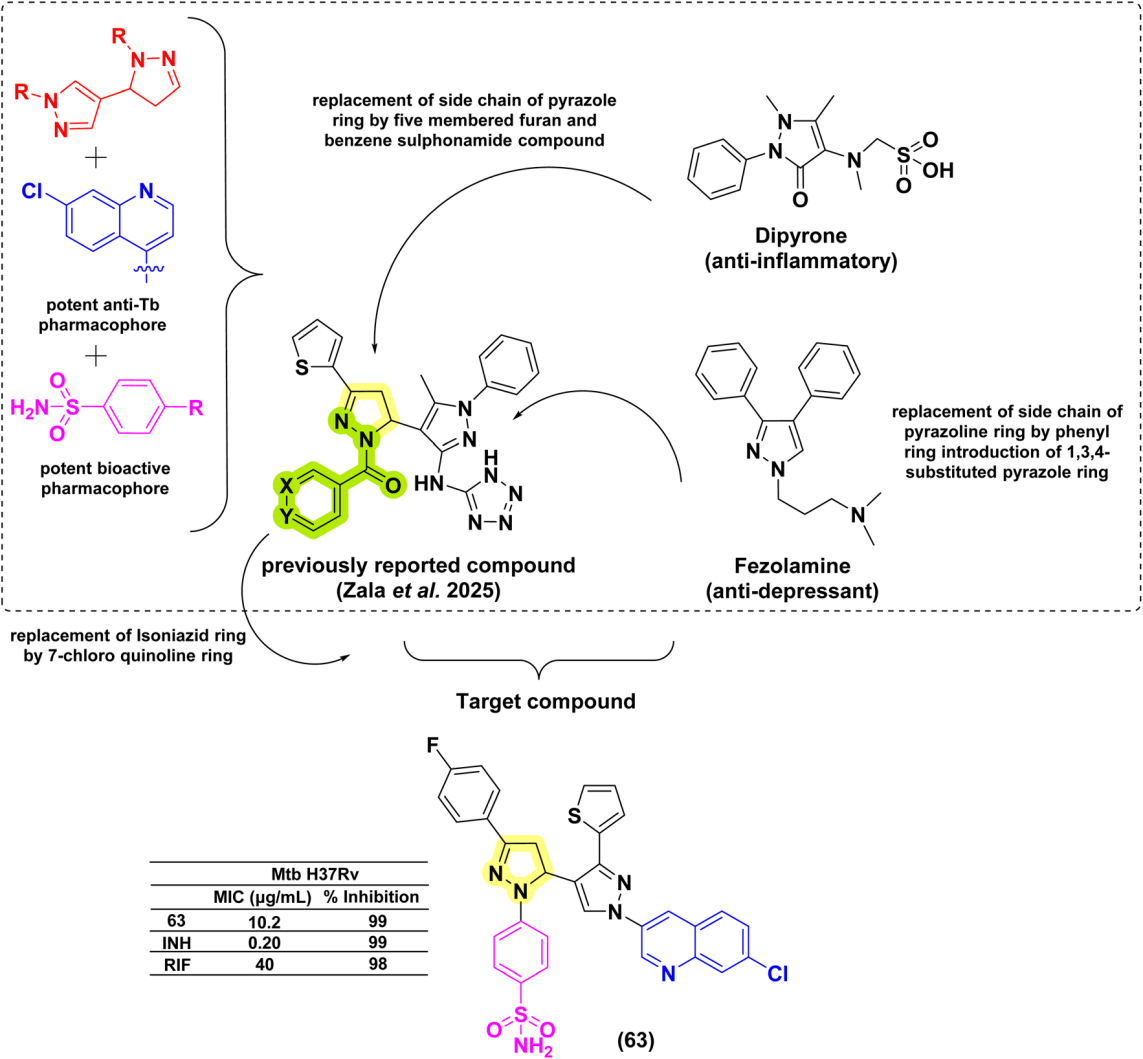
Chemical structures and anti-tubercular profiles of *N*-phenyl pyrazolines-based analogues. Yellow highlights the pyrazoline ring system, other colours mark the features incorporated in the design strategy.

In 2014, Deshpande *et al.* designed a series of seven 1,3,5-triphenyl pyrazolines along with seven acetyl pyrazoline analogues and evaluated them against *Mtb* H37Rv. There was no significant difference in the activity profile between phenyl and acetyl counterparts (refer to acetyl pyrazolines). The activity of these compounds (56a–g) in terms of MIC was in the range of 0.8–1.6 μg mL^−1^. However, the overall cytotoxicity for these compounds was found to be in the range of 1–30% at a concentration range of 50–200 μg mL^−1^, suggesting their safer profiles.^129^ Karad *et al.*, in 2014, reported twelve pyrazolines with a fluorinated phenyl ring and substituted pyrazole at the third and fifth positions of the pyrazolines, respectively. Upon evaluation against *Mtb* H37Rv at a concentration of 250 μg mL^−1^, four compounds (57a–d) displayed good inhibition profiles ranging from 90–96%. The compounds having 4-Br substitution on the phenyl ring at the first position of the pyrazoline (57a) displayed activity equivalent to the standard drugs, pyrazinamide (98%) and INH (99%), used in the study.^130^ The same group in 2016 reported a series of four compounds by shifting the phenyl groups present in the first and third positions of the pyrazoline ring. Additionally, they replaced the pyrazole heterocycle with quinoline in the fifth position of the pyrazoline ring. In this case also, they found the compound having a 4-Br (58) substitution as a potent candidate. Replacing the phenyl ring at the first position of pyrazoline with thiocarbamoyl substitution provided a compound with equal potency. While replacement with either acetyl or formyl led to a drastic reduction in activity (compounds 22a–b).^131^ Ahmad *et al.* in 2014 reported *N*-phenyl pyrazolines conjugated with paracetamol at the third position of the pyrazoline, along with *N*-pyridine-4-carbonyl analogues (29a–b, 30–31, 32a–d) (derived from INH: refer to INH drug conjugate section). The phenyl pyrazoline counterparts (59a–h) were found to be inferior when compared with pyrazolines derived from INH.^132^ Muneera *et al.* in 2016, designed 2-hydroxy naphthyl pyrazolines similar to the one reported by Stirrett and Ferreras *et al.*, having a bidentate feature capable of chelating metallic cations. They evaluated the copper complexes of the designed ligands (60a–c) for antitubercular activity. The antitubercular activity was in the range of 11.2–15.8 μg mL^−1^, and there was no significant difference in activity. The activity was comparable to standard drugs, streptomycin and pyrazinamide.^133^ Thakor *et al.*, in 2018, reported a similar bidentate featuring pyrazolines, replacing 2-hydroxy naphthyl substitution with thiophene at the third position of the pyrazoline ring. The palladium complex of these pyrazolines was evaluated for antitubercular activity, and the ligand having 4-fluorophenyl substitution at the fifth position of the pyrazoline (61) was found to be active.^134^ In 2025, Cui *et al.* reported a fluorescent amino-pyrazoline compound 62a as a promising hit, showing inhibitory activity against *M. smegmatis* (MIC_99_ = 40 μM) and *M. bovis* BCG (MIC_99_ = 49 μM). In an effort to enhance the biological activity, a second-generation library of structurally modified derivatives was synthesized through rational scaffold optimization. Among them, 62c and 62b emerged as the most potent inhibitors, exhibiting significantly improved MIC_99_ values of 13 μM and 25 μM against *M. smegmatis*, and 16 μM and 20 μM against *M. bovis* BCG, respectively. Hence, the electron-withdrawing effect of the di-CF_3_ groups enhances target interactions, while phenyl halogens, such as chlorine at the first position of the pyrazoline ring, provide improved steric compatibility for effective target binding. Additionally, no significant activity differences were observed between *R*, *S*, and racemic forms, indicating that stereochemistry has minimal impact on antibacterial activity.^57^ Zala *et al.* (2025) reported seven novel 7-chloroquinoline-based sulfonamide–pyrazolylpyrazoline hybrids. They modified the pyrazoline ring by replacing dipyrone side chains with furan and benzenesulfonamide groups, and altered the pyrazole ring of fezolamine to include a phenyl ring within a 1,3,4-substituted framework. Additionally, isoniazid and tetrazole moieties in a previously synthesized compound were replaced with 7-chloroquinoline and benzenesulfonamide units. Compound 63 displayed potent inhibitory activity against the *Mtb* H37Rv strain^135^ (Fig. 23 and 24).

#### 1.3.4. Class 4

##### 1.3.4.1 1-Sulphonyl pyrazolines

**Fig. 25:**
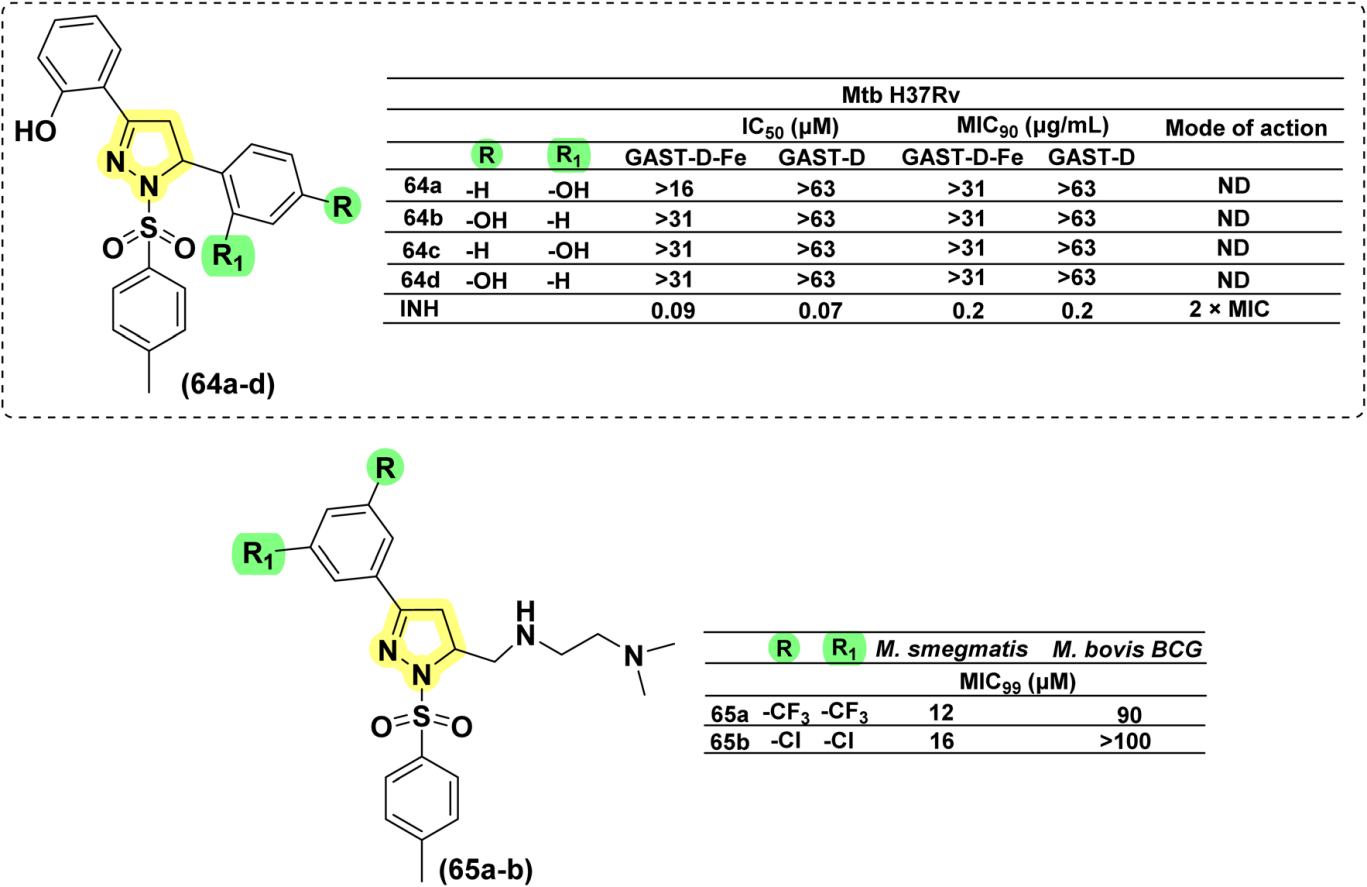
Chemical structures and anti-tubercular profiles of 1-sulphonyl pyrazolines-based analogues.

In 2011, Ferreras *et al.* reported antimycobacterial activity for four sulphonyl pyrazolines in an attempt to identify compounds binding with conditionally essential proteins expressed by *Mtb* and *Y. pestis* during iron stress. The structures of these pyrazoline derivatives mimic the bidentate hydroxyphenyloxazoline/thiazoline portion of siderophores secreted by *Mtb*/*Y. pestis*. All the compounds (64a–d) were found to be inactive. The study design revealed that they act by inhibiting essential proteins and not by inhibiting conditionally essential proteins expressed during iron.^74^ In 2025, Cui *et al.* discovered a novel fluorescent aminopyrazoline derivative through a rational, structure-based design and optimization approach. Using compound 62a as a potent scaffold (refer to 1,3,5-triphenyl pyrazoline section), a tosyl group was introduced at the first position of the pyrazoline ring to yield compounds 88a and 88b. Compound 65a demonstrated the highest activity against *M. smegmatis* with a MIC_99_ of 12 μM, while exhibiting only moderate activity against *M. bovis* BCG, with a MIC_99_ of 90 μM. Replacing the aryl group with a tosyl group improves potency against *M. smegmatis* but decreases effectiveness against *M. bovis* BCG. A similar pattern was observed for compound 65b, which showed a MIC_99_ of 16 μM against *M. smegmatis*, but a marked reduction in activity against *M. bovis* BCG (MIC_99_ > 100 μM). The authors suggested that since *M. smegmatis* is a fast-growing mycobacterium, the greater potency of amino-pyrazolines against this strain compared to *M. bovis* BCG likely arises from differences in cell wall permeability or metabolic processes (Fig. 25).^57^

##### 1.3.4.2 Overview of research methodologies (methods of biological evaluation, *in vivo* studies, docking analyses, and biological targets) for pyrazoline derivatives discussed in this review

The biological evaluation, including *in vitro*, *in vivo*, and *in silico* assays, supports the potential of the designed and synthesized molecules as promising leads or hits for antitubercular drug discovery. Accordingly, a concise overview of the *in vitro* biological evaluation methods employed by various research groups has been presented. In addition, select studies have reported *in vivo* assessments of the most potent compounds identified through initial *in vitro* screening, alongside *in silico* analyses to validate their binding affinity and interaction with target proteins. The standard reference drugs used across these evaluations have also been documented. Table 4 provides a concise overview of these biological evaluations in tabular format.

**Table 4:** A concise summary of the biological evaluations of pyrazoline derivatives, including *in vitro*, *in vivo*, and *in silico* studies. The table highlights assay types, test organisms or targets, standard reference drugs used, and the year of study

Sl no.	Authors	Methods of biological evaluation	*In vivo* studies	Target of docking studies	Standard drug used
1	Arthur *et al.* 1975 (ref. 37)	*Mtb* H37Rv using a tube dilution technique	NA	NA	Isoniazid
2	Küçükgüzel *et al.* 2002 (ref. 28)	*Mtb* (strain H37Rv) using the BACTEC 460 radiometric system, followed by MIC determination *via* Alamar Blue-based broth microdilution assay	NA	NA	Tobramycin
3	Zitouni *et al.* 2005 (ref. 99)	BACTEC 460 radiometric system and BACTEC 12B medium	NA	NA	
4	Shaharyar *et al.* 2006 (ref. 106)	Agar dilution method	NA	NA	Isoniazid
5	Ali *et al.* 2007 (ref. 70)	BACTEC-460 radiometric system and agar dilution method	NA	NA	Isoniazid
6	Ali *et al.* 2007 (ref. 119)	BACTEC-460 micro-dilution assay with Almar Blue	NA	NA	Isoniazid
7	Ali *et al.* 2008 (ref. 71)	BACTEC460 radiometric system	NA	NA	Isoniazid
8	Özdemir *et al.* 2008 (ref. 100)	BACTEC-12B medium using a Broth microdilution assay	NA	NA	Rifampicin
		Microplate Alamar Blue assay (MABA)			
9	Stirrett *et al.* 2008 (ref. 72)	The resazurin microtiter assay determined the MIC_90_ of compounds against *Mtb* H37Rv screened under iron-deficient (GAST) and iron-rich (GAST-Fe, supplemented with 100 μM FeCl_3_) media	NA	NA	Rifampicin
10	Sivakumar *et al.* 2010 (ref. 127)	*Mtb* (H37Rv) by LRP (Luciferase Reporter Phage) assay	NA	NA	
11	Kasabe and Kasabe 2010 (ref. 115)	*Mtb* (strain H37Rv) using REMA plate method	NA	NA	Isoniazid
12	Sharma *et al.* 2010 (ref. 101)	The compounds were added to the Lowenstein–Jensen egg medium at 100 μg mL^−1^ and inoculated with *Mtb* H37Rv strains, then incubated at 37 °C	NA	NA	NA
13	Manna and Agrawal 2010 (ref. 110)	The *in vitro* antitubercular activity was assessed by measuring the growth of *Mtb* (H37Rv) and a multidrug-resistant H37Rv strain using Lowenstein–Jensen (L.–J.) medium	The antitubercular activity of test compounds was studied in six-week-old female CD-1 mice infected with *Mtb* ATCC 35801. Test compounds (25 mg kg^−1^) were evaluated for their ability to reduce bacterial loads in lung and spleen tissues compared to standard drugs and a negative control. Viable bacterial counts were determined by serial 10-fold dilutions followed by inoculation onto 7H10 agar plates. The test compounds showed significant bacterial reductions, comparable to standard treatments	NA	Rifampicin and isoniazid
14	Manna and Agrawal 2011 (ref. 59)	The *in vitro* antitubercular activity was screened against *Mtb* using egg-based Lowenstein–Jensen and Ogawa media. The growth of *Mtb* (H37Rv) was measured after 3 and 6 weeks, with cultures incubated at 37 °C in ambient air for up to 6 weeks	The antitubercular activity of test compounds was studied in six-week-old female CD-1 mice infected with *Mycobacterium tuberculosis* ATCC 35801. Test compounds (25 mg kg^−1^) were evaluated for their ability to reduce bacterial loads in lung and spleen tissues compared to standard drugs and a negative control. Viable bacterial counts were determined by serial 10-fold dilutions followed by inoculation onto 7H10 agar plates. The test compounds showed significant bacterial reductions, comparable to standard treatments	NA	Rifampicin and gatifloxacin
15	Taj *et al.* 2011 (ref. 136)	*Mtb* (strain H37Rv) using the tube dilution method in Middlebrook 7H9 broth	NA	NA	Streptomycin and pyrazinamide
16	Ali *et al.* 2011 (ref. 76)	The compounds were added to the Lowenstein–Jensen egg medium at 100 μg mL^−1^ and inoculated with *Mtb* H37Rv strains, then incubated at 37 °C	NA	NA	NA
17	Ahsan *et al.* 2011 (ref. 137)	*In vitro* antimycobacterial activity against *Mtb* and INH^R^-*Mtb* using the agar dilution method. Active compounds were further tested for cytotoxicity (IC_50_) in VERO cells at 62.5 μg mL^−1^ or 10 times the MIC	NA	NA	Rifampicin and isoniazid
18	Ahsan *et al.* 2011 (ref. 77)	Agar dilution method using double dilution technique	NA	NA	Rifampicin and isoniazid
19	Ahsan *et al.* 2011 (ref. 85)	Agar dilution method using Middlebrook 7H11 broth medium	NA	NA	Isoniazid
20	Ferreras *et al.* 2011 (ref. 138)	The resazurin microtiter assay determined the MIC_90_ of compounds against *Mtb* H37Rv screened under iron-deficient (GAST) and iron-rich (GAST-Fe, supplemented with 100 μM FeCl_3_) media	NA	NA	Isoniazid
21	Shelke *et al.* 2012 (ref. 139)	The H37Rv strain was cultured in L.–J. medium (1 mg per mL inoculum). Primary screening used concentrations of 500, 250, and 125 μg mL^−1^, with further testing at 100–1.5625 μg mL^−1^. MIC was defined as the concentration inhibiting 99% of growth	NA	NA	Isoniazid
22	Ali *et al.* 2012 (ref. 104)	BACTEC 460 radiometric system	NA	NA	Isoniazid
23	Hazra *et al.* 2012 (ref. 78)	Evaluated against *Mtb* H37Rv (ATCC 27294) using Middlebrook 7H-9 broth. Growth inhibition of *Mtb* species was assessed by Ziehl–Neelsen staining	NA	NA	Pyrazinamide
					Streptomycin
24	Jain *et al.* 2013 (ref. 124)	The antimicrobial activity was determined using the standard Kirby–Bauer disk diffusion method. *Mtb* was inoculated in the nutrient broth and incubated overnight at 37 °C. The bacterial culture was then spread on a solidified Muller–Hinton agar plate, and 6 mm wells were punched. Different concentrations of the derivatives (2.5, 5, 7.5, and 10 mg) were dispensed into the wells, with 100 μL of DMSO added to the centre well as a control. The plates were incubated at 2537 °C for 24 hours, and the zone of inhibition around the wells was measured and recorded	NA	NA	Rifampicin
25	Dharmarajsinh *et al.* 2014 (ref. 91)	The *in vitro* antitubercular activity was assessed by measuring the growth of *Mtb* (H37Rv) using the Lowenstein–Jensen (L.–J.) medium	NA	InhA (PDB ID 2H7I)	Isoniazid
26	Ahmad *et al.* 2014 (ref. 132)	The agar microdilution method was used, where twofold dilutions of each test compound were added to the 7H11 agar medium supplemented with OADC (oleic acid, albumin, dextrose, and catalase) and the organism	NA	NA	Streptomycin
27	Dharmarajsinh *et al.* 2014 (ref. 92)	The *in vitro* antitubercular activity was assessed by measuring the growth of *Mtb* (H37Rv) using the Lowenstein–Jensen (L.–J.) medium	NA	InhA (PDB ID 2H7I)	Isoniazid
28	Kalaria *et al.* 2014 (ref. 140)	The *in vitro* antitubercular activity (250 μg mL^−1^ and 100 μg mL^−1^) was assessed by measuring the growth of *Mtb* (H37Rv) using the Lowenstein–Jensen (L.–J.) medium	NA	NA	Rifampicin and isoniazid
29	Monga *et al.* 2014 (ref. 87)	The MIC of the test compounds against *Mtb* H37Rv was determined using the L.–J. agar method	NA	NA	Rifampicin
30	Karad *et al.* 2014 (ref. 130)	The *in vitro* antitubercular activity was assessed by measuring the growth of *Mtb* (H37Rv) using the Lowenstein–Jensen (L.–J.) medium	NA	NA	Rifampicin and isoniazid
31	Asad *et al.* 2014 (ref. 125)	Agar dilution method	NA	NA	Isoniazid
32	Deshpande *et al.* 2015 (ref. 129)	Microplate Alamar Blue assay (MABA)	NA	NA	Pyrazinamide and isoniazid
33	Napoleon *et al.* 2015 (ref. 60)	Microplate Alamar Blue assay (MABA)	NA	NA	Pyrazinamide and isoniazid
34	Joshi *et al.* 2016 (ref. 94)	Microplate Alamar Blue assay (MABA)	NA	InhA (PDB ID 4TZK)	Ethambutol and rifampicin
35	Muneera and Joseph 2016 (ref. 133)	Broth dilution assay method	NA	NA	Pyrazinamide
36	Solankee *et al.* 2016 (ref. 116)	The *in vitro* antitubercular activity was assessed by measuring the growth of *Mtb* (H37Rv) using the Lowenstein–Jensen (L.–J.) medium	NA	NA	Rifampicin and isoniazid
37	Karad *et al.* 2016 (ref. 79)	The *in vitro* antitubercular activity was assessed by measuring the growth of *Mtb* (H37Rv) using the Lowenstein–Jensen (L.–J.) medium	NA	NA	Rifampicin and isoniazid
38	Dixit *et al.* 2017 (ref. 95)	Microplate Almar Blue assay (MABA)	NA	InhA (PDB ID 4TZK)	Pyrazinamide and streptomycin
39	Rao *et al.* 2017 (ref. 118)	Positive candidates from the preliminary screening were further tested against *Mtb* (H37Rv) and rifampicin-resistant *Mtb* (RifR) to determine the minimum inhibitory concentration (MIC) using the Broth microdilution assay	NA	NA	Rifampicin
40	Sadashiva *et al.* 2017 (ref. 82)	Microplate Almar Blue assay (MABA)	NA	NA	Pyrazinamide, streptomycin, and ciprofloxacin
41	Sowmya *et al.* 2017 (ref. 128)	Microplate Almar Blue assay (MABA)	NA	NA	Isoniazid, streptomycin, and ciprofloxacin
42	Hallikeri *et al.* 2017 (ref. 96)	Microplate Almar Blue assay (MABA)	NA	NA	Pyrazinamide
43	Thakor *et al.* 2018 (ref. 134)	The MIC of the test complexes against *Mtb* H37Rv was determined using the Lowenstein–Jensen agar method	*Schizosaccharomyces pombe* was used to assess cytotoxicity *via* trypan blue staining. Test compounds showed maximum cytotoxicity, increasing with concentration. Chelation with Pd(ii) enhanced cytotoxicity, surpassing pyrazoline-based Ru(iii) complexes	B-DNA (PDB ID 1BNA)	Rifampicin and isoniazid
44	Venkata *et al.* 2019	Broth dilution assay	NA	*Mtb* InhA structures (PDB IDs 2NSD & 3FNG)	Pyrazinamide
45	Pola *et al.* 2020 (ref. 120)	*Mtb* (H37Rv) using a growth inhibition assay by turbidimetry	NA	InhA (PDB ID 4TZK)	Rifampicin and isoniazid
46	Wong *et al.* 2021 (ref. 80)	The antitubercular activity against *Mtb* (H37Rv) was evaluated using the Tetrazolium Bromide Microplate Assay (TEMA) method	NA	CYP51 (PDB 1EA1)	Isoniazid
47	Castaño *et al.* 2022 (ref. 114)	Antituberculosis activity (*Mycobacterium bovis* and *Mtb* H37Rv) was carried out using the agar dilutions pot culture growth inhibition assay	NA	NA	Isoniazid
48	Shyam *et al.* 2022 (ref. 141)	The resazurin microtiter assay determined the MIC_90_ of compounds against *M. smegmatis*, *M. aurum*, *M. bovis* BCG, and *M. tuberculosis*, screened under iron-deficient (GAST) and iron-rich (GAST-Fe, supplemented with 200 μM FeCl_3_) media	*In vivo* pharmacokinetic profiling of top compounds exhibited high plasma clearance (84.8 and 100 mL min^−1^ kg^−1^) and significant tissue distribution (11.5 and 17.3 L kg^−1^) in male Sprague-Dawley rats. After oral dosing (5 mg kg^−1^), both reached *T*_max_ at 0.25 hours, with *C*_max_ values of 51.6 ng mL^−1^ (ref. 44) and 48.8 ng mL^−1^,^49^ and AUC_last_ of 203 ng h mL^−1^ and 167 ng h mL^−1^. Oral bioavailability was similar: 21% and 22%	MbtA (PDB ID 5KEI)	Rifampicin and isoniazid
49	Rasgania *et al.* 2024 (ref. 112)	The antitubercular activity against *Mtb* (H37Rv) was evaluated using the Microplate Alamar Blue Assay (MABA)	NA	InhA (PDB ID 2X23)	Isoniazid and Triclosan
50	Cui *et al.* 2025 (ref. 57)	The resazurin microtiter assay determined the MIC_99_ of compounds against *M. smegmatis* and *M. bovis* BCG	NA	Ag85C as the primary target, disrupting late-stage mycolic acid biosynthesis and compromising cell wall integrity (PDB ID 1DQZ)	Rifampicin
51	Tailor *et al.* 2025 (ref. 117)	The disc diffusion method was performed on Mueller–Hinton agar, followed by incubation at 37 °C for 24 hours. Inhibition zone diameters were measured in mm. The MICs of the most active compounds were determined using the twofold dilution method against *Mtb* H37Rv	NA	Glucosyl-3-phosphoglycerate phosphatase (GPGP) (PDB ID 4PZA) from *Mtb*	Rifampicin and isoniazid
52	Zala *et al.* 2025 (ref. 135)	The *in vitro* antitubercular activity was assessed by measuring the growth of *Mtb* (H37Rv) using the Lowenstein–Jensen (L.–J.) medium	NA	Enoyl acyl carrier protein reductase (ENR) from *Mtb*	Rifampicin and isoniazid

The primary model organism used to perform antitubercular assays was *Mtb* H37Rv.^59,78,110,115,139^ This is likely because it is the most reliable and widely accepted strain for such assays and is readily available in most research laboratory settings. The *in vitro* assays were primarily conducted using the BACTEC 460 radiometric system,^70,71,76,100,104,105,107^ an automated system, and the Microplate Alamar Blue Assay (MABA),^60,93,94,96,128^ as reported by various research groups. Notably, one group (Shyam *et al.*, Ferreras *et al.*, and Stirrett *et al.*) focused on the concept of iron regulation in mycobacterium species and therefore utilized iron-deficient and iron-rich GAST media along with the resazurin-based assay for their *in vitro* studies.^72,138,141^ Both research groups extended their investigations beyond *Mtb*, evaluating activity against additional mycobacterial strains such as *M. smegmatis*, *M. aurum*, and *M. bovis* BCG. They also explored target-specific inhibition at the enzyme level. One group assessed their compounds against the siderophore biosynthesis enzymes MbtA and MbtB, supported by crystallographic studies of the most potent inhibitors. Cui *et al.* similarly evaluated their compounds against Ag85C, a key mycolyltransferase involved in late-stage mycolic acid biosynthesis.^57^ Their lead compound demonstrated a 46% reduction in Ag85C activity at 50 μM, indicating effective enzyme inhibition. Both groups employed various antimicrobial assays, including the Luciferase Reporter Phage (LRP) assay, agar dilution, and microdilution methods, to determine MIC values. Furthermore, Cui *et al.* assessed the intracellular efficacy of pyrazoline derivatives using J774 murine macrophages, highlighting their potential as intracellular antimycobacterial agents. Very few groups performed *in vivo* assays to evaluate the potential of their molecules in preclinical settings.^59,110^*In vivo* antitubercular activity of test compounds was assessed in six-week-old female CD-1 mice infected with *Mtb* ATCC 35801. Compounds (25 mg kg^−1^) were tested for their ability to reduce bacterial loads in lung and spleen tissues, with results compared to standard drugs and a negative control. Viable bacterial counts were determined *via* serial dilutions and inoculation onto 7H10 agar plates. Test compounds exhibited significant bacterial reductions, comparable to standard treatments. *In vivo* studies were also conducted to evaluate the cytotoxicity and pharmacokinetics of the potent molecules. Cytotoxicity was assessed using *Schizosaccharomyces pombe* through trypan blue staining.^134^*In vivo* pharmacokinetic profiling was performed in male Sprague-Dawley rats following oral dosing. Parameters such as *T*_max_, *C*_max_, AUC, oral bioavailability, plasma clearance, and tissue distribution were evaluated.^141^ The test results were primarily compared with standard frontline antitubercular drugs, namely isoniazid, rifampicin, pyrazinamide, ethambutol, streptomycin, and ciprofloxacin.

##### 1.3.4.3 Target orientation in antitubercular drug discovery: pyrazoline scaffold

Target orientation was presented by the authors in general and was identified as three key targets: InhA, cytochrome P450 14 alpha-sterol demethylase, and the mycobactin biosynthesis pathway.

##### 1.3.4.4 InhA or 2-trans-enoyl-acyl carrier protein reductase

InhA, or 2-trans-enoyl-acyl carrier protein reductase, is a critical enzyme in the fatty acid elongation cycle of *Mtb*, responsible for the synthesis of mycolic acids, key components of the bacterial cell wall.^142^ It serves as the primary target of the first-line antitubercular drug INH, a prodrug activated by the bacterial catalase-peroxidase enzyme, *KatG*. Once activated, INH forms an adduct with NADH, which inhibits InhA by blocking its activity. However, mutations in the katG gene or the InhA promoter region often lead to drug resistance, posing significant challenges in Tb treatment. Consequently, direct InhA inhibitors that bypass the need for activation by *KatG* are being developed to address multidrug-resistant tuberculosis. As a vital enzyme in mycolic acid biosynthesis and a central player in antimicrobial resistance, InhA remains a key target in tuberculosis drug discovery and research. In line with this, various research groups have designed, synthesized, and evaluated pyrazoline analogues, as discussed earlier, and assessed their potential using molecular simulations targeting InhA, the enzyme inhibited by INH. These efforts aim to develop isoniazid-like drugs with improved efficacy and the ability to overcome drug resistance.

In 2013, Rana *et al.* conducted molecular docking studies to explore the plausible binding motifs of their pyrazoline-based compounds (compounds 17a–c). They utilized the crystal structure of *Mtb* enoyl reductase (InhA) (PDB code 2H7I) as the target.^91^ The results showed that the ligand was deeply buried within a hydrophobic pocket formed by residues Tyr158, Ile215, Met103, and Met199, minimizing exposure to the solvent. Encouragingly, most of the ligands retained the dispersion interactions observed in the crystal structure (PDB code 2H7I) and also formed additional interactions with residues Ile202, Met155, and Leu218. However, the researchers noted that the Glide score did not correlate well with biological activity (*r*^2^ = 0.04), which they attributed to the simplicity of the molecular mechanics-based scoring function. They further investigated the contribution of the pyrazoline fragments of the target compounds to protein binding. The pyrazoline ring was found to interact with Ala198 and Met199 through dispersion interactions, but the interaction energies were significantly lower. Interestingly, the aryl ring on the pyrazoline fragment faced toward the solvent and lacked interacting partners. This resulted in unfavourable interaction energies, suggesting that the aryl group at the fifth position of the pyrazoline ring might not be necessary. The study proposed that smaller substituents, such as alkyl groups, could replace the aryl group to improve binding. Overall, the designed compounds showed more favourable interactions with the active site residues of InhA than isoniazid, as reflected by their superior Glide scores. A research group led by Joshi and Dixit in 2016 and 2017 performed molecular docking studies using Surflex-Dock (Sybyl-X 2.0) on the InhA enzyme structure (PDB ID 4TZK).^94,95^ The compounds displayed consensus scores ranging from 9.05 to 2.65. Their analysis revealed that substituted pyrrolyl derivatives (compounds 20a–d and 21a) occupied the hydrophobic pocket of InhA. The top-performing molecules formed hydrogen bonds with Met98, Tyr158, and the cofactor NAD+, fitting well within the InhA binding pocket. Notably, these compounds exhibited similar interactions to the original ligand of 4TZK. The OCH_3_, CHO, and C=O groups formed hydrogen bonds with the substrate-binding site, while electron-donating or withdrawing groups on the aromatic ring attached to the pyrazoline moiety enhanced activity. Additionally, the pyrrole, pyrazoline, isoxazole, and phenylthiourea moieties facilitated effective binding and penetration into the active site. Thakor *et al.*, in 2018, conducted molecular modelling studies to investigate the binding mode of compound 61 (pyrazoline-based palladium(ii) compound).^134^ Using HEX 6.0 software, they performed docking studies with B-DNA (PDB ID 1BNA) to determine the theoretical binding energy of the synthesized compounds. The most stable interaction, with a binding energy of −252.61, fit into the G–C-rich minor groove, stabilized by van der Waals and hydrophobic forces. Their analysis suggested an intercalative binding mode of interaction with DNA. In 2019, Bontha Venkata conducted molecular docking (Ligand scout 4.1) for compounds 36a–e with *Mtb* InhA structures (PDB IDs 2NSD, 3FNG). The compounds showed strong ligand interactions and binding affinities, with values ranging from −16.70 to −19.20 kcal mol^−1^ (3FNG) and −9.30 to −11.20 kcal mol^−1^ (2NSD), significantly higher than pyrazinamide (−10.70 and −11.10 kcal mol^−1^, respectively). In 2020, Pola *et al.* conducted molecular docking studies on InhA (PDB ID 4TZK) using Schrödinger software to evaluate the binding modes of active and weakly active compounds.^120^ The most active compound 47a, displayed a strong hydrogen bond interaction with a surrounding water molecule, resulting in a docking score of −10.50 kcal mol^−1^ and docking energy of −44.50 kcal mol^−1^. This interaction contributed to its significant *in vitro* activity compared to the standard INH. Conversely, the weakly active compound lacked crucial hydrogen bond interactions with surrounding amino acid residues, yielding a lower docking score (−6.74 kcal mol^−1^) and energy (−42.50 kcal mol^−1^), and exhibited reduced activity against *Mtb*. In 2024, Rasgania's study further supported the potent antitubercular effect of compound 37 through docking studies using Autodock Vina against the promising antitubercular target InhA (PDB ID 2X23).^112^ The compound exhibited a significant binding of −8.9 kcal mol^−1^ and formed favourable interactions with key residues like Tyr158 and Thr196. Tyr158 is crucial for enoyl-acyl reductase activity, as its binding with a ligand can disrupt the enzyme. Thr196 plays an essential role in the substrate binding loop of InhA. Although no hydrogen bonds were formed, hydrophobic interactions with residues like Phe41, Val65, Ile122, Ile95, Ala198, Leu197, Phe97, Ile202, and Ala201 contribute to binding, playing a key role in inhibiting the target receptor. In 2025, Zala *et al.* conducted molecular docking studies targeting enoyl-acyl carrier protein reductase (ENR) of *Mtb*. Compound 63 exhibited the most favourable docking profile, with a Glide score of −9.714 and a Glide binding energy of −64.183 kcal mol^−1^. Its high binding affinity was attributed to strong electrostatic interactions with key residues Thr196 (−1.099 kcal mol^−1^), Arg195 (−1.063 kcal mol^−1^), Gln100 (−1.053 kcal mol^−1^), and Met98 (−2.517 kcal mol^−1^).^135^

##### 1.3.4.5 Ag85C

Ag85C is identified as the primary molecular target, where its inhibition disrupts late-stage mycolic acid biosynthesis, leading to impaired cell wall integrity. Cui *et al.* in 2025 conducted molecular docking studies using the crystal structure (PDB 1DQZ) to investigate the interaction of amino-pyrazoline derivative (62a) with Ag85C. Compound 62a showed good binding within the active site, with its di-CF_3_ phenyl group occupying the hydrophobic pocket and the aminopyrazoline core forming hydrophilic interactions. Both (*R*)- and (*S*)-enantiomers exhibited similar poses with binding affinities of −10.8 and −10.4 kcal mol^−1^, respectively. Key residues Leu40, Arg41, and Phe76 contributed to ligand stabilization.^57^

##### 1.3.4.6 Glucosyl-3-phosphoglycerate phosphatase (GPGP)

GPGP enzymes are important targets due to their essential roles in bacterial survival and virulence. Tailor *et al.* (2025) conducted molecular docking studies targeting GPGP (PDB ID 4PZA), where compound 43 demonstrated favourable binding energy (−7.95 kcal mol^−1^). It formed key hydrogen bonds with residues Glu29 and Asp63 at distances of 2.87 Å and 2.81 Å, respectively. These interactions positioned the ligand deep within the enzyme's catalytic site, contributing to effective inhibition of its function and subsequent disruption of the bacterial metabolic pathway.^117^

##### 1.3.4.7 Cytochrome P450 14 alpha-sterol demethylase

CYP51 (cytochrome P450 14 alpha-sterol demethylase) in *Mtb* is involved in the sterol biosynthesis pathway. It catalyses the 14-alpha demethylation of sterol intermediates, which is a critical step in producing sterols essential for the integrity and function of the bacterial cell membrane.^143,144^ While *Mtb* does not rely on sterols as extensively as eukaryotes, it still requires them for maintaining the mycomembrane, a unique lipid-rich structure that provides protection against environmental stress and contributes to the bacterium's virulence. Disruption of CYP51 activity impairs the production of functional sterols, leading to compromised membrane integrity and weakening the bacterial cell envelope. This can make the bacterium more susceptible to host immune responses and antimicrobial treatments. Thus, CYP51 plays a key role in the survival and pathogenicity of *Mtb* by ensuring proper membrane composition. In 2021, Wong conducted molecular docking studies on compound 10 targeting alpha-sterol demethylase (CYP51).^80^ The binding energies for CYP51 with fluconazole, isoniazid, and compound 12 ranged from −6.2 to −7.1, −6.0 to −5.0, and −6.3 to −6.7, respectively. Key interactions included pyrazoline with Leu317 and Arg354, hydrophobic interactions with Ile27, Arg274, and Arg247, and a non-conventional hydrogen bond between the thioamide group and Gly84. However, the study requires further investigation into how these interactions contribute to the compound's activity.

##### 1.3.4.8 Mycobactin biosynthesis pathway enzymes

**Fig. 26:**
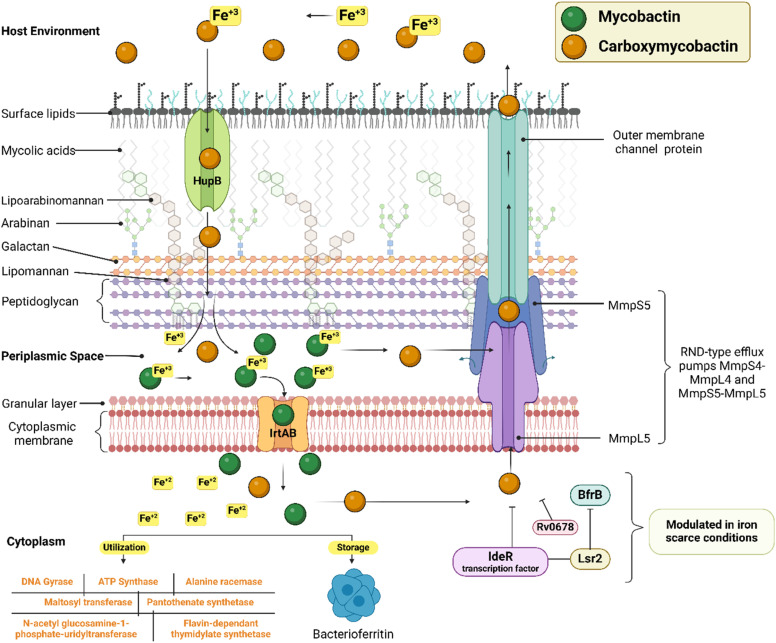
The mycobactin biosynthetic pathway: a promising novel therapeutic target to combat drug resistance in mycobacterial infection (created in BioRender. Agreement number: IF28OH9O0K, Rakshit, G. (2025) https://BioRender.com/f5i661h).

Targeting novel pathways is a crucial strategy to combat drug resistance in tuberculosis. One such conditionally essential pathway is the mycobactin biosynthetic pathway.^145,146^ During infection, alveolar macrophages, the primary site of mycobacterial infection, create an iron-deficient environment as a defence mechanism by sequestering iron.^147,148^ To overcome this, mycobacteria release siderophores like mycobactin and carboxymycobactin.^149,150^ These small hexadentate ligands with high iron affinity play a vital role in iron acquisition.^151^ Carboxymycobactin, a hydrophilic molecule, binds ferric ions in the host environment, converts them to ferrous ions, and internalizes them for use in mycobacterial metabolism.^152^ Any excess iron is stored in bacterioferritin, aiding the bacteria in sustaining growth and replication. Targeting this iron-acquisition pathway offers potential as a therapeutic strategy against drug-resistant TB^146^ (Fig. 26).

In line with this approach, studies by Stirrett *et al.* (2008), Ferreras *et al.* (2011), and Mousumi *et al.* (2022) explored inhibitors resembling the hydroxyphenyl-oxazoline portion of mycobactin, a mycobacterial siderophore.^72,74,141^ Additionally, Mousumi *et al.* (2022) conducted simulation studies targeting MbtA, a salicyl-AMP ligase involved in the first step of siderophore biosynthesis. Mousumi *et al.* (2022) performed molecular dynamics simulations using GROMACS to study compounds 51c–e against MbtA (PDB ID 5KEI), an enzyme involved in the first step of mycobactin biosynthesis. They hypothesized that ligand binding at the Lys546-centered catalytic pocket in the C-terminal domain may restrict substrate binding and inhibit MbtA activity. Compound 51d showed a stronger affinity for MbtA (binding free energy of −25.69 ± 3.39 kcal mol^−1^) compared to 51e. Hydrogen bonding was observed with key residues, including Ala420, Glu423, and Gly545. The study concluded that the ligands form stable complexes with MbtA, potentially inhibiting its catalytic activity by limiting C-terminal domain movement.

##### 1.3.4.9 ADMET aspects for pyrazoline derivatives

The articles recently published in 2025 included predictive ADME studies to evaluate drug-likeness and various other medicinal chemistry parameters. Zala *et al.* (2025) conducted *in silico* pharmacokinetic and toxicity predictions for compound 63 using the QikProp module of Schrödinger software.^135^ The ADME analysis assessed key drug-likeness parameters, including Lipinski's rule of five, which compound 63 successfully met: a molecular weight of 629.125 (<650), a log *P*_o/w_ of 6.59, and a QP log *S* of −9.987, indicating moderate solubility. The compound also showed favourable blood–brain barrier (BBB) permeability with a QP log BB value of −1.123, suggesting potential for central nervous system (CNS) penetration. The predicted QPPMDCK value was 723.122, indicating high membrane permeability through MDCK cells, a model for BBB permeability. Additionally, the QPPCaco value, estimating intestinal absorption, was 244.564 nm s^−1^, remaining well below the 500 nm s^−1^ threshold, suggesting good oral bioavailability. Overall, these properties indicate that the pyrazolylpyrazoline derivative, compound 63, possesses promising ADME characteristics and is a suitable candidate for further optimization in drug development. Cui *et al.* (2025) reported that compound 62c displayed favourable *in silico* ADMET properties *via* ADMETLAB 3.0.^57^ Despite slightly suboptimal Caco-2 permeability, it showed good bioavailability (MW ∼ 492 Da, log *P* 4.91), low P-gp efflux, moderate plasma protein binding (93%), suitable distribution (∼1.08 L kg^−1^), and CNS penetration potential, making it a candidate for TB meningitis. It also demonstrated high metabolic stability (instability probability 0.2), though predicted CYP1A2 and CYP2C19 interactions were noted. Toxicity risks were low, except for a potential hERG interaction (probability > 0.7), which can be addressed through structural optimization. Overall, 62c emerges as a strong antimycobacterial lead. Tailor *et al.* (2025) evaluated the drug-like properties of the lead compound (43) using DataWarrior, ADMETlab 2.0, and SwissADME.^117^ The molecular weight was 538.62 Da, slightly above the ideal range but still acceptable for drug development. It had 7 hydrogen bond acceptors and 1 hydrogen bond donor, supporting good interaction potential with biological targets. The moderate lipophilicity, indicated by *M* log *P* of 4.03 and *W* log *P* of 3.32, suggests favourable membrane permeability. With 3 rotatable bonds, the compound maintains good conformational stability, and its topological polar surface area (TPSA) of 100.24 Å^2^ points to potential oral bioavailability and central nervous system penetration. Importantly, no PAINS alerts were detected, indicating the compound is unlikely to interfere non-specifically in biological assays. Altogether, these properties highlight compound 43 as a promising drug-like candidate.

Shyam *et al.* (2022) conducted *in vivo* pharmacokinetic and pharmacodynamic (PK/PD) studies and identified compound 50d–e, a mycobactin biosynthesis inhibitor, as demonstrating an excellent pharmacokinetic profile.^141^ In male Sprague-Dawley rats, these compounds showed high plasma clearance rates (84.8 and 100 mL min^−1^ kg^−1^) and extensive tissue distribution (11.5 and 17.3 L kg^−1^). Following oral administration at 5 mg kg^−1^, both compounds reached *T*_max_ at 0.25 hours, with *C*_max_ values of 51.6 ng mL^−1^ (compound 50d) and 48.8 ng mL^−1^ (compound 50e), and AUC_last_ values of 203 ng h^−1^ mL^−1^ and 167 ng h^−1^ mL^−1^, respectively. Oral bioavailability was comparable for both compounds, measured at 21% and 22%.

##### 1.3.4.10 Key patents on pyrazoline scaffold as antitubercular agents

Patents safeguard the intellectual property rights of researchers and pharmaceutical companies, granting them exclusive rights to develop and commercialize novel compounds, especially those showing promise against drug-sensitive and drug-resistant strains of *Mtb*, for up to 20 years. Despite the critical need, there have been relatively limited patents filed in this domain recently. Below are some key patents (Table 5) from the last five years that highlight recent advancements in pyrazoline-based compounds as potential antitubercular agents.

**Table 5:** Key patents on pyrazoline scaffold as antitubercular agents

Sl. no.	Title	Key highlights	Anti-Tb compounds	Year	Publication number	Reference
1	A lysosome-targeted biological thiol fluorescent probe of pyrazoline derivatives and its preparation method, and application	The fluorescent probe enables real-time imaging of Cys, Hcy, and GSH levels in lysosomes of live cells. It offers high selectivity, sensitivity, rapid response, and low cytotoxicity, making it ideal for the simultaneous detection of these biomolecules	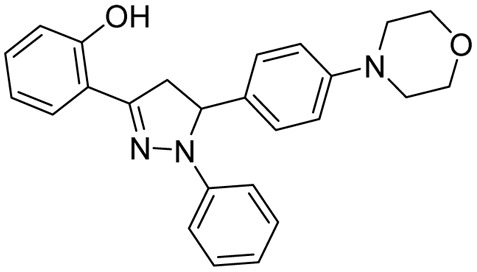	2024	CN118852115 (A)	153
2	Method for cyclization synthesis of pyrazoline from ketazine under the catalysis of hydrazinium salt	(i) High selectivity (99.6%) and yield (98.2%), (ii) catalyst reusable up to 5 times with good efficiency (≥95% selectivity, 75% yield), (iii) simple process, easy catalyst separation, and minimal waste, & (iv) scalable and environmentally friendly	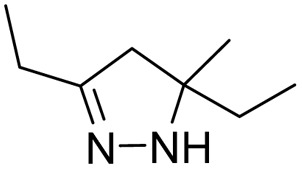	2023	CN115572263A	154
					CN115572263B	
3	A novel aryl azo pyrazole compound and its synthesis	This invention relates to a novel aryl azo pyrazoline compound which exhibits antimicrobial, anti-inflammatory, and antipyretic activities. It also discloses a novel process for the synthesis of the aryl azo pyrazoline	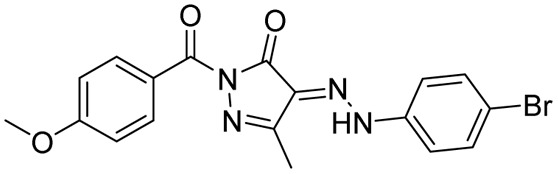	2023	WO2023209677	155
4	5-functionalized pyrazoline and preparation method thereof	(i) Novel compounds: 5-substituted pyrazoline derivatives with variable R_1_, R_2_, and R_3_ groups, (ii) key functional groups: hydroxymethyl, dimethylhydroxymethyl, and hydrazinoyl substitutions, (iii) synthesis route: derived from 1,3-diaryl-5-(3,5-dimethyl)pyrazoylpyrazoline precursors, (iv) efficient synthesis with yields ranging from 71% to 95%, (v) utilizes NaBH_4_, MeMgBr, and hydrazine hydrate under controlled temperatures (0 °C to RT), (vi) standard work-up with extraction and recrystallization (*e.g.*, hexane: ethyl acetate = 12 : 1)	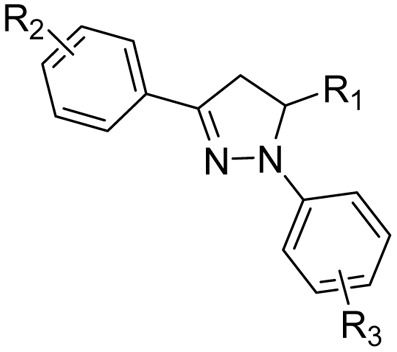	2022	CN115490638A	156
5	3-(2-Hydroxyphenyl)-5-phenyl-4,5-dihydro-1*H*-pyrazole derivatives, and method of preparing the same 3-(2-hydroxyphenyl)-5-phenyl-4,5-dihydro-1*H*-pyrazole derivatives, and method of preparing the same	Novel mycobactin-mimicking compounds show strong anti-Tb action and effectively block bacterial efflux mechanisms	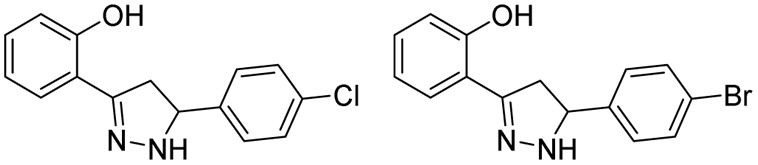	2022	Indian patent no. 455777	157
6	Pyrazoline thiazole derivative as well as preparation method and application thereof	(i) Pyrazoline–thiazole derivatives with variable R_1_ and R_2_ groups, (ii) three-step method *via* chalcone intermediates. Specific molar ratios and solvents; TLC-monitored reactions, & (iii) potential anti-inflammatory agents (effective in LPS-induced RAW264.7 cells)	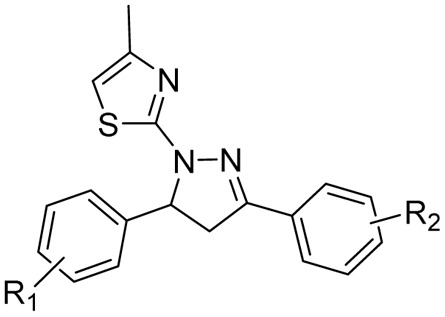	2021	CN113264928A	158
					CN113264928B	
7	Novel sulphonamide compound synthesis and uses thereof	(i) Carbonic anhydrases, particularly the β-CA isoforms (mtCA 1, mtCA 2, and mtCA 3) encoded by *Mtb* as a drug target, (ii) compounds showed the highest anti-tubercular activity against dormant (MIC: 2.12 μg mL^−1^ to 29.5 μg mL^−1^) and active phages (MIC: 2.48 μg mL^−1^ to 29.6 μg mL^−1^) of *Mtb* H37Ra in *in vitro* and *ex vivo* studies	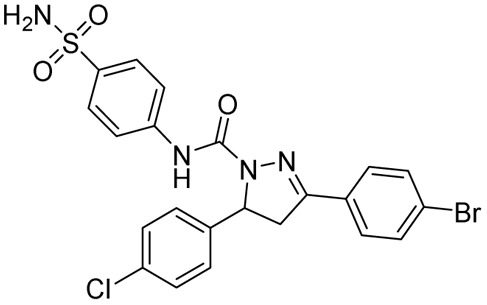	2015	IN1493/DEL/2015	159
8	Halogenated pyrazoline derivatives for the treatment of *Mtb*	Green methodologies, such as ultrasonic irradiation, have enabled higher yields (up to 83%), faster reaction times, and demonstrated potent anti-tubercular activity (MIC: 6.25 μg mL^−1^) of the synthesized compounds	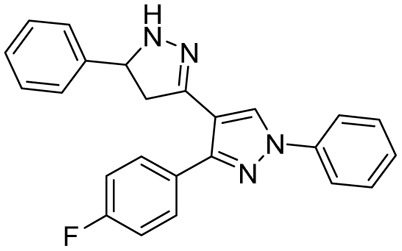	2012	Indian patent no. 126/MUM/2012	139
9	Pyrazoline derivatives for the treatment of tuberculosis	(i) Pyrazolone derivatives as inhibitors of the *Mtb* shikimate kinase (MtSK) enzyme, (ii) compounds were pharmaceutically acceptable salts or *in vivo* hydrolysable esters, (iii) synthesized using novel methods	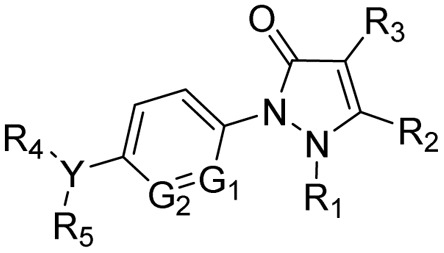	2010	US20100179161A1	160
					CA2619262A1	

## 2 Future perspectives and challenges

Pyrazoline derivatives show considerable promise in the fight against tuberculosis (TB) and other drug-resistant mycobacterial infections due to their potent activity and pleiotropic molecular mechanisms of action targeting a range of validated protein molecules. Ongoing advancements in medicinal chemistry and structural biology are expected to enable the rational design and optimisation of these compounds, leading to the development of more potent, less toxic, and selective antimycobacterial agents. By structurally tailoring these molecules to enhance their pharmacokinetic properties and bioavailability, their clinical effectiveness could be significantly improved. Additionally, the potential of pyrazoline derivatives as core scaffolds for designing hybrid molecules could expand their therapeutic applications.

However, despite this potential, several challenges remain. A key issue is the urgent need for robust preclinical and clinical studies to assess the target-specific interaction, efficacy, safety, tolerability, and potential side effects of these compounds. Moreover, there is a pressing need for the continual design and development of reliable, rapid *in vitro* whole-cell phenotypic assays to efficiently validate target essentiality^161^ and evaluate large compound libraries and molecular formulations^162^ in diverse physiological conditions, particularly for unique, differentially culturable mycobacterial pathogens. Improving and modifying current methodologies^163^ to better mimic the *ex vivo*/*in vivo* intracellular host environment would also help reduce reliance on animal models. Future research should focus on designing analogues that are highly specific to intended targets, thereby minimising off-target effects and reducing the risk of broad-spectrum resistance.

The development of narrow-spectrum agents and adjunct therapies, such as combining pyrazoline derivatives with existing antibiotics (first-line anti-TB drugs, bedaquiline and linezolid), efflux pump inhibitors, or biofilm disruptors, could significantly enhance treatment outcomes and help reverse the current trend in antimicrobial drug resistance. Additionally, investigating novel conditionally essential targets within the mycobactin megasynthase pathway should be considered, alongside the development of new therapeutic strategies.^164^

## 3 Concluding remarks

The pyrazoline ring has emerged as a promising structural framework for developing lead molecules due to its capacity for versatile chemical modifications and potential activity against mycobacterial strains. The medicinal chemistry of pyrazoline compounds has been, and continues to be, an active area of research, although it remains relatively less explored due to the limited availability of literature. This growing interest is driven by (a) their notable biological properties, particularly their potential as antimycobacterial agents, (b) their favorable physicochemical characteristics, which make them valuable leads in drug discovery and suitable for further optimization through active analogue approaches or other strategies; and (c) their ability to inhibit various enzymatic pathways critical to mycobacterial survival. This review highlights recent advancements in the chemical space of pyrazolines as antitubercular agents, tracing the drug discovery efforts since their inception. It covers key molecular modifications, such as rational substitutions and conjugations, designed to enhance overall potency. It also discusses important synthetic methodologies, biological evaluation techniques, and relevant patents filed in this domain. Given the significance of this scaffold as a potential source of novel antimycobacterial agents, the article aims to assist synthetic and medicinal chemists in designing more potent analogues.

## Conflicts of interest

There are no conflicts of interest to declare.

## Supplementary Material

RA-015-D5RA03759J-s001

## Data Availability

This article is a review and does not include any original experimental data. All data discussed and analyzed in the manuscript are available from the cited published literature. No new datasets were generated or analyzed during the preparation of this review. General synthetic schemes with concise description for all the pyrazolines discussed in the review. See DOI: https://doi.org/10.1039/d5ra03759j.
