# Supplementary material for: Advancements in the design and development of pyrazoline-based antimycobacterial agents: an update and future perspectives
Source: RSC Adv. 2025 Sep 1;15(38):31360–401. doi: 10.1039/d5ra03759j (PMC12401172; doi:10.1039/d5ra03759j)
Supplement: RA-015-D5RA03759J-s001 [file RA-015-D5RA03759J-s001.pdf]

Supplementary Information (SI) for RSC Advances.
This journal is © The Royal Society of Chemistry 2025

Advancements in the Design and Development of Pyrazoline-Based Antimycobacterial Agents: An Update and Future Perspectives

Gourav Rakshit[1, 2], Soumi Chakraborty[1], Sanjib Bhakta[2¶], Venkatesan Jayaprakash[1¶]

[1]Department of Pharmaceutical Sciences and Technology, Birla Institute of Technology, Mesra, Ranchi, 835215, India

[2]Mycobacteria Research Laboratory, Institute of Structural and Molecular Biology, School of Natural Sciences, Birkbeck, University of London, London WCIE 7HX, United Kingdom

Address for Correspondence: venkatesanj@bitmesra.ac.in and s.bhakta@bbk.ac.uk (¶equal contribution)

S1. General Synthetic Chemistry Procedures

This section highlights several notable examples from the existing literature that detail efficient synthetic routes yielding pyrazoline derivatives with promising antitubercular activity.

Arthur (1975) described the synthesis using diethyl acetylsuccinate and thiosemicarbazide(*1*).

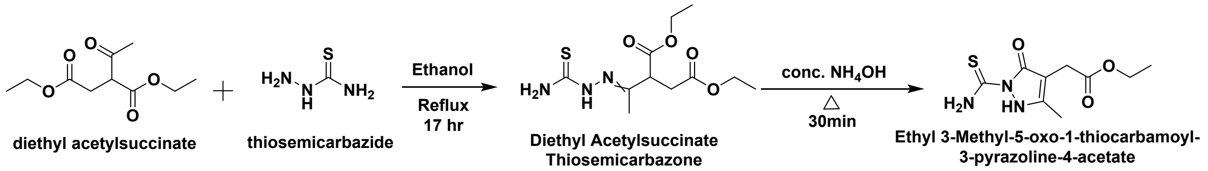

Küçükgüzel (2000) aromatic primary amines were diazotized and coupled with ethyl acetoacetate to produce ethyl 2-arylhydrazono-3-oxobutyrates. These were then reacted with substituted hydrazines in glacial acetic acid to yield 4-arylhydrazono-2-pyrazoline-5-one derivatives(*2*).

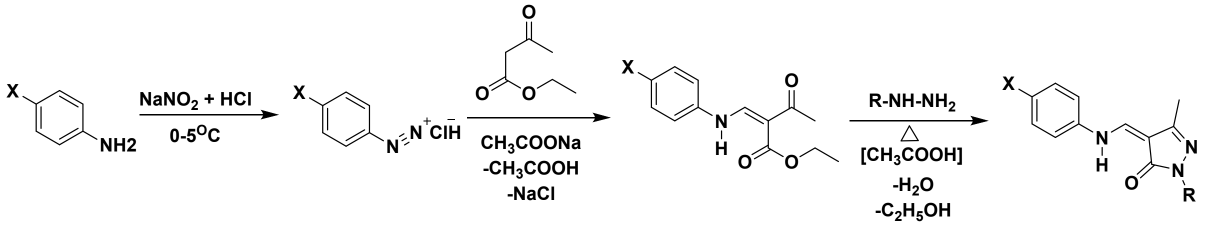

Zitouni (2005) synthesized pyrazoline derivatives by reaction of 1-(chloroacetyl)-3-(2-thienyl)-5-aryl-2-pyrazolines with appropriate sodium salts of N, N-disubstituted dithiocarbamoic acids, resulting in the formation of 1-[(N, N-disubstituted thiocarbamoylthio)acetyl]-3-(2-thienyl)-5-aryl-2-pyrazolines(*3*).

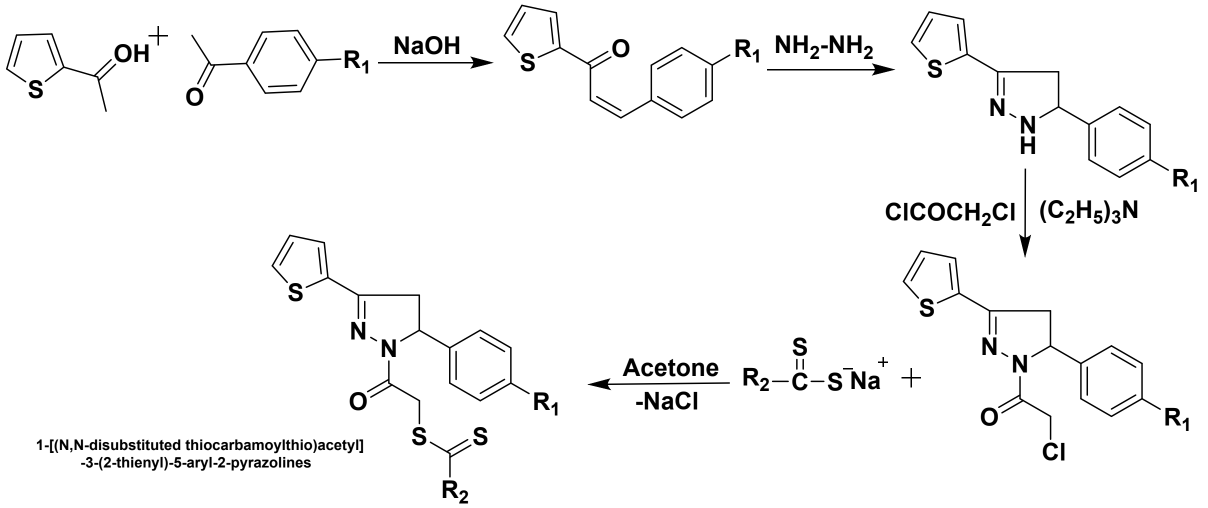

Shaharyar (2006) performed the synthesis of pyrazoline derivatives. Firstly, chalcones were synthesized by reacting 3-methyl-4-hydroxyacetophenone with the appropriate aldehyde in the presence of a base through the conventional Claisen-Schmidt condensation method. The

reaction between the chalcone and isonicotinyl hydrazide in an ethanolic solution, facilitated by glacial acetic acid, resulted in the formation of the pyrazolines, with reaction times varying from 8 to 14 hours(*4*).

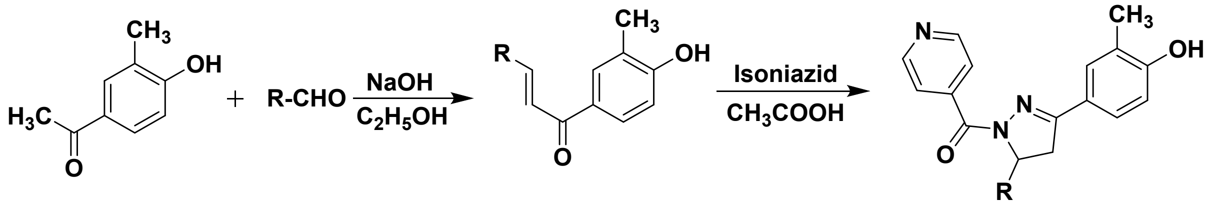

Shaharyar (2006) again synthesised pyrazoline derivatives, but this time the starting material was different. Initially, chalcones were synthesized by condensing 2-(4-formyl-2-methoxyphenoxy) acetic acid with various ketones in a dilute methanolic potassium hydroxide solution at room temperature using Claisen–Schmidt condensation. Subsequently, pyrazolines were synthesized by reacting these chalcones with appropriate aryl acid hydrazides in glacial acetic acid for 4 hours. He was successful in reducing the reaction time(*5*).

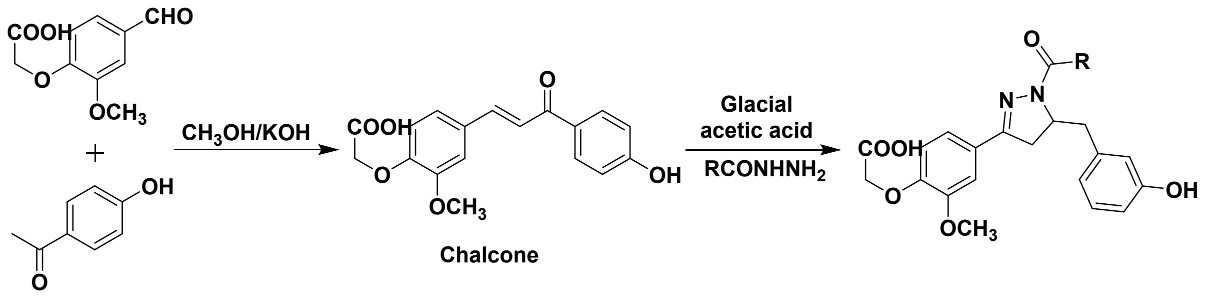

Following the same scheme, Mohamed Ashraf Ali in 2007 modified the reaction slightly to synthesize different pyrazolines with varied substitutions(*6*).

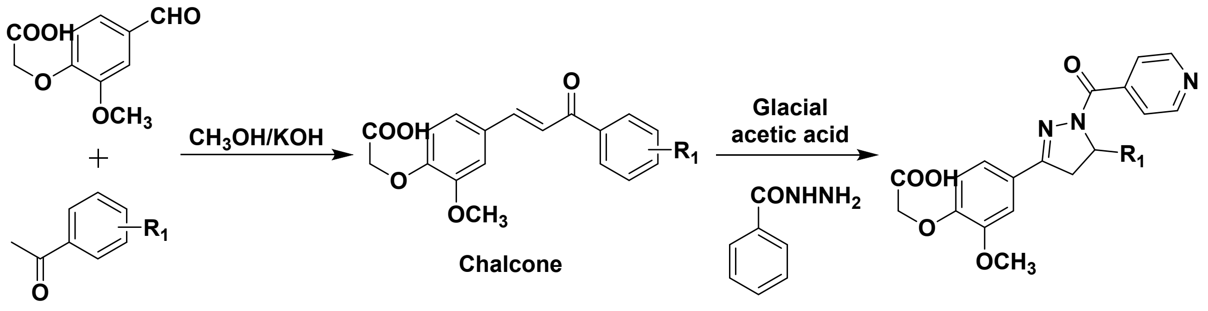

The same group in 2007 reported a modified method, where pyrazolines were synthesized by reacting hydrazine hydrate with chalcones, followed by condensation with appropriate aryl isothiocyanates, yielding N-substituted pyrazoline derivatives(*7*).

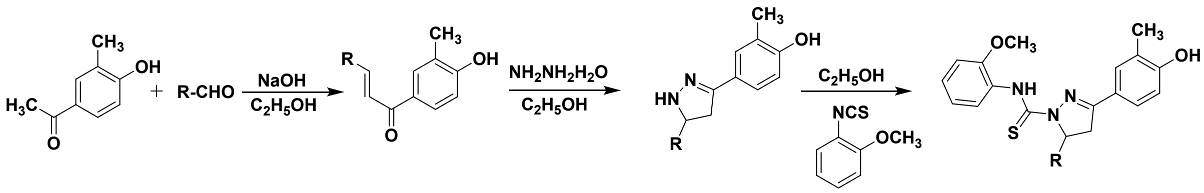

In 2008, they repeated the same scheme using 2-chloro isothiocyanates, resulting in the production of 2-chloroanilino-5- (substituted) phenyl -3-(4-hydroxy-3-methylphenyl)-4,5-dihydro-1H-1-pyrazolyl methanethiones(*8*).

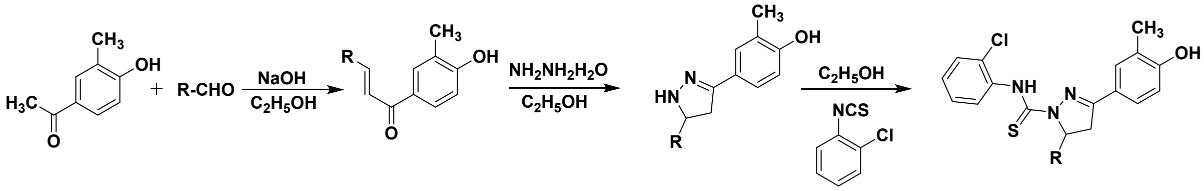

In 2010, Sivakumar and his team reported the synthetic methodology for 1,3,5-triphenyl-2-pyrazoline derivatives, starting from chalcones and then undergoing condensation with meta-chloroperoxybenzoic acid and phenylhydrazine(*9*).

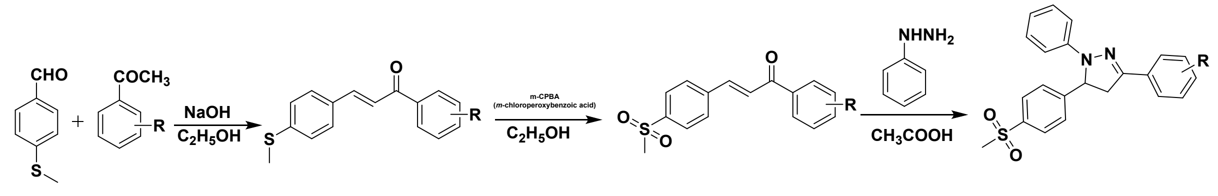

In 2010, Kasabe and Kasabe reported a synthetic methodology for substituted 3-pyrazoline derivatives. The reaction begins with the synthesis of pyridine-3-carbonyl hydrazine by refluxing nicotinamide and hydrazine hydrate in methanol. This is followed by a reaction with sodium nitrite and ethyl acetoacetate, then a condensation with sodium hydroxide, and finally, the addition of ethanethiohydrazide to achieve ring closure(*10*).

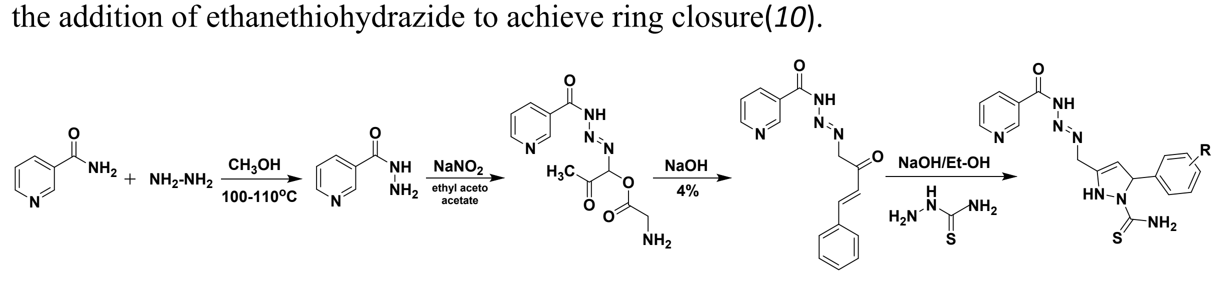

Sharma and group, in 2010, reported a complex synthetic methodology to synthesize substituted pyrazoline derivatives. Various intermediates were synthesised like ethyl-2-[2, 3-dichloroanilido] ethanoate, ethyl-2-[(N-benzoyl) 2, 3- dichloroanilido] ethanoate, ethyl-2-[(N-benzoyl) 2, 3-dichloroanilido] acetohydrazide, and N-cinnamoyl –N-2'-cyanoethyl -2, 3-dichloroaniline, which on further reaction yielded the pyrazolines(*11*).

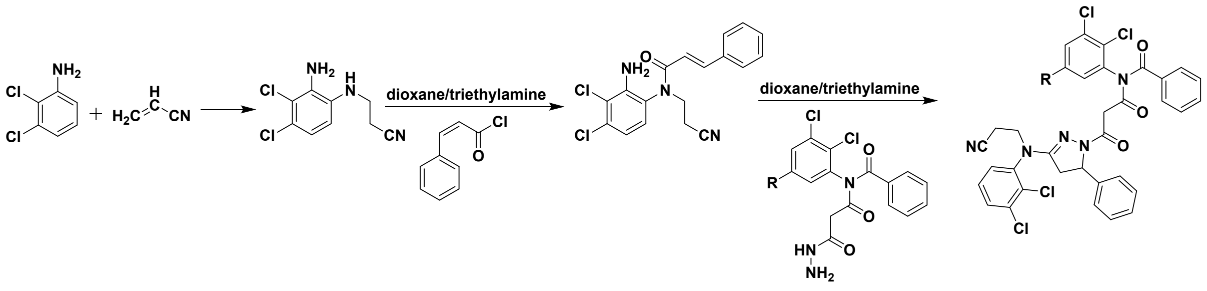

In 2010, Manna and Agrawal synthesized some pyrazoline derivatives using the microwave irradiation technique. This process of synthesis reduces reaction time and improves yield. First, crucial intermediates 1-benzo[b]furan-2-yl-3-phenyl-2-propen-1-ones (chalcones) were synthesized from 2-acetylbenzofuran and various aromatic aldehydes. These chalcones were then treated with isonicotinic acid hydrazide and nalidixic acid hydrazide to yield the final compounds: 3-benzofuran-5-aryl-1-pyrazolyl-pyridylmethanones and 3-benzofuran-5-aryl-1-pyrazolylcarbonyl-4-oxo-naphthyridins. Nalidixic acid hydrazide was prepared from the ester derivative of nalidixic acid(*12*).

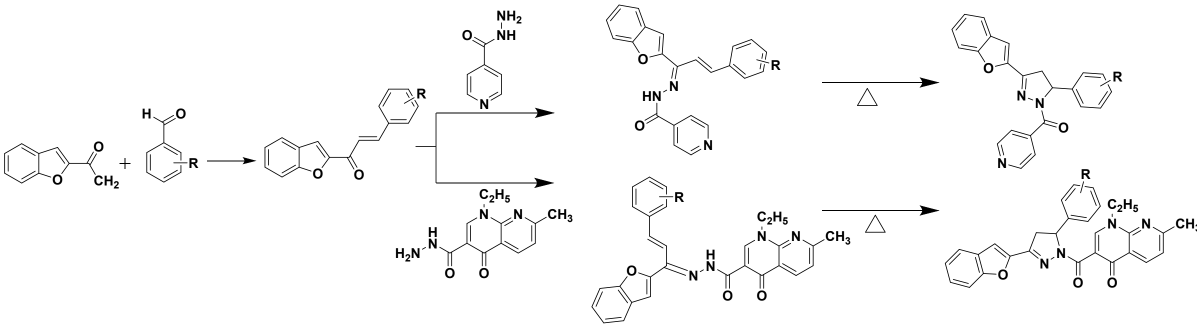

Taj and the group in 2011 performed a one-pot reaction to synthesise pyrazoline derivatives. The sydnone ring cleavage of 3-[4-(5-aryl-4,5-dihydro-1H-pyrazol-3-yl)]phenylsydnone (3a-k) in concentrated HCl and absolute alcohol at 75-80°C, followed by cyclization with cyclohexanone and refluxing at 150°C for 3 hours, led to the formation of 6,7,8,9-tetrahydro-2-(4,5-dihydro-5-aryl-1H-pyrazol-3-yl)-5H-carbazole (4a-k). This eco-friendly method is particularly interesting for producing pure amorphous compounds(*13*).

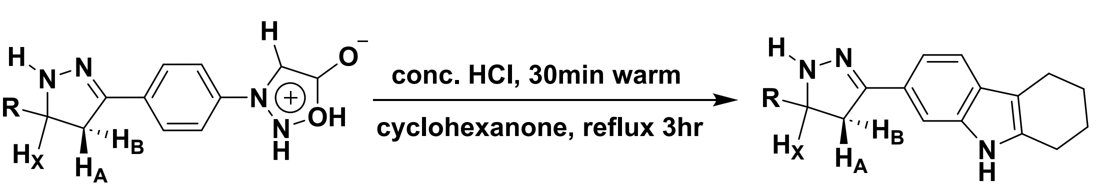

R = Phenyl, b ; R = o -chlorophenyl, c; R = m-chlorophenyl, d; R = p-chlorophenyl, e;
R = p□nitrophenyl,f ; R = o -hydroxyphenyl, g ; R = p -hydroxyphenyl, h ;
R = styryl, i ; R = methyl, j ; R = p-anisyl, k ; R = p -toly

In 2011, Ali and his research group reported the synthesis of twenty-one novel methanone derivatives containing a pyrazoline nucleus. Indanone was condensed with substituted aldehydes in methanol at room temperature, followed by reaction with acid hydrazide in the presence of glacial acetic acid, yielding methanone derivatives in 62–84% after ethanol recrystallization(*14*).

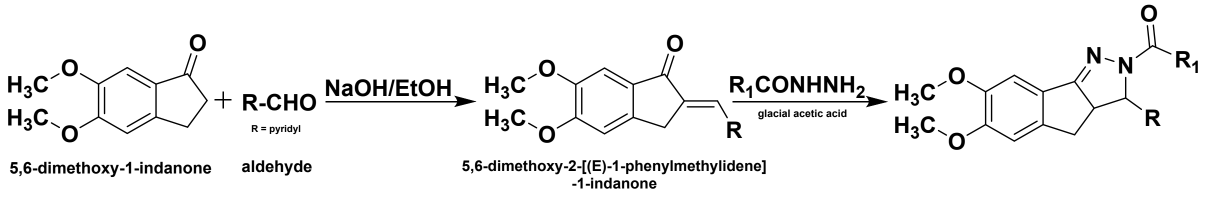

The same synthetic scheme above was employed by Ahsan in 2011 to synthesize eighteen novel 3-substituted-N-aryl-6,7-dimethoxy-3a,4-dihydro-3H-indeno[1,2-c]pyrazole-2-carboxamide analogues, based on the structure of the known antitubercular agent, thiacetazone(*15*).

In 2011, Manna and Agrawal synthesized 1,3,5-trisubstituted pyrazoline derivatives (having benzofuran and indophenazine). 2,5-Dihydroxybenzaldehyde was used to synthesize 5-hydroxy-2-acetylbenzofuran in the presence of chloroacetone and K_2CO_3. Intermediates were synthesised using substituted benzofuran and various aromatic aldehydes in a strong alkaline medium. Final products were synthesized from benzofuran chalcones in the presence of glacial acetic acid(*16*).

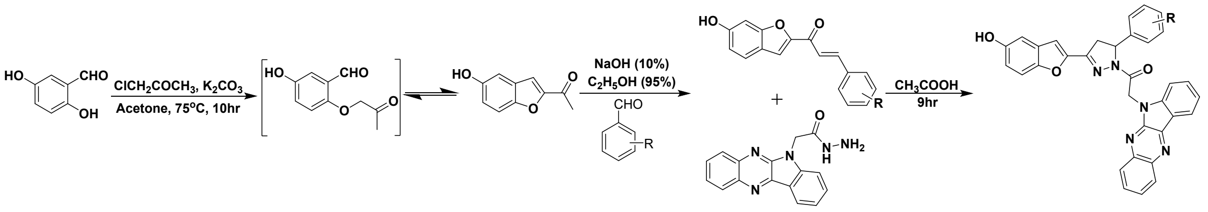

Shelke and his group in 2012 synthesised fluorinated pyrazolines using ultrasonic irradiation. Ultrasonic irradiation presents an environmentally friendly alternative to conventional methods for synthesizing pyrazoline derivatives. This technique is advantageous due to its simplicity, lower temperature requirements (room temperature), reduced reaction times (10–25 min), and higher yields (up to 83%)(*17*).

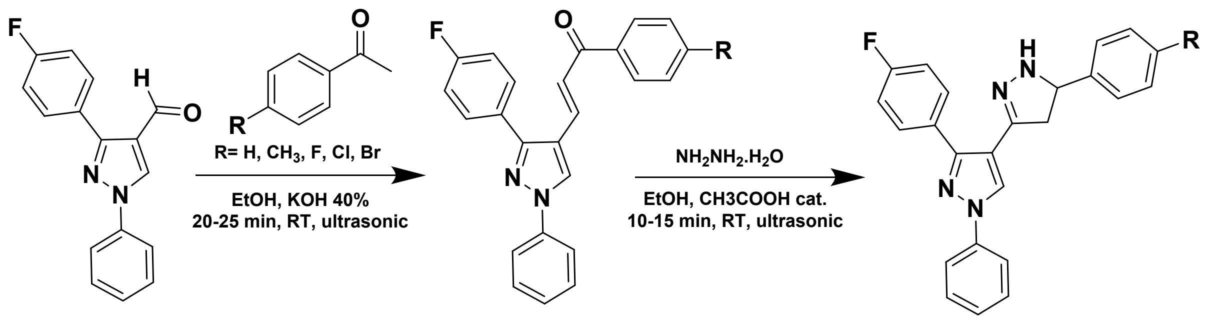

In 2012, Hazra and co-workers reported the synthesis of fluoro and nitro-substituted benzothiazolopyrazolines. This technique produced good yields(*18*).

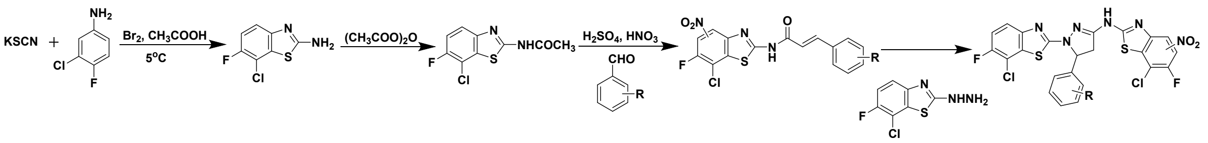

In 2012, Dinesh and the team reported the synthesis of substituted pyrazolines. Chalcones were synthesized by reacting 2,4-dihydroxyacetophenone with the substituted benzaldehyde in the presence of a base through the conventional Claisen-Schmidt condensation method. The reaction between the chalcone and hydrazine hydrazide in an ethanolic solution resulted in the formation of the pyrazolines, with reaction times varying from 5 to 6 hours(*19*).

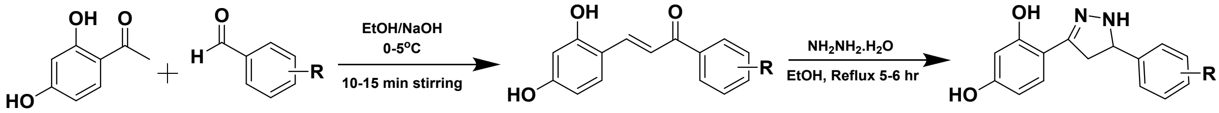

In 2014, Narendrasinh and the group reported the synthesis of benzoxazole-based pyrazoline derivatives. In the first step, the conventional Claisen-Schmidt condensation method synthesised chalcones in good yields (75-90%). The reaction between the chalcone and hydrazine hydrazide in an ethanolic solution resulted in the formation of the pyrazolines, with reaction times varying from 18-24 hours. Furthermore, derivatisation was carried out by using sodium dithionite, cyanogen bromide, and sodium bicarbonate, using methanol-water as a solvent(*20*).

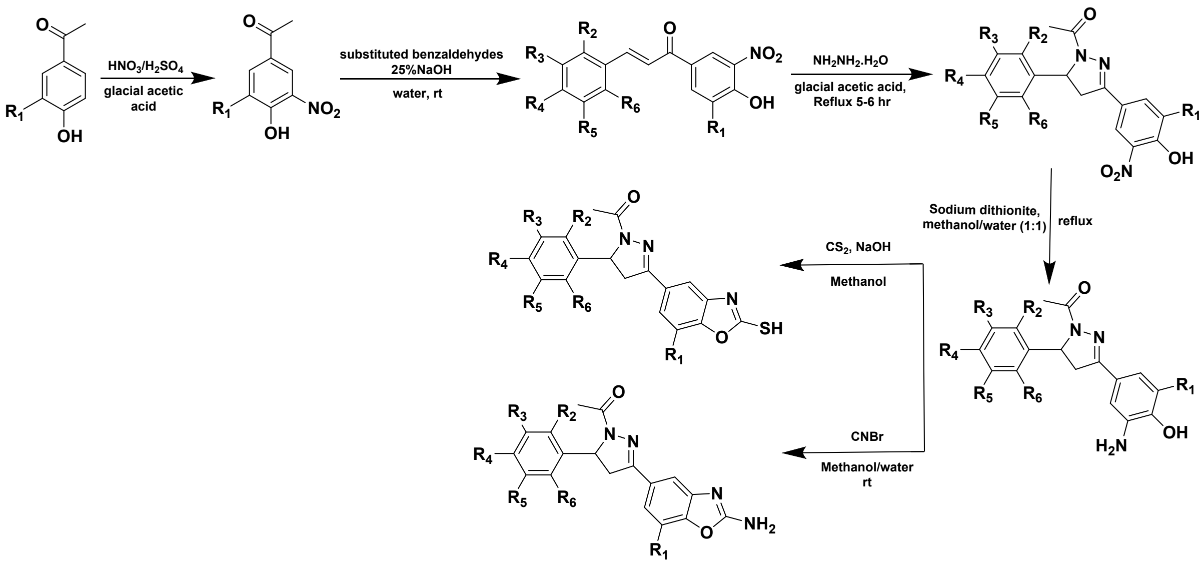

In 2014, Ahmad and his team synthesized sixteen new pyrazoline analogs derived from chalcones, which were prepared from *p*-acetamidophenol (paracetamol). The intermediate, 3-acetyl-4-hydroxyphenyl acetamide, was obtained by reacting *N*-(4-hydroxyphenyl)acetamide with acetic anhydride in dry pyridine, followed by treatment with anhydrous $AlCl_3$. Eight chalcones were then synthesized via Claisen–Schmidt condensation of this intermediate with various aromatic aldehydes in an alkaline medium. Their formation was confirmed by the appearance of a red color with concentrated H_2SO_4(*21*).

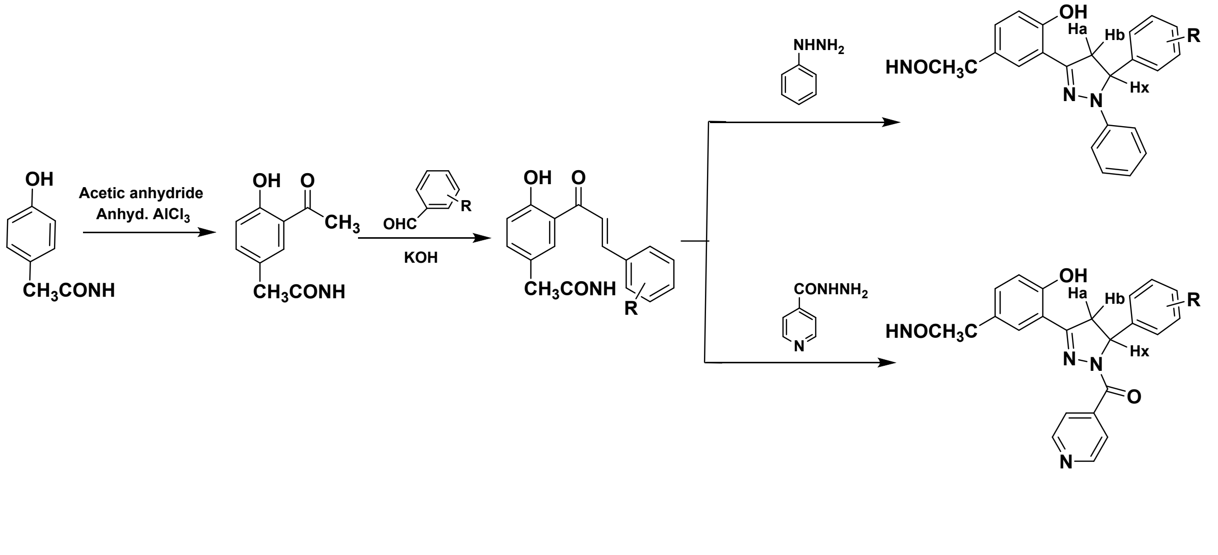

In 2014, Rana and his team synthesized 5-(1-acetyl-5-aryl-4,5-dihydro-3-pyrazolyl)-2-substituted benzoxazoles via a multistep process starting from substituted acetophenones. Nitration using fuming nitric acid in glacial acetic acid yielded nitro derivatives, which underwent aldol condensation with various benzaldehydes to form 3-aryl-1-(4-nitro-3-hydroxyphenyl)prop-2-en-1-ones. Cyclization with hydrazine hydrate produced 5-(1-acetyl-5-

aryl-4,5-dihydro-3-pyrazolyl)-2-nitrophenols. These were reduced with sodium dithionite to their amino counterparts. Final cyclization of o-aminophenol derivatives was performed via: (a) treatment with CS_2/KOH to yield 2-mercaptobenzoxazoles, or (b) reaction with cyanogen bromide in THF-water to obtain 2-aminobenzoxazoles(22).

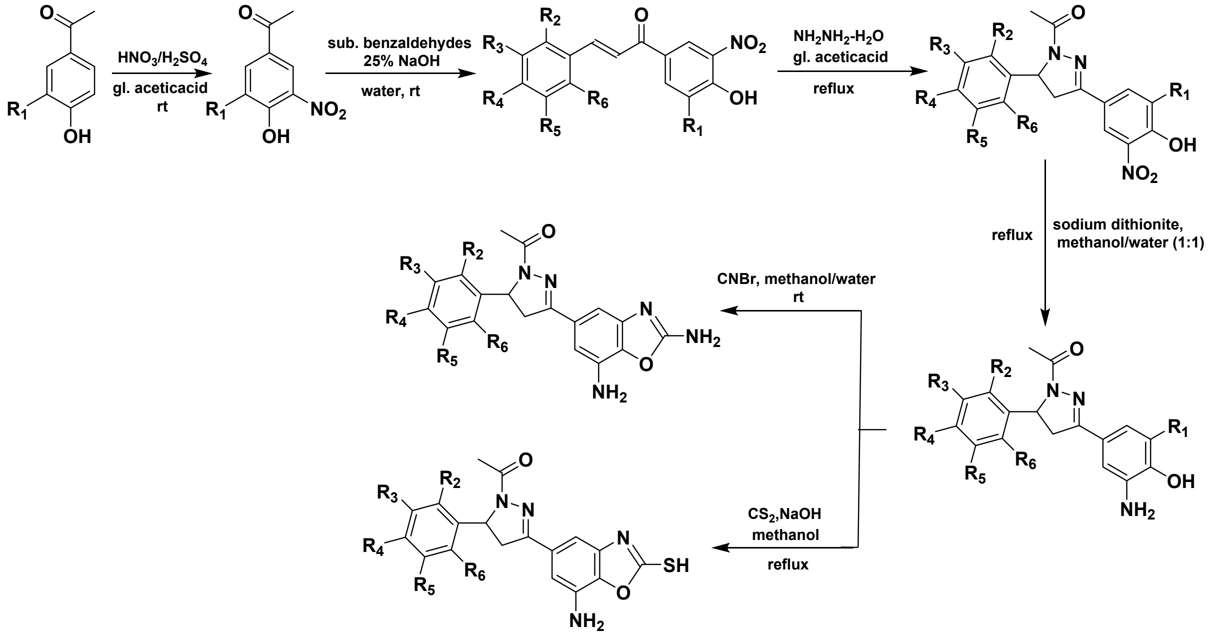

In 2014, Kalaria and his team reported the synthesis of novel compounds starting from 5-chloro-3-methyl-1-phenyl-1H-pyrazole-4-carbaldehyde, prepared via the Vilsmeier–Haack reaction of 3-methyl-1-phenyl-1H-pyrazol-5(4H)-one. Nucleophilic substitution of the C5-chloro group with imidazole in refluxing DMF using anhydrous K_2CO_3 yielded 5-(1H-imidazol-1-yl)-3-methyl-1-phenyl-1H-pyrazole-4-carbaldehydes. These aldehydes underwent Claisen–Schmidt condensation with substituted acetophenones in ethanolic NaOH at room temperature to form pyrazolic chalcones. Subsequent treatment with hydrazine hydrate or hydroxylamine hydrochloride in ethanol with catalytic acetic acid produced pyrazoline and isoxazoline derivatives(23).

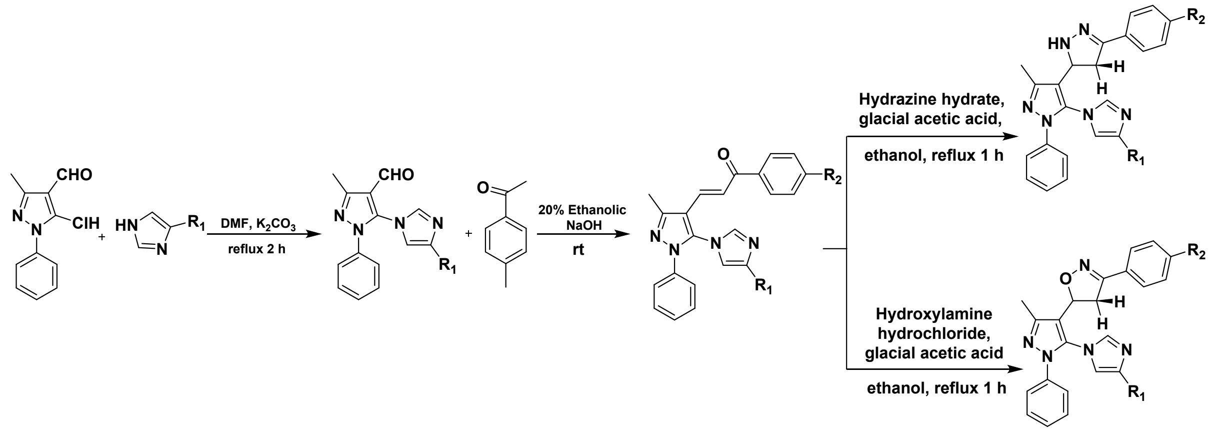

In 2014, Monga and his team synthesized various derivatives starting from 3-nitroacetophenone and aromatic aldehydes via condensation to form 1,3-diaryl-2-propen-1-one (chalcones). Cyclization of these chalcones with hydrazine hydrate in glacial acetic acid yielded N-acetyl pyrazolines, while the same reaction in absolute ethanol produced pyrazoline derivatives. Additionally, reaction with ethyl acetoacetate in the presence of barium hydroxide gave cyclohexanone derivatives(*24*).

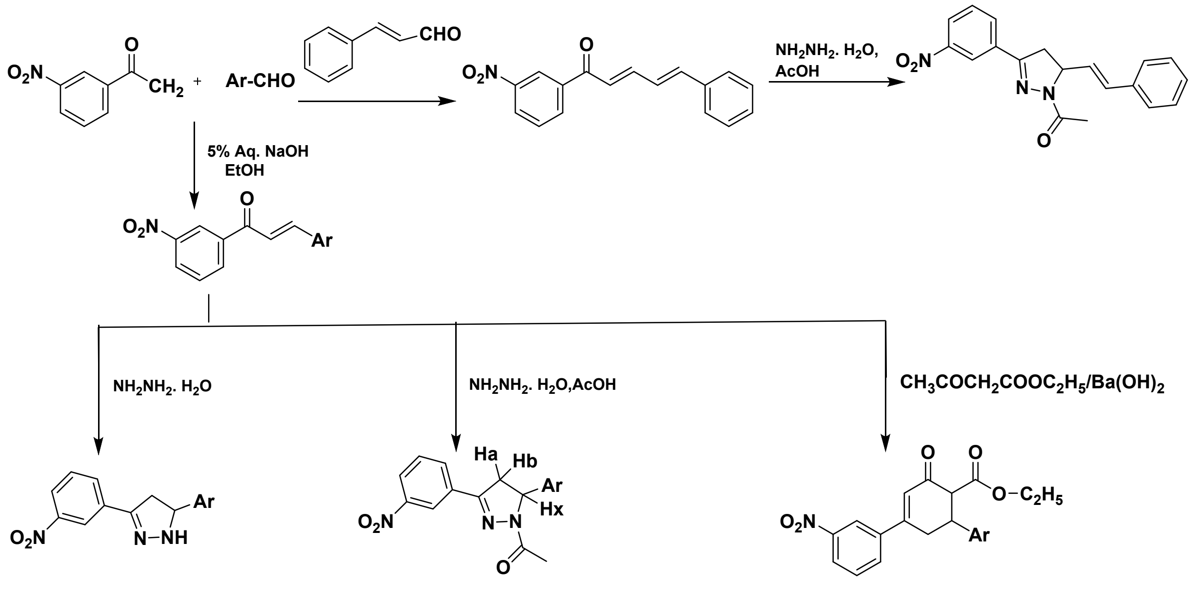

In 2014, Karad and his team synthesized a series of pyrazolylpyrazolines. Starting from 3-methyl-1-phenyl-1H-pyrazol-5(4H)-one, they prepared 5-chloro and subsequently 5-aryloxy pyrazole-4-carbaldehydes. These underwent Claisen–Schmidt condensation with 4-fluoroacetophenone to form chalcones, which were then cyclized with substituted phenylhydrazine hydrochlorides under microwave irradiation to yield the final pyrazolylpyrazolines(*25*).

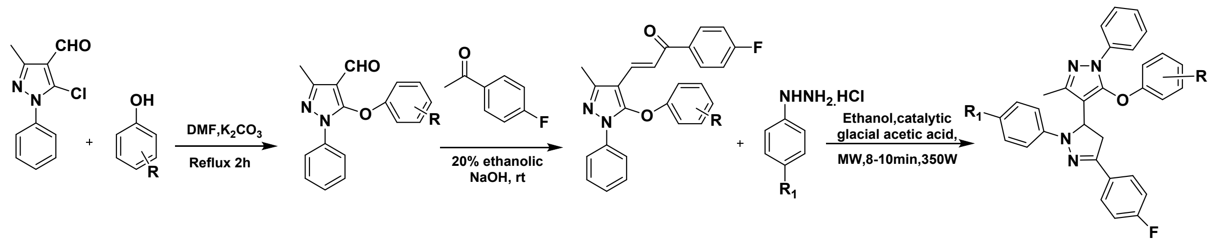

In 2015, Deshpande and his team synthesized chalcones by reacting substituted acetophenones with piperonal in ethanol using 50% NaOH at 10–15 °C, followed by overnight stirring. The product was precipitated with ice, neutralized if needed, filtered, washed, and recrystallized. These chalcones were then refluxed with hydrazine hydrate or phenylhydrazine in glacial acetic acid for 8 hours. After cooling, the mixture was distilled, poured into ice water, neutralized with sodium bicarbonate, and the final product was filtered and recrystallized(*26*).

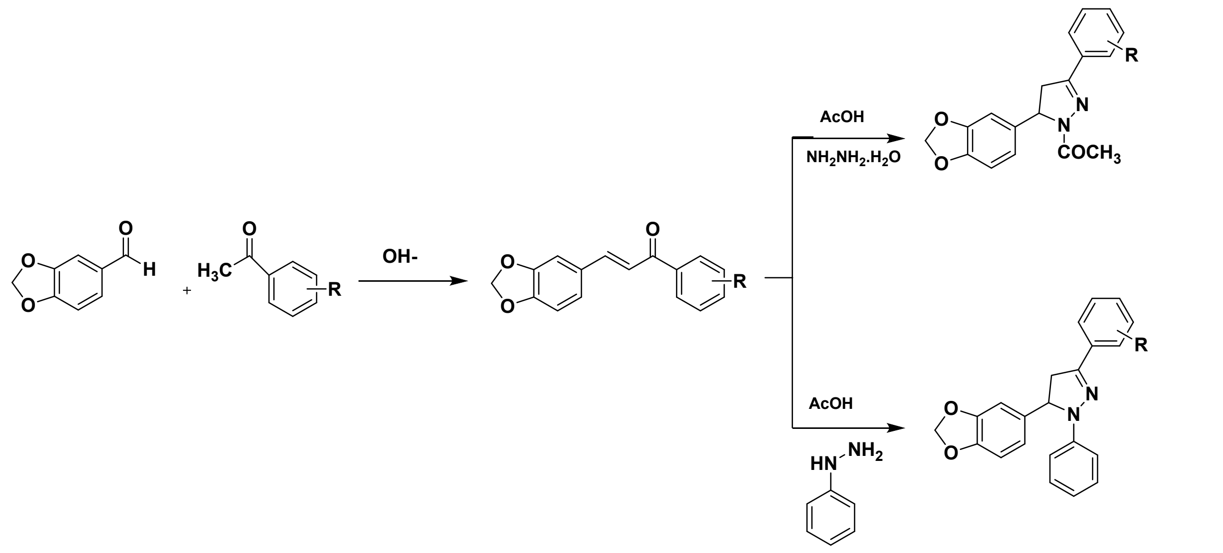

In 2015, Napoleon and his team synthesized bisbenzylidene cycloalkanones by grinding cyclohexanone, aldehydes, and solid NaOH, followed by acid treatment and purification. These intermediates were then reacted with isoniazid in ethanol using p-toluenesulfonic acid as a catalyst under reflux. After completion, the product was isolated, washed, and recrystallized to yield the final compounds(*27*).

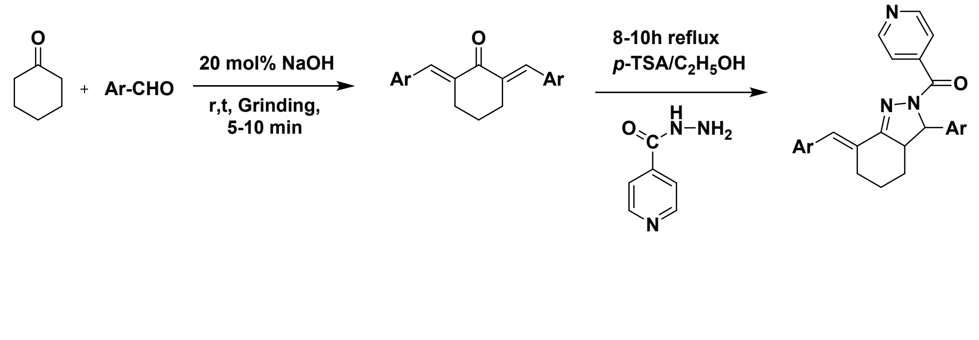

In 2016, Joshi and his team synthesized (4-pyrrol-1-yl)acetophenone via the Paal-Knorr reaction from 4-aminoacetophenone and 2,5-dimethoxytetrahydrofuran. Chalcones were formed through Claisen–Schmidt condensation with substituted aldehydes in ethanolic NaOH. These were cyclized with hydrazine hydrate and glacial acetic acid under solvent-free conditions to yield N-acetyl pyrazolines. Further reaction with hydroxylamine hydrochloride and sodium acetate in glacial acetic acid afforded 5-(4-(1H-pyrrol-1-yl)phenyl)-3-substituted phenylisoxazoles(*28*).

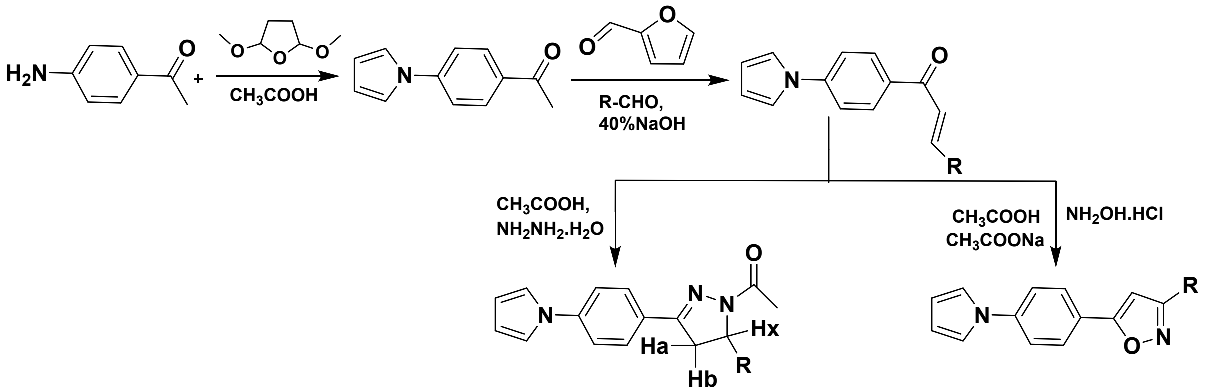

In 2016, Muneera, and their team synthesized bioactive pyrazoline derivatives via base-catalyzed Claisen–Schmidt condensation of imidazole-2-carboxaldehyde with 1-acetyl-2-hydroxynaphthalene, followed by cyclization with phenylhydrazine (L1), 2,3-dimethylphenylhydrazine (L2), or 3-nitrophenylhydrazine (L3)(*29*).

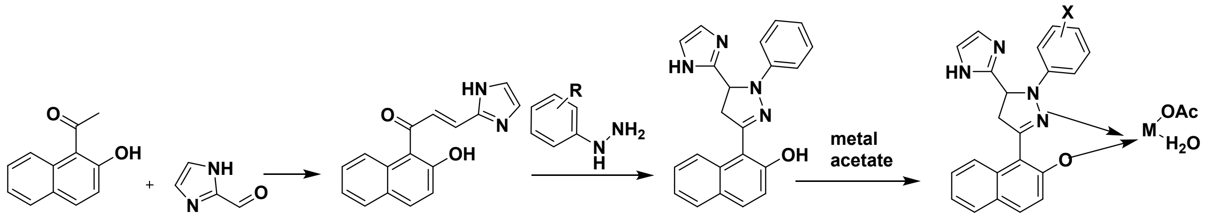

In 2016, Karad and his team synthesized a novel series of pyrazoline scaffolds. Starting from 2-chloroquinoline-3-carbaldehyde (prepared via the Vilsmeier–Haack reaction), 2-morpholinoquinoline-3-carbaldehyde was obtained by refluxing with morpholine in DMF using anhydrous K_2CO_3. This intermediate underwent Claisen–Schmidt condensation with 4-substituted acetophenones to form [(2-morpholinoquinolin-3-yl)]chalcones. These chalcones

were then cyclized with various hydrazine derivatives under different conditions, including microwave irradiation, to yield the desired pyrazoline derivatives(*30*).

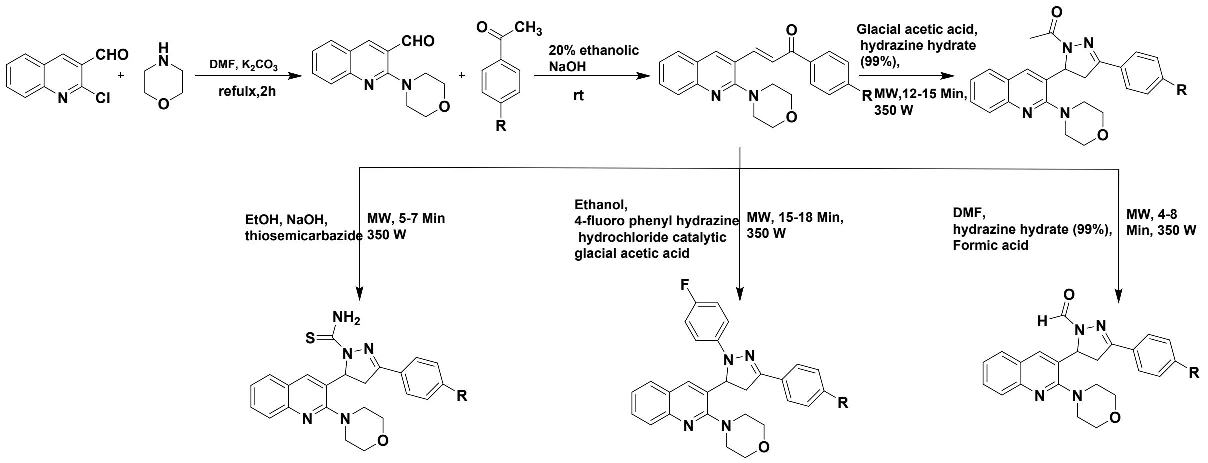

In 2016, Anjani and her team synthesized three combinatorial libraries of substituted phenyl derivatives, pyrazoline, isoxazole, and benzodiazepine, by reacting chalcones with phenylhydrazine hydrochloride, hydroxylamine hydrochloride, and o-phenylenediamine, respectively(*31*).

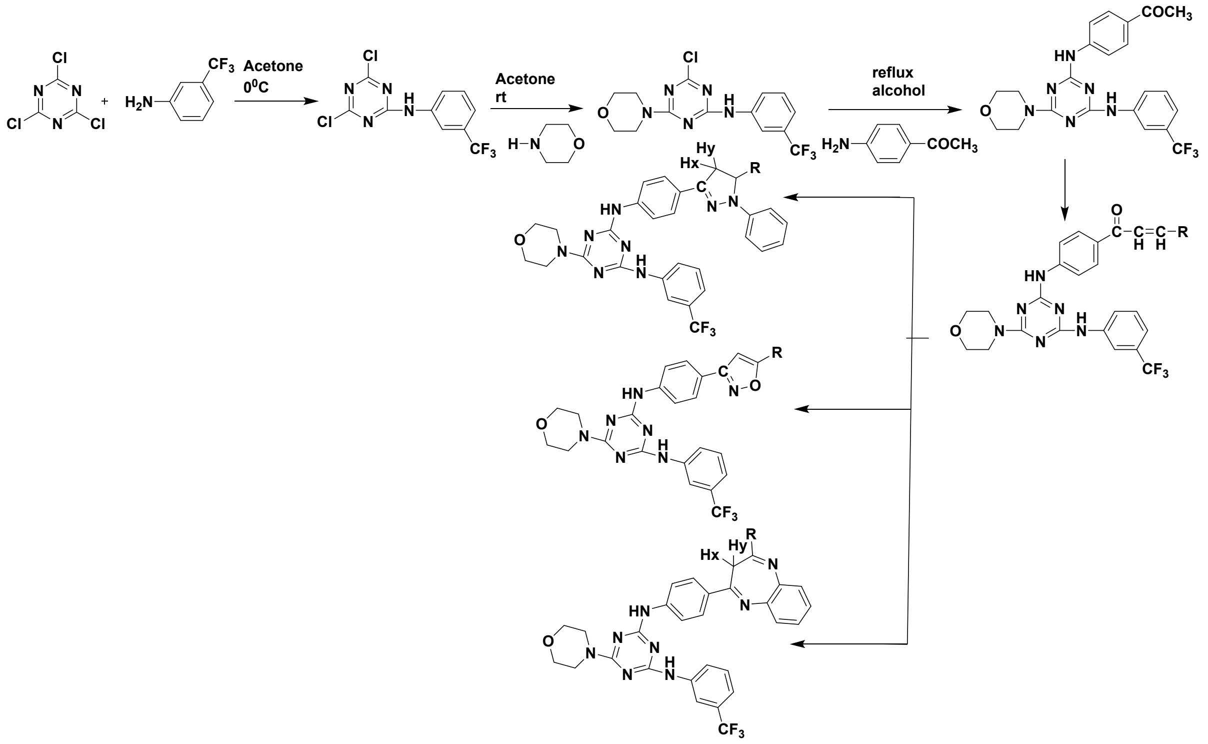

In 2017, Dixit and his team synthesized (4-pyrrol-1-yl)acetophenone via Paal-Knorr reaction using 4-aminoacetophenone and 2,5-dimethoxytetrahydrofuran in glacial acetic acid. Chalcones were then prepared through Claisen–Schmidt condensation of this compound with

substituted aldehydes in 40% alcoholic NaOH. These chalcones were subsequently cyclized with hydrazine hydrate and formic acid under solvent-free conditions to yield pyrazolyl carbaldehydes(*32*).

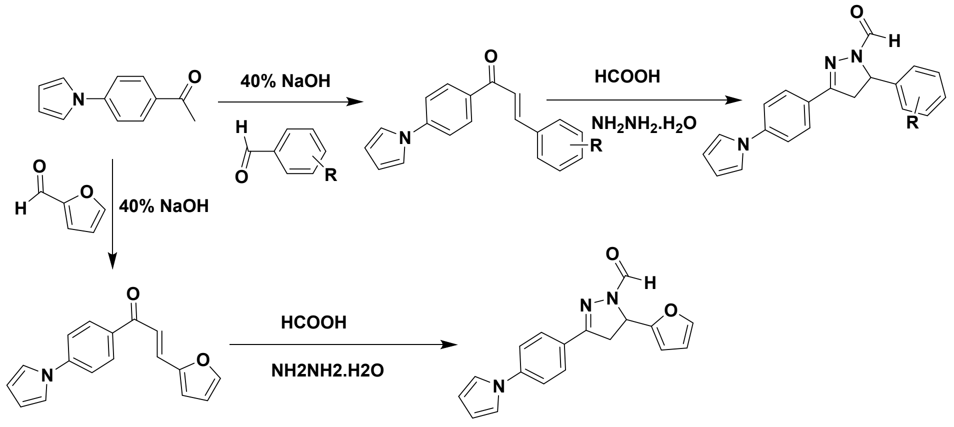

In 2017, Sadashiva and his team synthesized pyrazoline derivatives integrated with a sulfonamide scaffold. Substituted chalcones were first prepared via Claisen–Schmidt condensation using para-substituted aryl aldehydes and acetophenones. These chalcones were then reacted with methyl hydrazinecarboxylate in methanol to form N-methyl ester-substituted pyrazolines. Further treatment with hydrazine hydrate yielded the corresponding carbohydrazides. Finally, reaction with fluorinated benzene sulfonyl chloride in the presence of pyridine produced the target sulfonamide-linked pyrazoline derivatives(*33*).

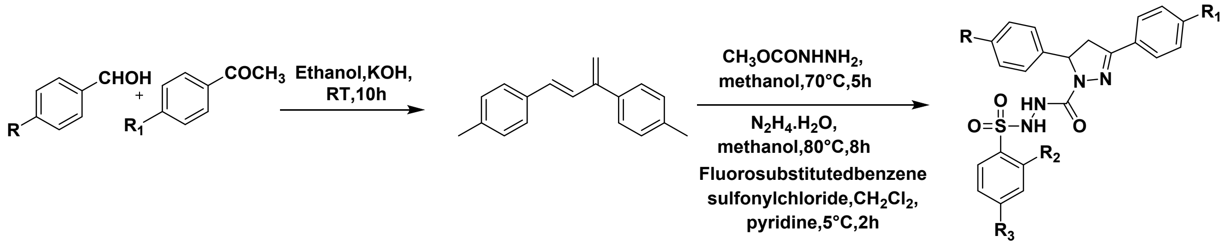

In 2017, Sowmya and team synthesized 6-aryl-pyridine-based 4,5-dihydro-2-pyrazolines. Starting from acetophenones and enaminones, they performed a one-pot cyclocondensation to obtain pyridine intermediates. These were converted to chalcones via Claisen–Schmidt condensation and then cyclized with hydrazine hydrate to form the final pyrazoline derivatives(*34*).

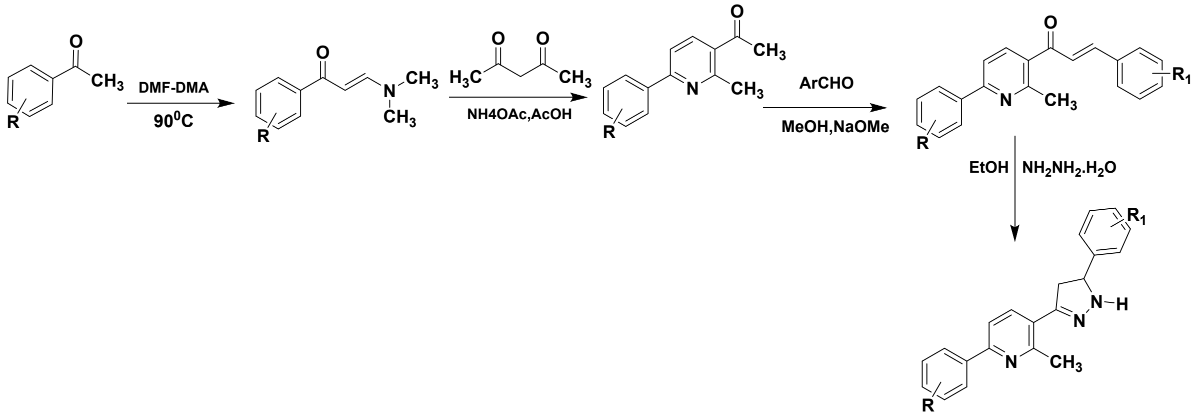

In 2017, Hallikeri and his team synthesized pyrrole-based biheterocyclic derivatives. Substituted chalcones were first prepared from 4-(1H-pyrrol-1-yl)acetophenone and substituted benzaldehydes using 40% NaOH in ethanol. These chalcones were then cyclized with phenylhydrazine to yield pyrazoline derivatives(*35*).

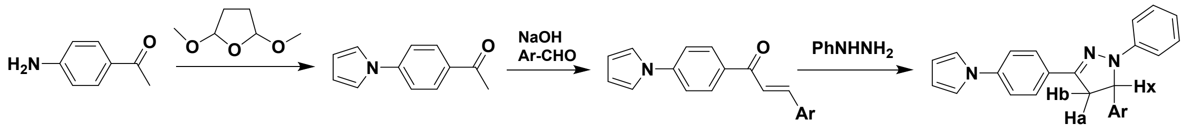

In 2018, Thakor and team synthesized chalcones by reacting 2-acetylthiophene with various aldehydes in methanolic KOH at room temperature for 2 hours. The products were precipitated with ice water, neutralized with dilute HCl, filtered, and recrystallized from methanol. These enones were then reacted with phenylhydrazine and methanolic potassium tert-butoxide under reflux for 5–6 hours to yield pyrazolines. The resulting ligands were further complexed with Na_2PdCl_4 in chloroform/methanol under reflux and stirred for 48 hours to obtain a greenish-brown Pd(II) complex(*36*).

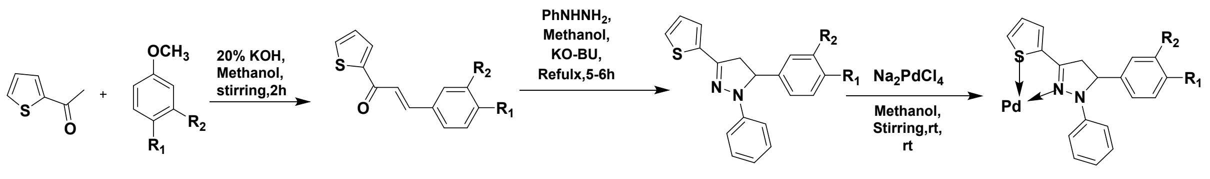

In 2019, Lokesh and his team synthesized 2-pyrazoline derivatives by reacting 2,5-dichloro-3-acetylthiophene chalcones with isoniazid in ethanol–pyridine at room temperature. Products were purified by chromatography and recrystallized from chloroform(*37*).

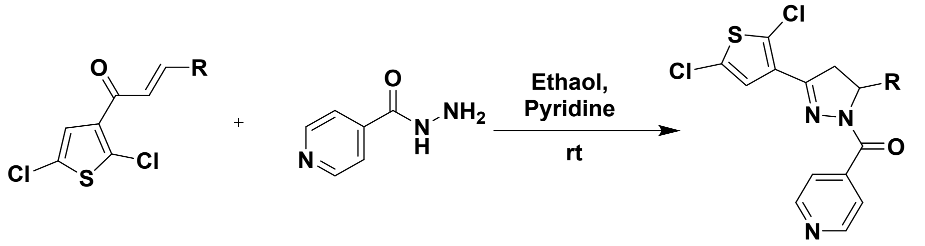

In the year 2020, Pola and his team synthesized a new series of naphthyl chalcones and their pyrazoline derivatives using substituted acetophenones, substituted naphthaldehydes, and hydrazine hydrate as starting materials(*38*).

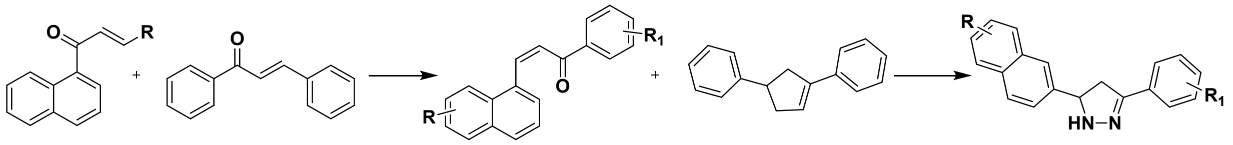

In 2021, Wong and his team synthesized chalcone derivatives by refluxing acetophenone with substituted benzaldehydes in methanol using piperidine as a catalyst. These chalcones were cyclo-condensed with 4-phenyl-3-thiosemicarbazide or 4-hydroxybenzhydrazide in ethanol with NaOH for 3–8 hours. After reaction completion (monitored by TLC), the mixtures were cooled, neutralized, and precipitated with ice. Products were filtered, washed, recrystallized from ethanol, and purified by column chromatography(*39*).

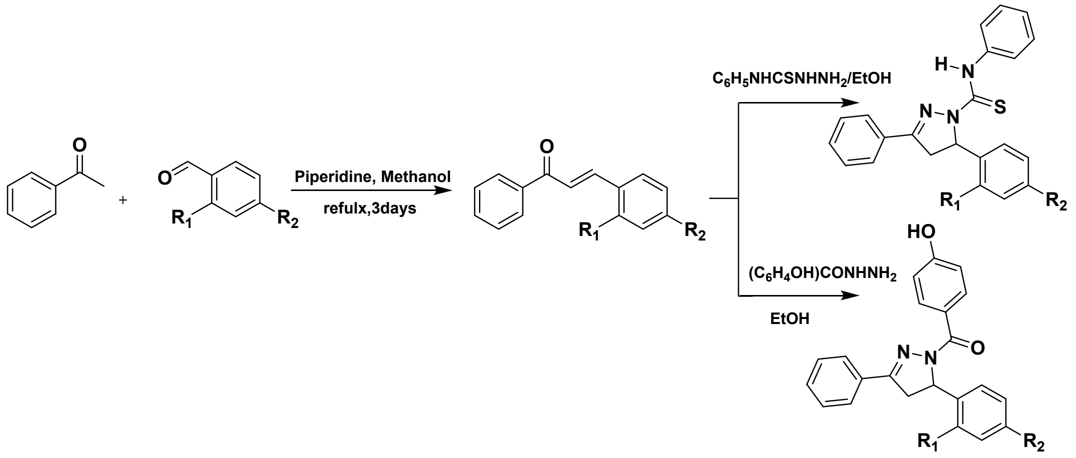

In 2024, Rasgania and her team synthesized thiophene chalcones by reacting 2-acetylthiophene with aromatic aldehydes in methanol and KOH at 0–5 °C, followed by stirring for 4 h and overnight standing. The product was isolated, neutralized, and recrystallized from ethanol. These chalcones were then refluxed with isoniazid in acetic acid for 24 h. After neutralization and purification, the final compounds were obtained in good yield(*40*).

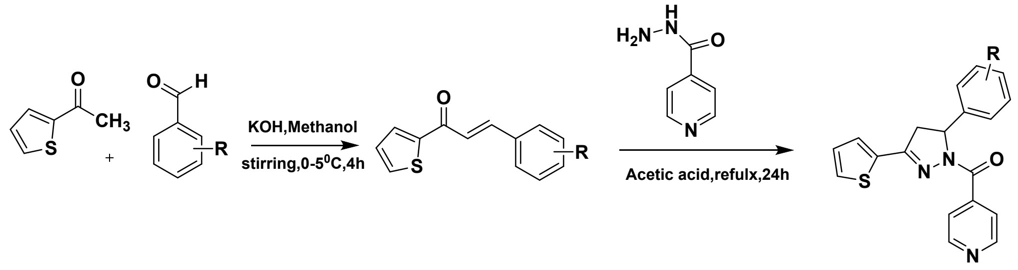

In 2025, Zala's team synthesized 4-hydrazinylbenzenesulfonamide from sulfanilamide via diazotization and $SnCl_2$/HCl reduction. 7-Chloro-4-hydrazinylquinoline was obtained by refluxing 4,7-dichloroquinoline with hydrazine hydrate in ethanol. Intermediates were formed by reacting 2-acetylthiophene or 2-acetylfuran using $POCl_3$ in DMF at 75–80 °C. These were converted to chalcones using substituted acetophenones in methanol/NaOH at room temperature. Final compounds were synthesized by reacting chalcones in ethanol and HCl at 75 °C, then neutralized, filtered, and crystallized, yielding 78–86% with high purity(*41*).

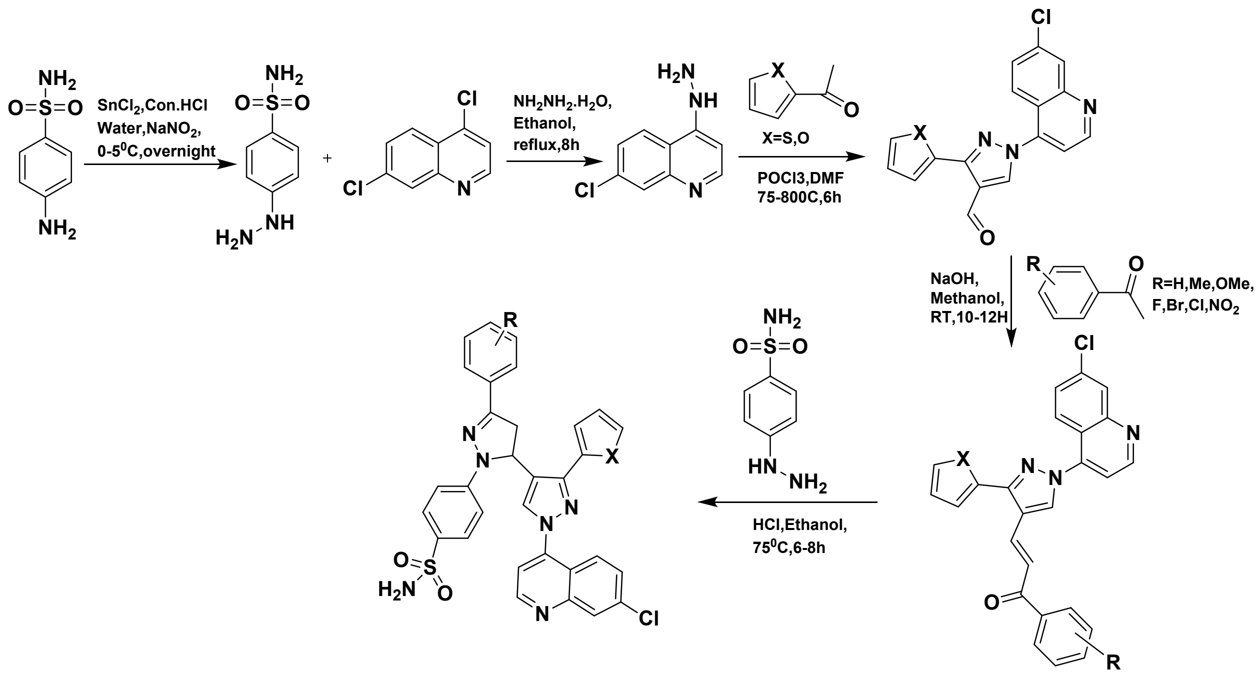

In 2025, Tailor reported the synthesis of heterocyclic derivatives from chalcones. 1-Acetylpyrazolines were synthesized by refluxing chalcones with hydrazine hydrate and 5 mL glacial acetic acid in alcohol for 6–8 h. The product was precipitated in ice and neutralized with

Na_2CO_3(*42*).

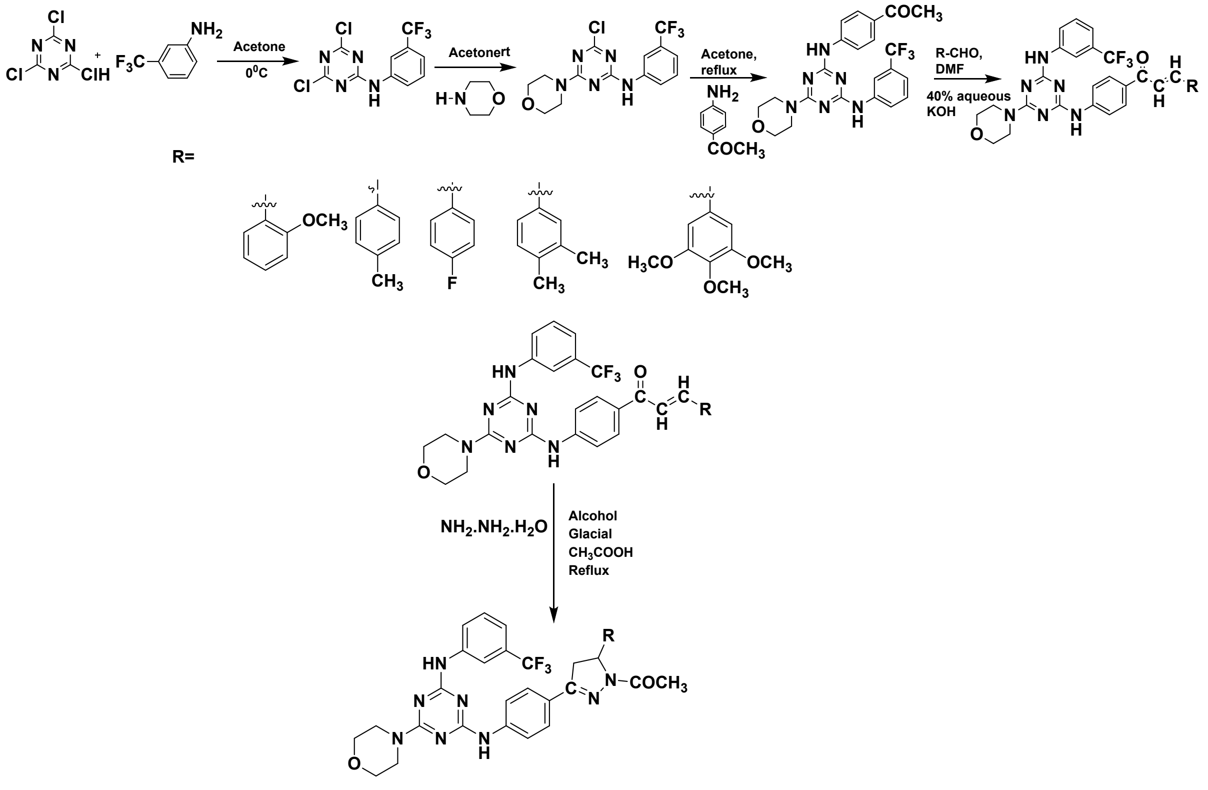

Cui (2025) reported the synthesis of 2-pyrazoline derivatives. The key step involved iodine-mediated intramolecular 5-exo-trig cyclization of homoallyl hydrazines in the presence of iodine (3 equiv.) and $NaHCO_3$ (5 equiv.) at room temperature, selectively forming 2-pyrazoline derivatives. No side products (e.g., azetidines, pyrrolidines) were observed(*43*).

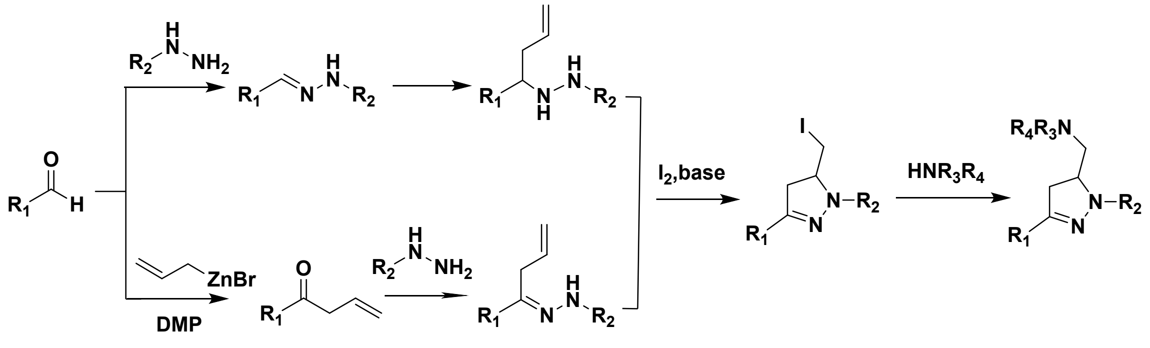

References

1. A. A. Santilli, D. H. Kim, F. J. Gregory, *J Pharm Sci.* **64**, 1057–1058 (1975).
2. Ş. G. Küçükgüzel *et al., Eur J Med Chem.* **35**, 761–771 (2000).
3. A. Özdemir, G. T. Zitouni, Z. A. Kaplancikli, *Turk J Chem.* **32**, 529–538 (2008).
4. M. Shaharyar, A. A. Siddiqui, M. A. Ali, D. Sriram, P. Yogeeswari, *Bioorg Med Chem Lett.* **16**, 3947–3949 (2006).

5. A. A. Siddiqui, M. A. Ali, *Bioorg Med Chem Lett*. **16**, 4571–4574 (2006).

6. M. A. Ali, M. Shaharyar, A. A. Siddiqui, *Eur J Med Chem*. **42**, 268–275 (2007).

7. M. A. Ali, M. S. Yar, M. Kumar, G. S. Pandian, *Nat Prod Res*. **21**, 575–579 (2007).

8. M. A. Ali, M. S. Yar, *J Enzyme Inhib Med Chem*. **22**, 183–189 (2007).

9. P. M. Sivakumar, S. P. Seenivasan, V. Kumar, M. Doble, *Bioorg Med Chem Lett*. **20**, 3169–3172 (2010).

10. A. J. Kasabe, P. J. Kasabe, *Int J Pharmacy Pharm Sci*. **2**, 132–135 (2010).

11. R. N. Sharma, K. P. Sharma, S. N. Dixit, (2010).

12. K. Manna, Y. K. Agrawal, *Eur J Med Chem*. **45**, 3831–3839 (2010).

13. T. Taj, R. R. Kamble, T. M. Gireesh, R. K. Hunnur, S. B. Margankop, *Eur J Med Chem*. **46**, 4366–4373 (2011).

14. M. A. Ali *et al.*, *J Enzyme Inhib Med Chem*. **27**, 155–159 (2012).

15. M. J. Ahsan, J. G. Samy, H. Khalilullah, M. A. Bakht, M. Z. Hassan, *Eur J Med Chem*. **46**, 5694–5697 (2011).

16. K. Manna, Y. K. Agrawal, *Medicinal chemistry research*. **20**, 300–306 (2011).

17. S. N. Shelke, G. R. Mhaske, V. D. B. Bonifácio, M. B. Gawande, *Bioorg Med Chem Lett*. **22**, 5727–5730 (2012).

18. K. Hazra *et al.*, *Arch Pharm (Weinheim)*. **345**, 137–146 (2012).

19. D. K. Jain, U. Bhadoriya, J. L. Lodhi, *Asian Journal of Chemistry*. **25**, 1387 (2013).

20. D. N. Rana, M. T. Chhabria, N. K. Shah, P. S. Brahmkshatriya, *Medicinal Chemistry Research*. **23**, 370–381 (2014).

21. A. Ahmad, A. Husain, S. A. Khan, M. Mujeeb, A. Bhandari, *Journal of Saudi Chemical Society*. **20**, 577–584 (2016).

22. D. N. Rana, M. T. Chhabria, N. K. Shah, P. S. Brahmkshatriya, *Medicinal Chemistry Research*. **23**, 2218–2228 (2014).

23. P. N. Kalaria, S. P. Satasia, D. K. Raval, *New Journal of Chemistry*. **38**, 2902–2910 (2014).

24. V. Monga *et al.*, *Medicinal chemistry research*. **23**, 2019–2032 (2014).

25. S. C. Karad, V. B. Purohit, D. K. Raval, *Eur J Med Chem*. **84**, 51–58 (2014).

26. S. R. Deshpande, S. N. Nagrale, M. V Patil, P. S. Chavan, *Indian J Pharm Sci*. **77**, 24 (2015).

27. A. A. Napoleon, F.-R. N. Khan, E. D. Jeong, E. H. Chung, *Chinese Chemical Letters*. **26**, 567–571 (2015).

28. S. D. Joshi *et al.*, *Eur J Med Chem*. **107**, 133–152 (2016).

29. M. S. Muneera, J. Joseph, *J Photochem Photobiol B*. **163**, 57–68 (2016).

30. S. C. Karad, V. B. Purohit, P. Thakor, V. R. Thakkar, D. K. Raval, *Eur J Med Chem*. **112**, 270–279 (2016).

31. A. Solankee, R. Tailor, K. Kapadia, (2016).

32. S. R. Dixit *et al.*, *Open Med Chem J*. **11**, 92 (2017).

33. R. Sadashiva *et al.*, *Medicinal Chemistry Research*. **26**, 1213–1227 (2017).

34. P. V Sowmya *et al.*, *Research on Chemical Intermediates*. **43**, 7399–7422 (2017).

35. C. S. Hallikeri, S. D. Joshi, B. Yenni, S. Dixit, V. H. Kulkarni, *INDIAN JOURNAL OF HETEROCYCLIC CHEMISTRY*. **27**, 17–23 (2017).

36. K. P. Thakor, M. V Lunagariya, B. S. Bhatt, M. N. Patel, *Appl Organomet Chem*. **32**, e4523 (2018).

37. B. V. S. Lokesh, Y. R. Prasad, A. B. Shaik, *Infectious Disorders-Drug Targets (Formerly Current Drug Targets-Infectious Disorders)*. **19**, 310–321 (2019).

38. S. Pola, K. K. Banoth, M. Sankaranarayanan, R. Ummani, A. Garlapati, *Medicinal Chemistry Research*. **29**, 1819–1835 (2020).

39. K. T. Wong *et al.*, *Molecules*. **26**, 2081 (2021).

40. J. Rasgania *et al.*, *J Mol Struct*. **1295**, 136657 (2024).

41. M. Zala *et al.*, *ACS Omega* (2025).

42. R. P. Tailor, K. G. Prajapati, M. A. Isa, A. P. Kappo, *Biochem Biophys Res Commun*, 151358 (2025).

43. Y. Cui *et al.*, *Eur J Med Chem*, 117889 (2025).
